# The global distribution and the risk prediction of relapsing fever group *Borrelia*: a data review with modelling analysis

**DOI:** 10.1016/S2666-5247(23)00396-8

**Published:** 2024-03-08

**Authors:** Tian Tang, Ying Zhu, Yuan-Yuan Zhang, Jin-Jin Chen, Jian-Bo Tian, Qiang Xu, Bao-Gui Jiang, Guo-Lin Wang, Nick Golding, Max L Mehlman, Chen-Long Lv, Simon I Hay, Li-Qun Fang, Wei Liu

**Affiliations:** State Key Laboratory of Pathogen and Biosecurity, Beijing Institute of Microbiology and Epidemiology, Beijing, China; Department of Epidemiology and Biostatistics, School of Public Health, Wuhan University, Wuhan, China; State Key Laboratory of Pathogen and Biosecurity, Beijing Institute of Microbiology and Epidemiology, Beijing, China; State Key Laboratory of Pathogen and Biosecurity, Beijing Institute of Microbiology and Epidemiology, Beijing, China; Department of Epidemiology and Biostatistics, School of Public Health, Wuhan University, Wuhan, China; State Key Laboratory of Pathogen and Biosecurity, Beijing Institute of Microbiology and Epidemiology, Beijing, China; State Key Laboratory of Pathogen and Biosecurity, Beijing Institute of Microbiology and Epidemiology, Beijing, China; State Key Laboratory of Pathogen and Biosecurity, Beijing Institute of Microbiology and Epidemiology, Beijing, China; Telethon Kids Institute, Nedlands, WA, Australia; School of Population Health, Curtin University, Bentley, WA, Australia; Melbourne School of Population and Global Health, University of Melbourne, Parkville, VIC, Australia; Department of Health Metrics Sciences, School of Medicine, University of Washington, Washington, WA, USA; Institute for Health Metrics and Evaluation, University of Washington, Washington, WA, USA; State Key Laboratory of Pathogen and Biosecurity, Beijing Institute of Microbiology and Epidemiology, Beijing, China; Department of Health Metrics Sciences, School of Medicine, University of Washington, Washington, WA, USA; Institute for Health Metrics and Evaluation, University of Washington, Washington, WA, USA; State Key Laboratory of Pathogen and Biosecurity, Beijing Institute of Microbiology and Epidemiology, Beijing, China; State Key Laboratory of Pathogen and Biosecurity, Beijing Institute of Microbiology and Epidemiology, Beijing, China; Department of Epidemiology and Biostatistics, School of Public Health, Wuhan University, Wuhan, China

## Abstract

**Background:**

The recent discovery of emerging relapsing fever group *Borrelia* (RFGB) species, such as *Borrelia miyamotoi*, poses a growing threat to public health. However, the global distribution and associated risk burden of these species remain uncertain. We aimed to map the diversity, distribution, and potential infection risk of RFGB.

**Methods:**

We searched PubMed, Web of Science, GenBank, CNKI, and eLibrary from Jan 1, 1874, to Dec 31, 2022, for published articles without language restriction to extract distribution data for RFGB detection in vectors, animals, and humans, and clinical information about human patients. Only articles documenting RFGB infection events were included in this study, and data for RFGB detection in vectors, animals, or humans were composed into a dataset. We used three machine learning algorithms (boosted regression trees, random forest, and least absolute shrinkage and selection operator logistic regression) to assess the environmental, ecoclimatic, biological, and socioeconomic factors associated with the occurrence of four major RFGB species: *Borrelia miyamotoi*, *Borrelia lonestari*, *Borrelia crocidurae*, and *Borrelia hermsii*; and mapped their worldwide risk level.

**Findings:**

We retrieved 13 959 unique studies, among which 697 met the selection criteria and were used for data extraction. 29 RFGB species have been recorded worldwide, of which 27 have been identified from 63 tick species, 12 from 61 wild animals, and ten from domestic animals. 16 RFGB species caused human infection, with a cumulative count of 26 583 cases reported from Jan 1, 1874, to Dec 31, 2022. *Borrelia recurrentis* (17 084 cases) and *Borrelia persica* (2045 cases) accounted for the highest proportion of human infection. *B miyamotoi* showed the widest distribution among all RFGB, with a predicted environmentally suitable area of 6·92 million km^2^, followed by *B lonestari* (1·69 million km^2^), *B crocidurae* (1·67 million km^2^), and *B hermsii* (1·48 million km^2^). The habitat suitability index of vector ticks and climatic factors, such as the annual mean temperature, have the most significant effect among all predictive models for the geographical distribution of the four major RFGB species.

**Interpretation:**

The predicted high-risk regions are considerably larger than in previous reports. Identification, surveillance, and diagnosis of RFGB infections should be prioritised in high-risk areas, especially within low-income regions.

**Funding:**

National Key Research and Development Program of China.

## Introduction

Relapsing fever is a zoonotic disease caused by relapsing fever group *Borrelia* (RFGB), composed of bacteria belonging to the genus *Borrelia* within the family Borreliaceae in the order Spirochaetales.^1,2^ RFGB, fastidious and slow-growing in culture, is transmitted to animal or human hosts through haematophagous arthropods, including hard (ixodid) ticks, soft (argasid) ticks, and body lice (*Pediculus humanus humanus* and *Pediculus humanus capitis*). Accordingly, two groups of RFGB can be distinguished on the basis of different transmission vectors: tick-borne relapsing fever group and louse-borne relapsing fever group.^3^

Patients infected with RFGB present with recurrent fever accompanied by headache, myalgia, chills, and other non-specific symptoms.^1,4^ Diagnosis of human infection is challenging because there are no specific clinical features that differentiate it from other vector-borne infections, which are associated with morphologically similar transmission vectors to those of RFGB.^5^ Testing thick blood smears from patients at the peak of fever is a common method to confirm RFGB infection; however, this approach is insufficiently sensitive to fully identify all infected cases. Particularly for low-density infections, a delayed diagnosis results in ineffective treatment of patients, leaving them with recurrent febrile symptoms.^2,6^

Since the 21st century, the extensive application of PCR has facilitated laboratory diagnosis, therefore accelerating the discovery of various tick-borne relapsing fever spirochetes, primarily in soft ticks and less often in hard ticks. These discoveries have further complicated the taxonomy of *Borrelia*, with many new species proposed and a third *Borrelia* group identified that does not cluster with either the Lyme disease or relapsing fever clades.^4,7^ Among all known species, *Borrelia miyamotoi* has been increasingly detected in diverse animals and ticks, and poses a significant concern for local and travelling populations as an emerging tick-borne pathogen.^8,9^

RFGB is substantially underdiagnosed owing to insufficient surveillance systems, poor awareness of the disease, and unavailability of clinical diagnostic tools; thus its disease burden and epidemiology are unclear.^6,10^ Relapsing fever caused by different RFGB species is likely to exhibit diversified epidemiological and ecological features, as well as clinical symptoms in patients (apart from recurring fever). There is an urgent and unmet need to present recent epidemiological findings on RFGB.

In this study, we explored the diversity and geographical distribution of RFGB and the multifactorial effects that determine the spatial patterns of the disease. We also predicted the probability of RFGB infection in areas where no historical field investigation has been done, with the aim to provide guidance for future surveillance and prevention strategies targeting relapsing fever.

## Methods

### Database establishment and spatial distribution of RFGB species

We systematically searched PubMed, Web of Science, GenBank, CNKI, and eLibrary for published articles or reports without language restriction from Jan 1, 1874, to Dec 31, 2022. We used the search terms: “relapsing fever *Borrelia*” OR “relapsing fever” OR “tick-borne relapsing fever” OR “louse-borne relapsing fever”. Two authors (TT and J-JC) independently screened the titles and abstracts of all studies for inclusion by using EndNote. For all eligible studies, the full text was retrieved and assessed for eligibility in the same manner. Each of the RFGB species was further searched to identify potentially missed studies, by using the same search strategy as the initial literature search and including taxonomy names.

We excluded the following studies: opinion and editorial articles, media reports, theses, reviews, and abstracts of posters and presentations at conferences or scientific meetings; drug or vaccine trials; experimental transmission or pathogenic mechanism study of RFGB; and studies in which diagnostic methods were not specified or unclearly described (appendix 1 p 13). Articles documenting RFGB natural infection events in vectors, host animals, and humans were included. Confirmed infections were those determined by molecular assay or successful isolation followed by microscopic observation. For serological studies, positive results were included only if they were determined by a four-fold increase in neutralising antibody or a seroconversion between acute and convalescence for human infection. Those determined by ELISAs or indirect fluorescent-antibody test were excluded because of cross-reactivity with other members with close genetic similarity.^10,11^ Detailed criteria for confirmed infections are in appendix 1 (p 14). Disagreements of opinion were resolved through discussions with one author (C-LL). The protocol was registered with PROSPERO and reported according to the Preferred Reporting Items for Systematic Reviews and Meta-Analyses (PRISMA) statement (CRD42022382610; appendix 1 pp 10‒12).^12^

For each validated record of study, we extracted data for 15 types of information, including eight types related to basic study information, three types related to RFGB detection from vectors or animals, and four types related to RFGB detection from humans (appendix 1 p 15). We extracted data for clinical manifestation for all patients with reported clinical information, and clinical factors (age, gender, episodes of fever, blood cell counts, and use of antibiotics) extracted from case reports were used in logistic regression modelling to explore their association with Jarisch-Herxheimer reaction (appendix 1 p 3). Data for the detection of different *Borrelia* species in individual ticks were collected to show co-infection. We obtained data for 44 environmental, ecoclimatic, biological, and socioeconomic variables, which were chosen on the basis of data availability and knowledge of their potential association with the risk of RFGB infection. To standardise the spatial resolution of these variables, we created a global grid map with a resolution of 10×10 km, and mean values for each variable were calculated for each square of the grid(detailed in appendix 1 pp 3‒4 and pp 18‒22). The location with geocoordinates of RFGB occurrence was defined as point occurrence, and others without geocoordinates defined as polygon occurrence. To minimise potential ecological fallacy, we excluded large polygon occurrence records from ecological modelling by using an area cutoff. We used 400 km^2^ and 900 km^2^ as thresholds for those without geocoordinates, respectively, and the polygon occurrence records exceeding these thresholds were excluded from ecological modelling (appendix 1 p 6).

### Niche modelling of the four main RFGB species

On the basis of the number of eligible publications and occurrence locations, the four most common RFGB species (*B miyamotoi*, *Borrelia hermsii*, *Borrelia crocidurae*, and *Borrelia lonestari*) were determined, which were further predicted for their potential distribution by modelling analysis. In the first step, seven main tick vectors (*Ixodes persulcatus, Ixodes ricinus, Ixodes scapularis, Ixodes pacificus*, *Amblyomma americanum*, *Ornithodoros hermsi*, and *Ornithodoros sonrai*) that have been found to be infected with these four RFGB species were identified on the basis of the absolute number and proportion of positive detections (appendix 1 pp 23‒25). Second, we undertook a comprehensive literature search of Web of Science and PubMed between Jan 1, 1980, and Dec 31, 2022, to identify all reports relating to these seven tick species. The search terms used included the names of each tick species. We included the same study types as for the first search; we included reports that provided specific geographic coordinates for sampling location, and excluded those without such information. Additionally, we used the Global Biodiversity Information Facility database and VectorMap to supplement the location data for each tick species (appendix 1 pp 4, 26). The geographic coordinates of each tick species occurrence were mapped onto the global grid map with a resolution of 10×10 km to establish their linkage with these ecological variables. The occurrence grids for each tick species were considered as cases (appendix 2).^13,14^ To verify the effects of different sampling methods for controls on spatial distribution modelling to predict their potential distribution, we compared the classification effects of sampling methods between random grids and background grids on models, and obtained control grids by sampling at a control-to-case ratio of 3:1 within the study areas that were more than 30 km away from case grids (appendix 1 pp 3‒5; 27). Third, the boosted regression trees (BRT) model was fitted 100 times to predict the average probability of occurrence for each tick species using environmental, ecoclimatic, and biological variables as predictors. The average probability for all grids was set as the habitat suitability index (HSI) within the predicted area.^14–16^ The HSIs of the seven predominant tick species were internally validated by the relative uncertainty (the ratio of the 95% uncertainty intervals to the HSI) and externally validated by comparing the suitable habitat with other previously published studies to ensure reliability, before their further application in the niche modelling of RFGB species.*A americanum* was applied in the modelling of *B lonestari; O sonrai* for *B crocidurae; O hermsi* for *B hermsii;* and *I persulcatus*, *I ricinus*, *I scapularis*, and *I pacificus* were used for *Borrelia miyamotoi* (appendix 1 p 5). Finally, the HSIs of these ticks, together with environmental, ecoclimatic, biological, and socioeconomic factors, were used in the niche modelling of four major RFGB species (appendix 3). To explore the relationship between the risk of RFGB occurrences, the HSIs of these ticks, and ecological variables and to predict the potential risk of RFGB occurrences, we used three machine learning models—BRT, random forest (RF), and least absolute shrinkage and selection operator (LASSO) logistic regression—with 10-fold block cross-validation.^17^ We calculated the mean (95% CI) of relative contributions (RCs) and the area-under-curve (AUC) of receiver operating characteristic (ROC) curves over the 100 models to assess predictive performance, and selected the model with highest mean AUC as the final result (appendix 1 pp 5‒6; appendix 4). By overlaying population counts on maps of RFGB-suitable regions, we evaluated both at-risk population size and areas affected by major RFGB species.

### Role of the funding source

The funder of the study had no role in study design, data collection, data analysis, data interpretation, or writing of the report.

## Results

**Figure 1:**
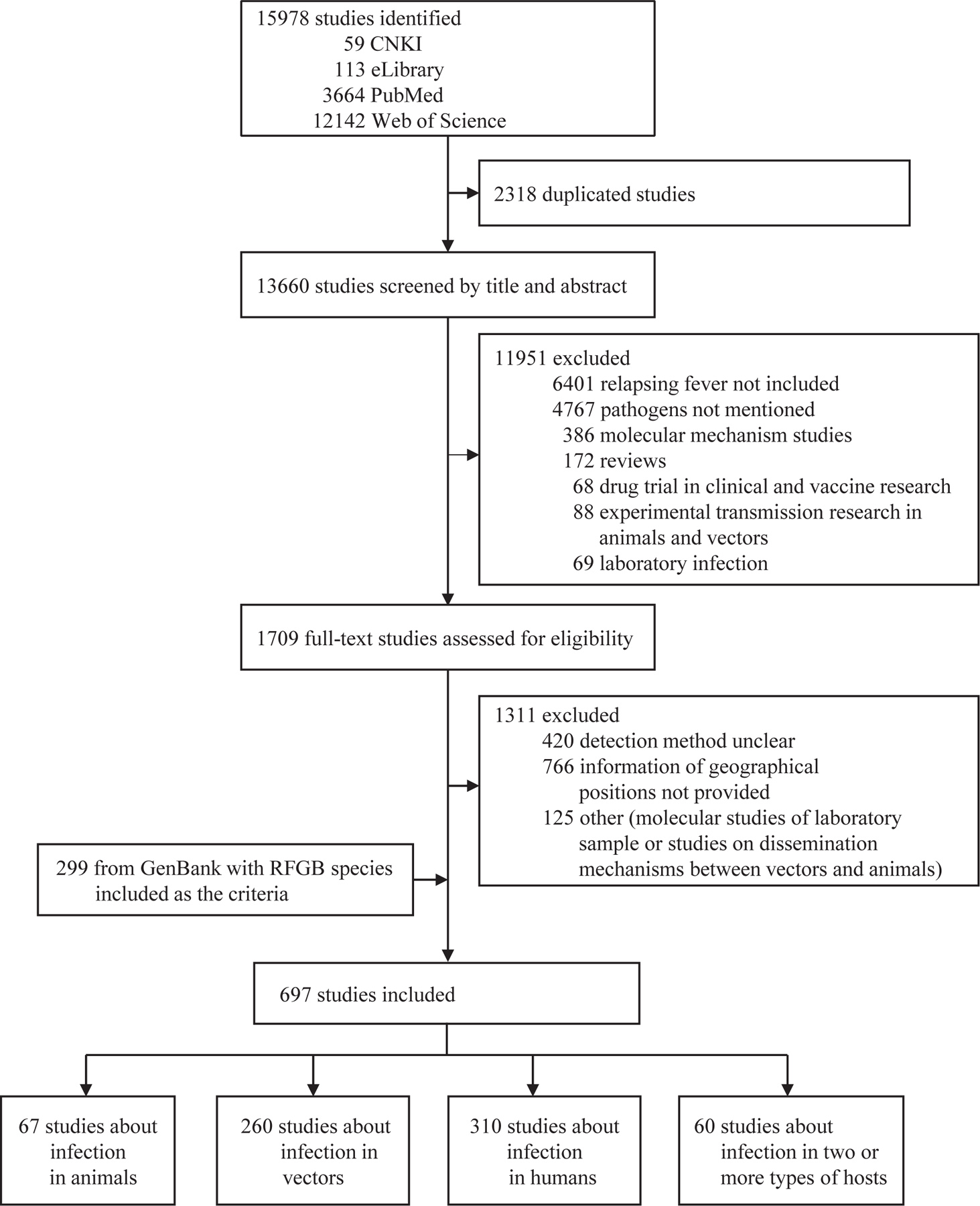
Flow diagram of the literature review RFGB = relapsing fever group Borrelia.

**Figure 2:**
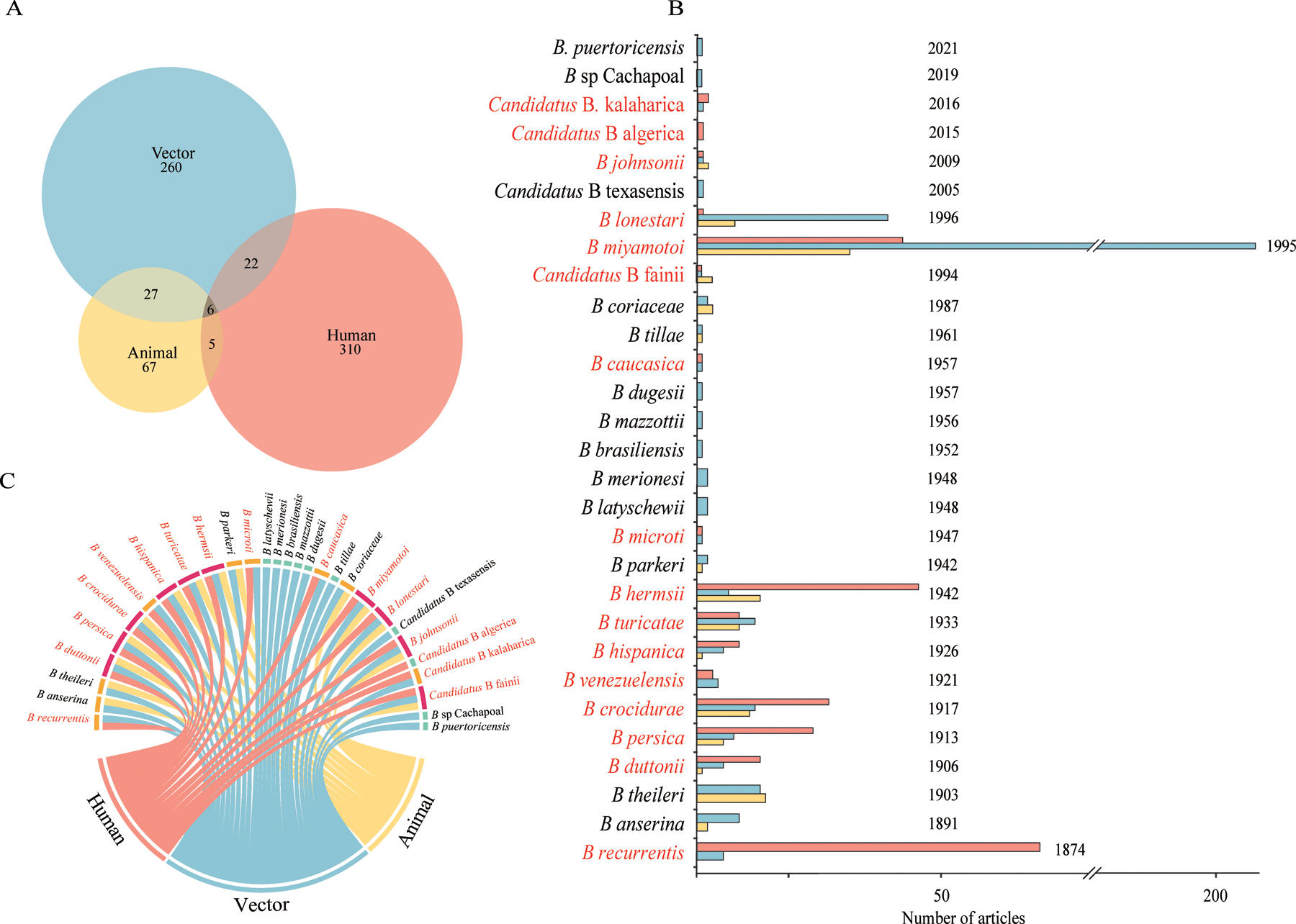
RFGB species infection in animals, vectors, and humans, from publications searched from Jan 1, 1874, to Dec 31, 2022 (A) Overall number of publications on RFGB detection in vectors (blue), animals (yellow), and humans (red). (B) Number of publications on RFGB species stratified by host types with year of first reporting for each RFGB species. (C) Chord diagram between RFGB species and host types. In panels B and C, the names of species with human infection records are marked in red. *B*=*Borrelia.* RFGB=relapsing fever group *Borrelia*.

Our search identified 13 660 articles and 299 reports from GenBank, of which 13 262 were excluded; 697 unique studies were included in the analysis (figure 1). 310 (44%) studies reported RFGB infection in humans, 260 (37%) in arthropod vectors, and 67 (10%) in animals. Additionally, 60 studies reported RFGB in two or more types of hosts, with most involving animals and vectors (n=27), or humans and vectors (n=22; figure 2A). The first reported RFGB was *Borrelia recurrentis* in 1874;^18^ since then and up until the end of 2022, 29 RFGB species had been determined (figure 2B). Of these species, 16 (55%) RFGB were pathogenic to humans. *B miyamotoi* was the most frequently reported species (223 papers), followed by *B recurrentis* (68 papers), *B hermsii* (50 papers), *B lonestari* (40 papers), and *B crocidurae* (38 papers; appendix 1 pp 16‒17). Overall, 19 (66%) of the 29 determined RFGB species were associated with multiple hosts, with four (14%) linked to both arthropod vectors and animals, five (17%) linked to both arthropod vectors and humans, and ten (34%) linked to all three hosts. Nine RFGB species were associated with vectors only, and *Candidatus* Borrelia algerica was detected only in humans (figure 2C).

**Figure 3:**
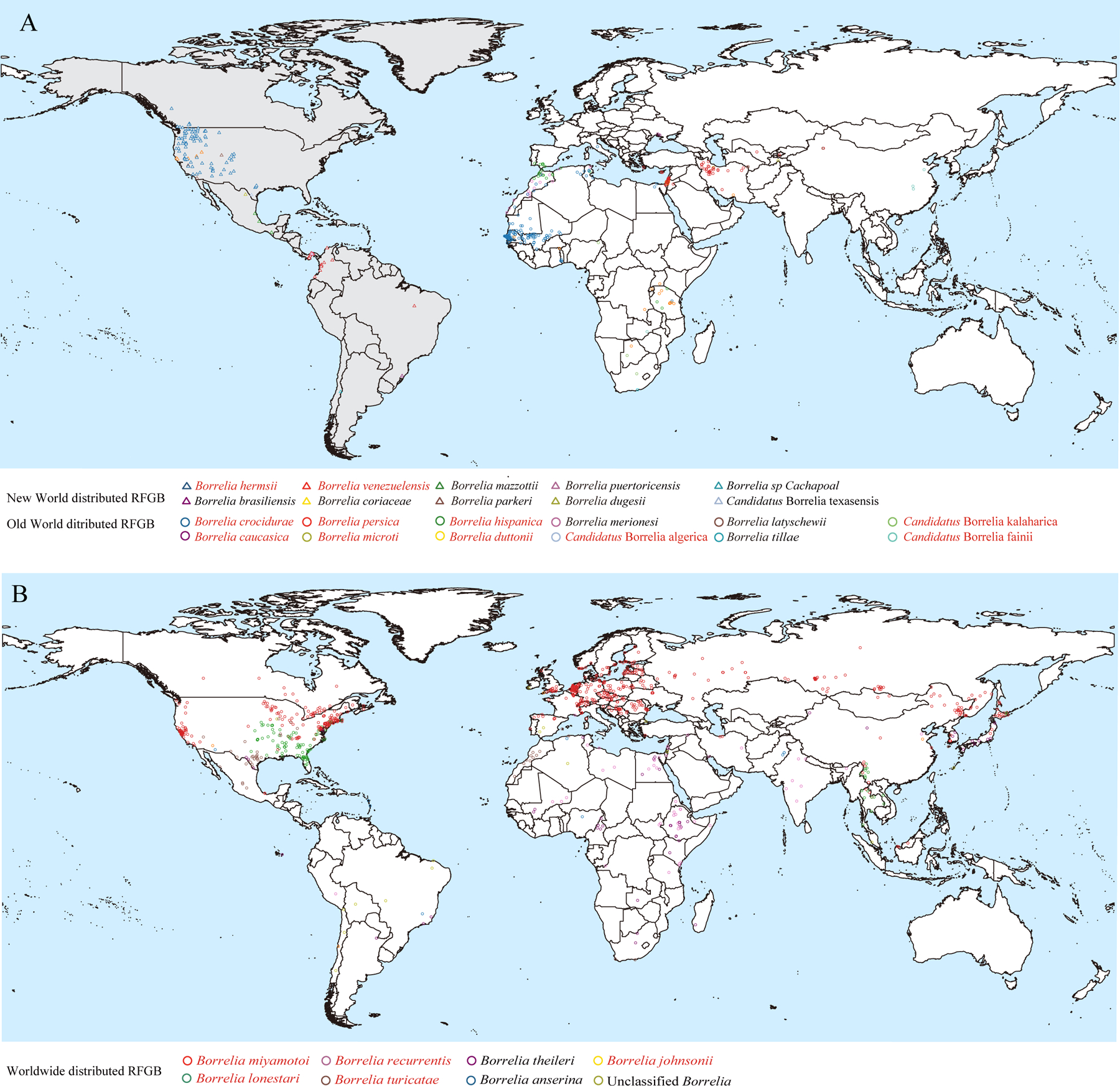
Spatial distribution of RFGB species on a global scale (A) New World or Old World distributed RFGB species. New World countries are marked in grey. (B) RFGB species with worldwide distribution. The names of RFGB species with human infection records are marked in red. RFGB=relapsing fever group *Borrelia*.

On the basis of their spatial distribution, the 29 RFGB species were reclassified into three categories: New World (ten species reported only in the Americas), Old World (12 species reported outside the Americas), and worldwide (seven species distributed globally; figure 3). Among the New World RFGB, *B hermsii* and *Borrelia venezuelensis* were reported in human cases, with *B hermsii* being the most widely distributed, spanning from west coast USA to central USA. Nine of the Old World RFGB were reported to cause human infections, including three widely distributed ones: *Borrelia persica* mainly found in the Middle East, *B crocidurae* in west and north Africa, and *Borrelia hispanica* in north Africa (figure 3A). Five of the globally distributed RFGB species were associated with human infection (*B miyamotoi*, *Borrelia recurrentis*, *B lonestari*, *Borrelia turicatae,* and *Borrelia johnsonii*), all extensively distributed across North America, western Europe, east Asia, southeast Asia, and other northern hemisphere regions (figure 3B). Among all 16 human-pathogenic species, *B miyamotoi* was identified as having the widest distribution, primarily in east Asia, Europe, and both eastern and western coasts of North America.

**Figure 4:**
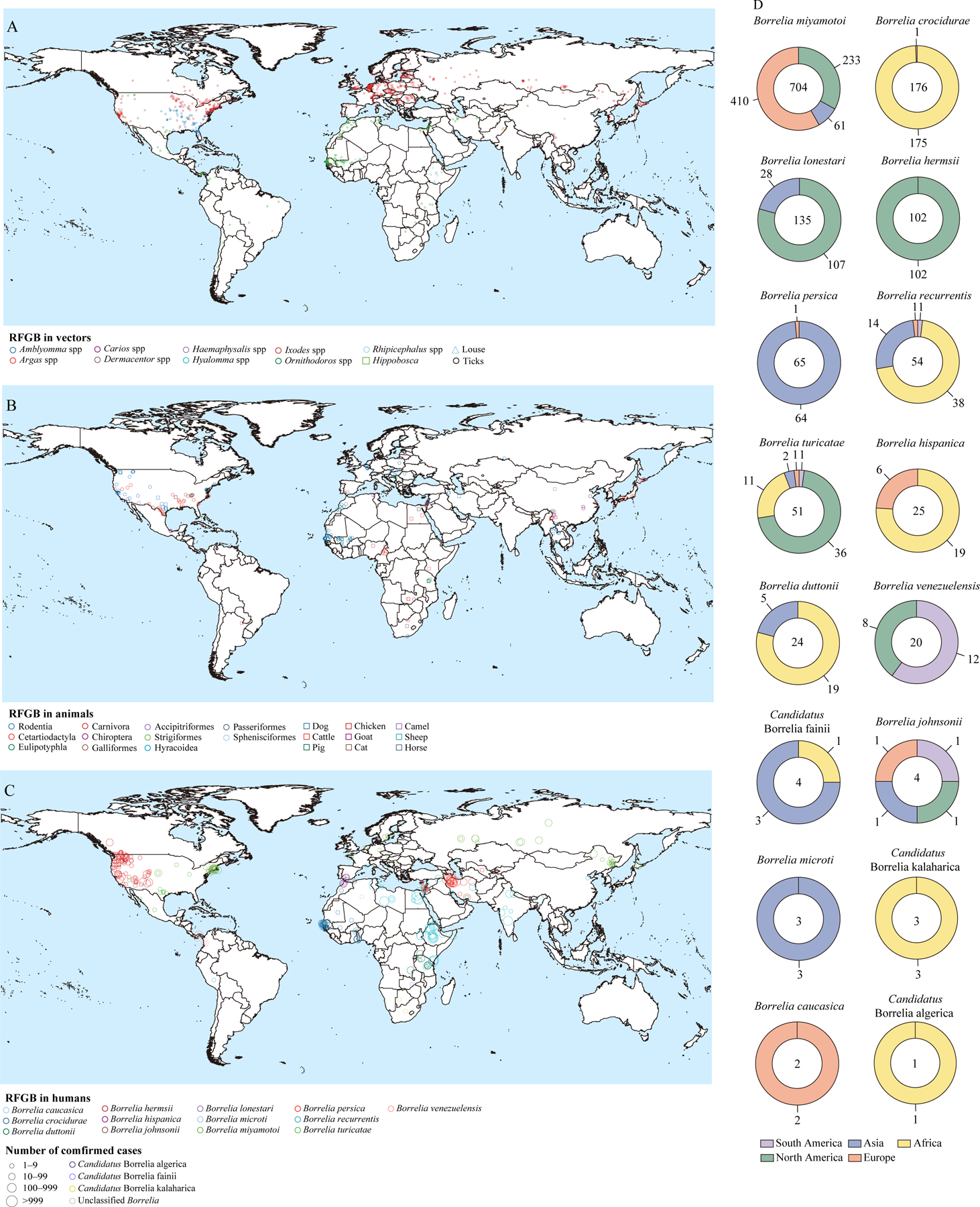
Global distribution of RFGB detection events in vectors and hosts (A) Vectors. (B) Animals. (C) Humans. (D) Number of human and host infection occurrence locations (after removing duplication) for each RFGB species, stratified by continent. RFGB=relapsing fever group *Borrelia*.

The presence of RFGB in *Ixodes* (hard ticks) was predominantly reported north of 30° N and exhibited a wider distribution compared with *Ornithodoros*, found mainly in northwest Africa, and *Amblyomma*, found mainly in North America (figure 4A). RFGB infections in the order Rodentia have been documented worldwide, covering almost all inhabitable continents except for Oceania (figure 4B). Human cases with *B miyamotoi* infection were the most widely distributed and were reported in multiple countries in North America and Eurasia, particularly coastal areas. Infection with this species was followed by *Borrelia recurrentis* infection, which was primarily observed in east Africa, north Africa, and south Asia; *B persica* in the Persian Gulf and the Middle East; *B crocidurae* on the west coast of Africa; and *B hermsii*, which mainly presented on the west coast of North America and central USA. The distribution pattern of human RFGB infections closely mirrored that of RFGB species recorded in their tick vectors (figure 4C). Considering all hosts collectively, the distribution of RFGB species varied between continents—eg, *B lonestari* was observed mainly in North America whereas *B miyamotoi* was found mainly in Europe and North America (figure 4D).

**Figure 5:**
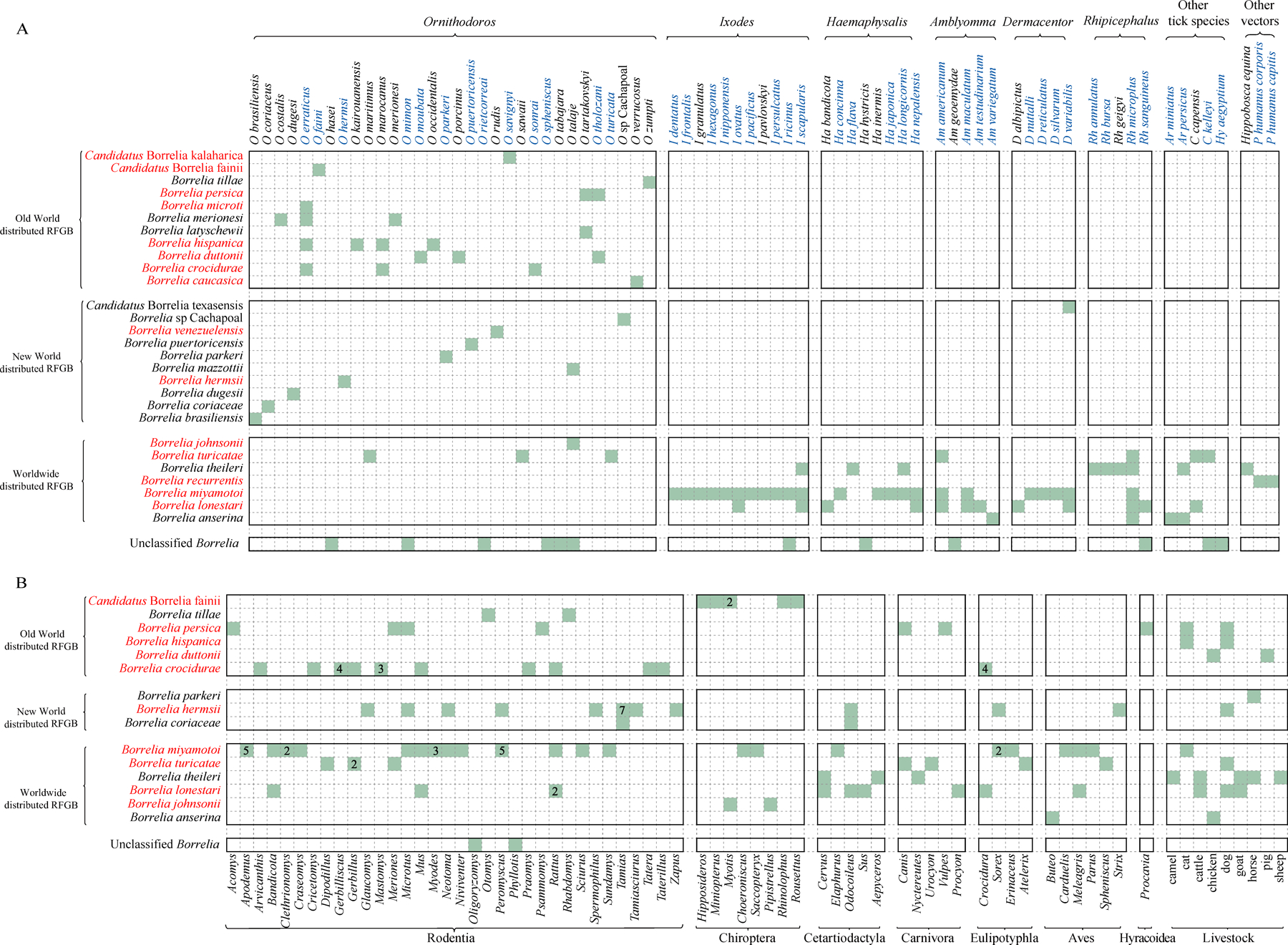
Vectors and numbers of animal species from which each RFGB species was detected (A) RFGB species detected in vectors, including 71 tick species, most of which further classified by genus. Vectors with documented human bites are marked in blue; RFGB species with documented human infections are marked in red. (B) RFGB species detected in animals classified by genus, including wildlife and domestic animals. The total number of animal species in each genus with detected RFGB species is shown inside the matrix when it is greater than 1. The names of RFGB species with human infection records are marked in red. RFGB=relapsing fever group *Borrelia*.

28 RFGB species were identified from arthropod vectors: 27 from ticks, one (B recurrentis) from only lice, and one (Borrelia theileri) from both ticks and the biting fly Hippobosca equina (figure 5A). We identified six RFGB species in 32 hard tick species, and 25 RFGB species in 31 soft tick species. Four species (*B theileri*, *Borrelia anserina*, *B lonestari*, and *B turicatae*) were found to infect both hard ticks and soft ticks. Soft ticks were associated with a higher competency of carrying RFGB than were hard ticks (25 of 31 [81%] vs six of 32 [19%]). Additionally, five hard tick and seven soft tick species were infected by unclassified RFGB, whereas records of human bites were associated with 43 tick species (appendix 1 pp 28‒30). The soft tick *Ornithodoros* was found to be infected with the highest variety of RFGB (22 species), followed by *Rhipicephalus* (five species), *Amblyomma* (four species), *lxodes* (three species), *Haemaphysalis* (three species), and *Dermacentor* (three species). *B miyamotoi* infected the largest number of tick species (23 hard ticks), followed by *B lonestari* (11 hard ticks and one soft tick), *Borrelia theileri* (seven hard ticks and one soft tick), and *B turicatae* (two hard ticks and five soft ticks). Co-infection of RFGB and Lyme disease spirochete was observed for *B miyamotoi* with *Borrelia afzelii*, *Borrelia bavariensis*, *Borrelia burgdorferi*, *Borrelia garinii*, and *Borrelia valaisiana*, which was determined from five tick species (appendix 1 p 31).

12 species of RFGB were identified in 61 genera of wild animals (figure 5B). The order Rodentia, encompassing 68 species in 32 genera, was infected with eight RFGB species (six can infect humans), followed by Chiroptera (three RFGB species detected in ten species, all can infect humans), Eulipotyphla (five RFGB species detected in ten species, all can infect humans), and Aves (five RFGB species detected in seven species, four can infect humans). Nine types of domestic animals were infected with ten RFGB species. Cats were infected with three different RFGB species and dogs with five, all of which can cause human infection. *B miyamotoi* was detected in the greatest number of animal species (34 species), followed by *B crocidurae* (19 species) and *B hermsii* (18 species).

26 583 human cases with confirmed RFGB infection were recorded, including 17 084 with *B recurrentis* infection and 9499 with tick-borne relapsing fever infection (appendix 1 p 32). 4792 (50%) of 9499 tick-borne relapsing fever cases were infected with confirmed species of RFGB, of which *B persica* was the most frequently reported (2045 [43%]), followed by *B crocidurae* (894 [19%]), *B miyamotoi* (825 [17%]), *B hermsii* (458 10%]), *B venezuelensis* (223 [5%]), *Borrelia duttonii *(177 [4%]), and *B hispanica* (150 [3%]). 12 445 patients had available clinical information, including 8974 (72·1%) cases with louse-borne relapsing fever infection (ie, *B recurrentis*) and 3471 (27·9%) cases with tick-borne relapsing fever infection (appendix 1 pp 33‒34). The networks of co-occurring symptoms based on 226 case reports showed that 50 (22%) of 226 cases with fever were accompanied by headache, myalgia, and chills (appendix 1 p 40). The Jarisch-Herxheimer reaction, the most serious side-effect of treatment for relapsing fever, was reported in 38 (17%) of 226 cases (appendix 1, p 35), with its frequency significantly associated with episodes of fever and thrombocytopenia (appendix 1 p 36).

Seven main tick species (*I persulcatus, I ricinus, I scapularis, I pacificus*, *A americanum*, *O hermsi*, and *O sonrai*) were determined for the four major RFGB. The distribution of these tick species was influenced by various ecological factors; climatic covariates had the greatest effect on five of the tick species whereas mammalian richness primarily affected *O hermsi* and leaf area index primarily affected *I persulcatus* (appendix 1 p 37; 41‒47). For each of the seven main tick species, the areas suitable for potential occurrence were estimated on the basis of the HSIs (appendix 1 pp 48‒54). *I persulcatus* was projected to be widely distributed between 45° and 60° N in Eurasia, whereas the three tick species (*I ricinus, I scapularis*, and *I pacificus*) that were associated with *B miyamotoi* were predicted to be distributed in more restricted geographical regions. *A americanum* (vector of *B lonestari*) was predicted in the southeastern coast of North America, *O hermsi* (vector of *B hermsii*) in western USA, and *O sonrai* (vector of *B crocidurae*) in southern Africa.

In the modelling analysis for the four major RFGB species, RF outperformed BRT and LASSO models in terms of predictive performance, as indicated by the mean AUC (appendix 1 p 55). Adequate prediction capacity was obtained for all major RFGB species, with the mean AUC ranging from 0·963 (95% CI 0·918‒0·990) for *B crocidurae* to 0·987 (0·961‒0·998) for *B lonestari* (table) when using 900 km^2^ as the threshold for polygon occurrences. These AUC values showed improved model performance compared with those obtained when using a threshold of 400 km^2^ (appendix 1 p 39). On the basis of the model results, the occurrence of all four major RFGB species was significantly associated with the HSIs of vector ticks, which had the greatest effect among all the evaluated predictors (appendix 1 p 38). The less important contributors varied among species—e.g., the occurrence of *B miyamotoi, B lonestari*, and *B hermsii* was associated with annual mean temperature; the occurrence of *B lonestari* and *B hermsii* was significantly associated with ecoclimatic factors, for which eight ecoclimatic factors individually had RCs exceeding 3%; socioeconomic factors such as human footprint and population density only affected the occurrence of *B miyamotoi*; and rodent richness and mammalian richness were positively associated with the occurrence of *B hermsii* (appendix 1 pp 38; 56‒59).

We predicted the areas and population sizes potentially affected by RFGB (table). *B miyamotoi* is the most widely distributed, with a suitable habitat of 6·92 million km^2^ inhabited by nearly 1·34 billion people, mainly concentrated in Europe and eastern North America (appendix 1 p 60). *B lonestari* is predicted to occur along the southeast coast of North America, covering a range of 1·69 million km^2^. The potential at-risk population size was not evaluated, since controversy remains as to whether *B lonestari* can infect people (appendix 1 p 61). *B hermsii* is predicted to occur in western USA within an area of 1·48 million km^2^ and inhabited by approximately 24·02 million people (appendix 1 p 62). *B crocidurae* exhibits a wide distribution across Africa around 10° N, covering an area of approximately 1·67 million km^2^ where about 341·01 million people reside (appendix 1 p 63).

## Discussion

From this study we have identified 29 RFGB species worldwide, with 27 identified from 63 tick species and 16 known to cause human infections. Several hard ticks, such as *Rhipicephalus* and *Amblyomma* spp, have shown competence as vectors for RFGB transmission. Importantly, multiple tick species from different geographical sources can carry a single RFGB species—eg, *B miyamotoi* was identified in *Dermacentor silvarum*, *Haemaphysalis concinna*, *Haemaphysalis longicornis*, and *I persulcatus*.^9,10,19,20^ Co-infection of RFGB and Lyme spirochete has been reported in individual ticks. All our findings challenge the historical assumption that RFGB can be found only in particular hosts and vectors within a given geographical region.^21^ Historically, RFGB species have been categorised into the Old World (Palearctic-Afrotropic ecozone) and the New World (Nearctic ecozone) borrelia,^4^ and traditionally, RFGB species were believed to be transmitted by soft ticks, apart from *B recurrentis* transmitted by human lice and *B lonestari* and *B miyamotoi* transmitted by hard ticks.^1,21^ In the past 30 years , both the variety and distribution of RFGB species have greatly expanded. Furthermore, there is an increasing number of case reports of louse-borne relapsing fever in the New World, such as in Peru,^22^ in addition to previous reports in Ethiopia. Most recent cases have been reported in European countries, and all were associated with refugees from east Africa and the Middle East.^23^ Still, compared with the small geographical extent of louse-borne relapsing fever infections, tick-borne relapsing fever infections are confirmed to occur in vectors and animals across a wider geographical range.^3^ These expanded perspectives on competent hosts and suitable geographical ranges could contribute towards improved understanding of the tick–pathogen interface and stimulate future research interest.

Seven RFGB species display a global distribution, with five causing human infection (*B miyamotoi*, *B recurrentis*, *B lonestari*, *B turicatae*, and *B johnsonii*). Although the highest case number has been reported from *B recurrentis*, the widest distribution has been reported for *B miyamotoi*. As an emerging species of *Borrelia* transmitted by hard ticks, *B miyamotoi* has been extensively reported to cause relapsing fever in humans across multiple countries in North America, Europe, China, Japan, and the Asian region of Russia.^24^ Among all identified RFGB species, *B miyamotoi* was harboured by the largest number of both tick species and animal species, and was predicted to have the largest environmentally suitable area. The main contributor to this finding was the vector tick HSI, followed by population density and urban built-up land area. We therefore hypothesise that the number of RFGB cases is likely underestimated.^6^ In regions predicted to be at risk, health-care providers should consider the possibility of infection with *B miyamotoi* in patients presenting with relapsing fever accompanied by erythema migrans rash or thrombocytopenia syndrome.^25^ Additionally, *Borrelia* spp associated with soft ticks are transmitted mainly by *Ornithodoros* ticks and thrive in endemic foci in tropical and subtropical latitudes, which were predicted to be expanding northwards.

In niche modelling, the HSIs of the main vector ticks were positively associated with and contributed most to the occurrences of all four major RFGB species. The predicted suitable habitat for most of the tick species was consistent with the aggregated data from previous studies (appendix 1 p 8), except for a wider distribution predicted for *I persulcatus* in southern Finland in our study,^26^ which might be related to more locations recorded for the detection of *I persulcatus* in our database or the selection of grid scale. The establishment of this dataset and associated HSIs can provide valuable insights and guidance for further development of early-warning systems.

There are several limitations of this study. Time ranges of the ecological variables in our modelling analysis are not fully consistent with the recorded RFGB occurrences. Our model applies single static biological variables for assessing environmental features, potentially overlooking or obscuring effects associated with the temporal dynamics of these variables. Additionally, field testing and reporting quality of RFGB infections vary across different countries, particularly in low-income countries, and under-reporting can result in false negatives, leading to an underestimation of the model-estimated risk. Moreover, positive detection of RFGB from vectors or animals does not necessarily indicate transmission or reservoir competence, and these factors should be determined in laboratory studies and considered in future modelling efforts.

In conclusion, this study provides a comprehensive depiction of global distribution and ecological associations pertaining to the four major RFGB species, predicting regions at risk for infection with these pathogens. The modelling results will facilitate targeted surveillance in addition to prevention and control measures across diverse countries and regions worldwide.

## Supplementary Material

Appendix 1

Appendix 2

Appendix 3

Appendix 4

## Figures and Tables

**Table:** Average AUC, areas, and population sizes predicted by the RF models at medium-to-high risk of exposure to the four major RFGB species

	Average testing AUC (95% CI)	Population size (million)					Area (10 000 km^2^)				
	
		Eurasia	Africa	Americas	Oceania	Worldwide	Eurasia	Africa	Americas	Oceania	Worldwide
											

*Borrelia miyamotoi*	0·980 (0·966‒0·990)	1017·21	20·28	301·55	..	1339·04	497·51	1·34	193·57	..	692·42
*Borrelia lonestari* *	0·987 (0·961‒0·998)	..	..	..	..	..	..	..	168·47	..	168·47
*Borrelia hermsii*	0·975 (0·944‒0·999)	..	..	24·02	..	24·02	..	..	148·21	..	148·21
*Borrelia crocidurae*	0·963 (0·918‒0·990)	..	341·01	..	..	341·01	..	167·43	..	..	167·43

AUC=area under the curve. RF=random forest. RFGB=relapsing fever group Borrelia.

* Given that there is controversy about whether *Borrelia lonestari* could infect people, its potential effect on at-risk population size was not evaluated.

## Data Availability

All the data collected in this study are available in the appendices.

## Research in context

### Evidence before this study

On Dec 8, 2022, we undertook a comprehensive search on PubMed, Web of Science, MedRvix, and bioRvix, to identify all studies of relapsing fever group *Borrelia* (RFGB) since the inception of the databases using the search terms (“relapsing fever” OR “relapsing fever *Borrelia*” OR “RF *Borrelia*”) AND (“spatial” OR “distribution”). We did not impose any restrictions on language or publication date. Of the 122 articles found, 88 were related to the detection of RFGB in vectors or animals or humans, and 17 reported experimental or mechanism studies. Among these publications, only 17 described the spatial distribution of RFGB. 13 studies were undertaken within a single country, while four encompassed multiple countries. Notably, among these four studies, one was a meta-analysis of *Borrelia miyamotoi* at the global scale and the other three used ecological modelling studies at a continental scale: one explored the distribution and future risk prediction for *Borrelia hermsii* in North America using maximum entropy model based on ecoclimatic predictors; and the other two separately assessed the effect of ecological factors on tick species known to carry RFGB in the Middle East and north Africa. To date, no study has provided a comprehensive quantification of the global risk of RFGB infection.

### Added value of this study

We undertook an exhaustive search of all published articles to extract data on RFGB detection in vectors, animals, or humans between Jan 1,1874, and Dec 31, 2022. We identified a total of 29 RFGB species, of which 27 were identified from 63 tick species and 16 were known to cause human infection. Although the highest number of cases has been reported from *Borrelia recurrentis*, the widest distribution was identified for *B miyamotoi* in multiple countries. Among all determined RFGB species, *B miyamotoi* was detected in the largest number of both tick and animal species, and was predicted to have the largest environmentally suitable area. The main contributor to this predicted effect was the vector tick habitat suitability index, followed by climatic factors. The soft tick *Ornithodoros* carries the greatest variety of RFGB. Soft ticks had a higher competency in carrying RFGB species than did hard ticks. By applying machine learning models, we estimated the contributions of environmental, ecoclimatic, biological, and socioeconomic factors towards geospatial distribution for the four major RFGB species. We predicted the areas and population sizes at potential risk of infection by these RFGB species.

### Implications of all the available evidence

This study underscores the necessity for RFGB surveillance in the high-risk regions that we identified, primarily in west Africa, south Europe, and east Asia. The capacity of ticks to transmit RFGB might lead to an expansion of the geographical regions with high-risk of relapsing fever. Collectively, our findings could help to guide the diagnosis, surveillance, and control of RFGB infections in high-risk regions.
