# Supplementary material for: The global distribution and the risk prediction of relapsing fever group Borrelia: a data review with modelling analysis
Source: Lancet Microbe. Author manuscript; Available in PMC 2025 Oct 10. (PMC12513746; doi:10.1016/S2666-5247(23)00396-8)
Supplement: Appendix 1 [file NIHMS2109450-supplement-Appendix_1.docx]

**Supplementary appendix**

Supplement to: The global distribution and the risk prediction of relapsing fever group Borrelia: a data review with modelling analysis

**Continent**

[Supplementary Methods 3](#_Toc145101787)

[Supplementary Results 8](#_Toc145101788)

[PRISMA Checklist 10](#_Toc145101789)

[Supplementary table 1: The inclusion and exclusion criteria of screening publications. 13](#_Toc145101790)

[Supplementary table 2: The laboratory tests used to detect RFGB in this study. 14](#_Toc145101791)

[Supplementary table 3: Variables extracted from selected studies. 15](#_Toc145101792)

[Supplementary table 4: The references for all the RFGB species. 16](#_Toc145101793)

[Supplementary table 5: The spatial resolution, study duration and source of the included data. 18](#_Toc145101794)

[Supplementary table 6: Ecological factors potentially associated with the ticks and tick pathogens used in the modelling analysis 19](#_Toc145101794)

[Supplementary table 7: Variables used for ecological modelling in this study. 21](#_Toc145101796)

[Supplementary table 8: The 16 RFGB species with reports of human infection. 23](#_Toc145101797)

[Supplementary table 9: Detection of the four major RFGB species in ticks. 24](#_Toc145101798)

[Supplementary table 10: The number of studies and occurrence locations for the seven tick species. 26](#_Toc145101799)

[Supplementary table 11: Kappa coefficient and F1 score of the BRT models obtained for each tick species with different sampling methods and ratios. 27](#_Toc145101800)

[Supplementary table 12: Vectors with record of RFGB detecting and evidence of biting human 28](#_Toc145101801)

[Supplementary table 13: The coinfection of *Borrelia miyamotoi* with Lyme disease spirochete in ticks. 31](#_Toc145101802)

[Supplementary table 14: The number of human cases with RFGB infection by diagnosis methods. 32](#_Toc145101803)

[Supplementary table 15: Clinical characteristics of human infections with RFGB. 33](#_Toc145101804)

[Supplementary table 16: The demographic features and the main clinical data of 226 case reports. 35](#_Toc145101805)

[Supplementary table 17: The logistic regression analysis of JHR occurrence in relapsing fever cases. 36](#_Toc145101806)

[Supplementary table 18: The RCs of significant contributors to the occurrence of seven main species of ticks based on BRT models. 37](#_Toc145101807)

[Supplementary table 19: The RCs of significant contributors to the spatial distribution of the four major RFGB species based on RF models. 38](#_Toc145101809)

[Supplementary table 20: Comparison of model performance for RFGB niche modelling of the four RFGB species by using different thresholds for polygon occurrences. 39](#_Toc145101811)

[Supplementary figure 1: The networks of co-occurring symptoms in the relapsing fever cases. 40](#_Toc145101812)

[Supplementary figure 2: Effects of major predictors (RCs >3%) for presence of *Ixodes persulcatus* based on BRT models. 41](#_Toc145101814)

[Supplementary figure 3: Effects of major predictors (RCs >3%) for presence of *Ixodes ricinus* based on BRT models. 42](#_Toc145101815)

[Supplementary figure 4: Effects of major predictors (RCs >3%) for presence of *Ixodes scapularis* based on BRT models. 43](#_Toc145101816)

[Supplementary figure 5: Effects of major predictors (RCs >3%) for presence of *Ixodes pacificus* based on BRT models. 44](#_Toc145101817)

[Supplementary figure 6: Effects of major predictors (RCs >3%) for presence of *Amblyomma americanum* based on BRT models. 45](#_Toc145101818)

[Supplementary figure 7: Effects of major predictors (RCs >3%) for presence of *Ornithodoros hermsi* based on BRT models. 46](#_Toc145101819)

[Supplementary figure 8: Effects of major predictors (RCs >3%) for presence of *Ornithodoros sonrai* based on BRT models. 47](#_Toc145101820)

[Supplementary figure 9: The predicted and recorded distributions of *Ixodes persulcatus* in Eurasia 48](#_Toc145101821)

[Supplementary figure 10: The regional predicted and recorded distributions of *Ixodes ricinus*. 49](#_Toc145101822)

[Supplementary figure 11: The predicted and recorded distributions of *Ixodes scapularis* in North America. 50](#_Toc145101823)

[Supplementary figure 12: The predicted and recorded distributions of *Ixodes pacificus* in North America. 51](#_Toc145101824)

[Supplementary figure 13: The predicted and recorded distributions of *Amblyomma americanum* in North America. 52](#_Toc145101825)

[Supplementary figure 14: The predicted and recorded distributions of *Ornithodoros hermsi* in North America. 53](#_Toc145101826)

[Supplementary figure 15: The predicted and recorded distributions of *Ornithodoros sonrai* in Africa. 54](#_Toc145101827)

[Supplementary figure 16: Predictive performance of the three machine-learning algorithms. 55](#_Toc145101828)

[Supplementary figure 17: Effects of major predictors (RCs >3%) for presence of *Borrelia miyamotoi* based on RF models. 56](#_Toc145101831)

[Supplementary figure 18: Effects of major predictors (RCs >3%) for presence of *Borrelia lonestari* based on RF models. 57](#_Toc145101832)

[Supplementary figure 19: Effects of major predictors (RCs >3%) for presence of *Borrelia hermsii* based on RF models. 58](#_Toc145101833)

[Supplementary figure 20: Effects of major predictors (RCs >3%) for presence of *Borrelia crocidurae* based on RF models. 59](#_Toc145101834)

[Supplementary figure 21: The predicted and recorded distributions of *Borrelia miyamotoi* within global range. 60](#_Toc145101835)

[Supplementary figure 22: The predicted and recorded distributions of *Borrelia lonestari* in North America. 61](#_Toc145101836)

[Supplementary figure 23: The predicted and recorded distributions of *Borrelia hermsii* in North America. 62](#_Toc145101837)

[Supplementary figure 24: The predicted and recorded distributions of *Borrelia crocidurae* in Africa. 63](#_Toc145101838)

Supplementary Reference 64

# Supplementary Methods

*Data extraction*

For the review of relapsing fever group *Borrelia* (RFGB) related studies, we extracted the information on RFGB species and compared them with the records in the NCBI. The confirmed RFGB species were searched again using the same method used for relapsing fever, and all the studies were removed for duplication by EndNote (version 19.1, <https://endnote.com/>) to form the literature database. Studies that met our inclusion criteria were added to the RFGB database.

The following data were extracted from each selected article about RFGB: article title, authors, publication year, study period referred as the time when samples were collected, study site (latitude and longitude or up to the highest resolution), species of RFGB, detection method, type of vectors and animal to the species level, total number and positive number of tested samples. The laboratory-confirmed infections were limited to those determined by molecular assays, isolation followed by microscopic identification, or isolation followed by serological assays. For the positive results determined by serological assays, we used only those determined by the neutralizing antibody assay.

*Clinical spectrum of* RFGB

For the RFGB species with ≥10 human cases that have reported symptoms, we extracted data on the clinical information from the database. For each RFGB species, the frequency and prevalence of each symptom were recorded. As it is likely that the common symptoms are more likely to be reported while the others tend to be neglected, those unreported symptoms were estimated in two ways: (I) recorded as zero, assuming the symptom is absent; (II) recorded with the same frequency as the minimum level among all the reported symptoms for this specific RFGB species in the same study. By applying this strategy, we obtained a lower bound and an upper bound on the frequency of each symptom. We defined those symptoms with the lower bound ≥30% as the major symptom. The network of co-occurring symptoms across the RGB species was established by programming in R 4.0.3 (R Foundation for Statistical Computing, Vienna, Austria). Based on the database, a total of 226 case reports were collected, with the details of the patients extracted, i.e., medical history, physical examination results, pathogenic findings, treatment process, course of disease and outcome (detailed in appendix 1 p 35). The logistic regression model was performed to examine the factors (including age, gender, episodes of fever, blood cell counts and usage of antibiotics) associated with the occurrence of Jarisch-Heyxheimer reaction (JHR). We summarized the clinical characteristics of all RFGB species and presented the networks of co-occurring symptoms in the relapsing fever cases through text association analysis (appendix 1 p 40).

*Geo-positioning of the occurrence data*

For each RFGB species, an occurrence was defined as one or more confirmed infection(s) at a unique location (geo-coordinates, polygons, or 10 km × 10 km pixels), regardless of the type of the hosts. To gain the locations of RFGB occurrences, we extracted the geo-coordinates from peer-reviewed articles reporting confirmed RFGB occurrences (detected by molecular assay or pathogen isolation) as point occurrence. When point information was not available, we extracted the smallest areal unit (county, city or province) as polygon occurrence. For each polygon occurrence, the coordinates of its geographic centroid were queried from Google Maps. All locations were geo-positioned with the highest possible accuracy and checked to ensure that the coordinates were accurate and duplicates were removed. Results from serological testing in humans were excluded from the modelling analyses, and the details of the selection criteria were supplemented in appendix 1 p 13.

*Assembling occurrence data and covariates*

We created a global grid-map with a resolution of 10 km × 10 km using ArcGIS 10.7 (Esri Inc, Redlands, CA, USA) and then associated each grid with ecological variables. To balance the availability of global data and their priori importance of predicting RFGB when selecting variables for modelling, we have pre-confirmed its potential association with the risk of RFGB infection (appendix 1 pp 18‒20). The map in raster format was summarized into a processable data format at the study level based on the grid-map, and the R package “Raster” was used for resampling in order to change the resolution of raster data. Specifically, for land cover data, percentage coverage of 13 types were extracted and summarized at 10 km × 10 km resolution. For climatic data, 19 variables (Bio1‒19) were created based on monthly maximum temperature, monthly minimum temperature, and monthly rainfall from 1980‒2019 using the R package “dismo” in order to generate more biologically meaningful variables. For other variables, we used the “aggregate” function to create a new raster with a 10 km resolution, and then used the “projectRaster” function to project the values of environmental variables to the grid-map through a bilinear interpolation method. Each occurrence was matched to the grid-map according to its coordinates. For a polygon occurrence record, we assigned the grid containing the centroid of the polygon as the occurrence grid. To minimize potential ecological fallacy, we first excluded all huge polygon occurrence records from ecological modelling due to insufficient resolution.^1,2^ For polygon occurrence records with an area no larger than 900 km^2^, we calculated the mean of each ecological variable across all grids within the polygon and associated the mean value with the occurrence grids, that was, the grids containing the centroid of the polygon. We divided the research time into the following four segments to match the collected independent variables: 1980‒1989, 1990‒1999, 2000‒2009, 2010‒2019, and independent variables outside the time range were taken from the most recent year. If multiple records were associated with the same grids, the occurrences were recorded by the segments of research time.

We compiled data on 44 ecological variables potentially associated with RFGB occurrence (appendix 1 pp 19‒20).^1,3,4^ These variables were categorized into four types: 19 ecoclimatic variables, 15 environmental variables, 7 biological variables, and three socioeconomic variables. All variables were processed in grid-map at the 10 km resolution. Ecoclimatic, environmental, and biological variables have been widely used to predict the distribution of tick species and the risk of tick-borne diseases. The increased frequency of tick-borne disease was also associated with complex socioeconomic factors, which may expand the human-animal-tick-environment interface as well as influence the process of the pathogen discovery and monitoring.^5,6^ We calculated the mean of each ecological covariate across each grid within the four time periods (appendix 1 pp 21‒22).

*Niche modelling of the main tick species*

We performed a systematic search of PubMed and Web of Science for published studies or reports on *Amblyomma americanum, Ixodes persulcatus, Ixodes ricinus, Ixodes scapularis, Ixodes pacificus, Ornithodoros sonrai*, and *Ornithodoros hermsi* without any language restrictions from January 1980 to December 2022. Search results were exported through EndNote (version 19.1), duplicates were removed, and a total of 17 930 studies were retained and imported into Microsoft Excel 2019. The studies that met the inclusion criteria for screening publications were further supplemented in the RFGB database.

The spatial data associated with tick collection was obtained for those that met the following criteria: (I) study on pathogens in ticks providing locations of sampling; (II) confirmation of tick species with molecular evidence or identification of morphology; (III) providing accurate locations of tick collection or geographic distribution figures. All the polygon occurrences corresponding to administrative units above the county level were excluded from the niche modelling. Details of the geographic information database are provided in appendix 2. The location information for each tick species was also retrieved from the database of the Global Biodiversity Information Facility (GBIF) and VectorMap. The accuracy of the coordinates was checked through Google Earth, and some samples with apparent errors in their geographic location coordinates were removed. After the above screening process, the geographical coordinates of each tick species were assembled and overlapped on a global grid of 10 km × 10 km pixels, and only one code of grid was retained (appendix 1 p 26).

Based on the distribution information of tick species in appendix 2, *A. americanum*, *I. scapularis*, *I. pacificus* and *O. hermsi* were only found in North America. *O. sonrai* was merely found in Africa and *I. persulcatus* was widely distributed in Eurasia. These six tick species were fitted in niche modelling on the continental plates scale to obtain the habitat suitability index. It was notable that *I. ricinus* occurred in the Europe, Western Asia and Northern Africa plates around the Mediterranean Sea, especially in Europe. To completely contain the distribution area of *I. ricinus*, we expanded region of prediction to longitudes of -14°W‒67°E, latitude of 27°N‒72°N, and finally fitted niche modelling in this mask area. The habitat suitability index of *A. americanum,* *O. hermsi* and *O. sonrai* would be used as a predictor for the niche modelling of *Borrelia lonestari*, *Borrelia hermsii, and Borrelia crocidurae* respectively. For *Borrelia miyamotoi*, the indexes of its four main tick species including *I. scapularis*, *I. pacificus* *I. persulcatus* and *I. ricinus* were integrated as follows: (I) in the region where the four tick species had not been reported, the value was set to zero; (II) otherwise, the value was the maximum of the four ticks’ habitat suitability indexes.

All occurrence grids extracted from appendix 2 of each tick species were considered as “cases”. For each occurrence grids, we sampled the pseudo-absence grids as “controls” with a control-to-case ratio of 3:1 for the subsequent modelling. The sampling was restricted to grids within the study area that were more than 30 km away from “case” grids with the method as follows: (1) randomly sampled 10000 grids from the sampling area as the candidate “controls”; (2) randomly sampled the pseudo-absence locations from the candidate “controls” at a ratio of 3:1 around the occurrence locations.^3,7,8^ The boosted regression trees (BRT) model was used to calculate the habitat suitability index for each of the seven main tick species in the corresponding occurrence areas, with the selected 41 variables excepting socioeconomic variables as candidate predictors (appendix 1 pp 21‒22).

To verify the effect of different sampling methods on the modelling results, we extracted and cleaned all data on the tick species collected from GBIF, VectorMap and studies, resulting in more than 100 000 records of point data. The grids in which these point data were located served as the candidate "controls" for each tick species within the study area. The sampling was restricted to grids within the study area that were more than 30 km away from “case” grids with the method as follows: (1) randomly sampled 10000 grids from the candidate “controls”; (2) randomly sampled the pseudo-absence locations from the candidate “controls” at different ratio range of three to five times around the occurrence locations. The modelling scheme remained the same.

By comparing the Kappa coefficient and F1 Score of niche modelling in each tick species, the method with the best classification for most tick species was the method of randomly sampling to obtain "controls" with a ratio of 3:1 within the sampling range, so this sampling method was applied to our modelling (appendix 1 p 27).

*Validation of tick model predictions*

To confirm the accuracy of the tick niche modelling, we performed two methods to validate the predicted habitat suitability index (HSI) for each tick species. The first method was internal validation, which evaluated the robustness of the model itself by calculating the relative uncertainty of the predicted distribution for each tick species. The relative uncertainty for each grid was computed as the ratio of the 95% uncertainty intervals to the predicted suitability. The second method was external validation, which demonstrated the accuracy of the model by comparing it with other previously published studies. We conducted an exhaustive search for the publications on the seven main tick species, and six of which had previously been studied for their predicted distributions. The results extracted from these peer-reviewed studies were compared with our predictions, focusing on the distributional extent of the high-risk areas.

*Niche modelling of four major RFGB species*

To explore the relationship between the risk of RFGB occurrences and ecoclimatic, environmental, biological, and socioeconomic variables, three machine-learning models, including BRT, random forest (RF) and least absolute shrinkage and selection operator (LASSO) logistic regression, were performed and compared to obtain the best predictive performance. The pseudo-absence locations were randomly sampled around the locations of RFGB events which were considered as “cases”, with a range of all the grids except for the grids within 30 km around the “cases” using the method as follows: (I) randomly sampled 10000 grids from 10 km × 10 km pixels map as the candidate “controls”; (II) randomly sampled the pseudo-absence locations from the candidate “controls” at a ratio of 3:1. We then sampled 80% of the training set and 20% of the test set via random splitting and fitted the three models separately, which was repeated 100 times. The machine learning model with the largest average area under the curve (AUC) over 100 repeats was selected to calculate the relative contribution (RCs) of the major predictors and make a final prediction for the risk of RFGB occurrence. All variables were fitted in the three models for niche modelling, but only those variables with an average RC greater than 3% were shown. The optimal threshold value used for the final prediction of the presence or absence of the RFGB species in the study region was based on the Youden index derived from the average AUC over the 100 models. To minimize potential ecological fallacy, we must exclude large polygon occurrence records from ecological modelling using an area cutoff. After calculating the areas of all collected polygon occurrences in our database, a threshold of 900 km^2^ (the minimum sampling range of pseudo-absence is set to 30 km) was finally determined, after taking into account the preservation of more polygon occurrence records (204/316) and controlling the ecological fallacy. And a higher area cutoff may make the average conditions in some areas unable to reasonably reflect conditions where the observation was actually made. Therefore, we have constructed a comparison model with another threshold of 400 km^2^ (the minimum sampling range of pseudo-absence is set to 20 km), i.e., the polygon occurrence records with an area over 400 km^2^ were excluded from ecological modelling.

*(1) BRT model*

The hyperparameters of the BRT model were set by grid search to obtain the best-fit effect. In order to avoid underestimation of error and inappropriate selection of models, we implemented a 10-fold block cross-validation approach for the niche modelling using the package “blockCV”.^9^ We compared the mean residuals of the spatial autocorrelation for different block sizes and determined 300 km as the optimal value for the block. The modelling dataset was divided into different blocks in the raster map by spatial autocorrelation and then randomly encoded, with each raster within a block assigned a unique code. The train and test datasets were segmented by these codes, and the optimal modelling parameters were determined by the best AUC obtained from 10-fold block cross-validation. We then fitted an initial model for each tick (or RFGB) species to obtain the best number of tree and apply in the subsequent modelling. In the model, we randomly divided the data into an 80% training set and a 20% test set and fitted a BRT model, which was repeated 100 times to obtain 100 models based on the 100 resampled training sets for each target tick (or RFGB) species.^1,3,10^ Using these presence and pseudo-absence locations and ecological predictors, BRT models were fitted using the “gbm.step” function in “dismo” package in R.

*(2) Random forest*

Random forest (RF) is another classical ensemble learning model widely used. The training algorithm for RF is based on bootstrap aggregating. Each tree is trained on many bootstrap samples and then evaluated using the remaining data to produce more accurate classifications. The unknown class of an observation will be calculated by the majority vote of the out-of-bag predictions for that observation. We optimized the hyperparameters by grid search with a 10-fold block cross-validation process to avoid inappropriate model selection.^11^ The hyperparameter selection process is the same as in BRT. The R packages “randomForest” were used to develop the random forest model.

*(3) LASSO regression*

We used L1-penalized least absolute shrinkage and selection regression for multivariate analysis, augmented with 10-fold block cross validation for internal validation. This is a logistic regression model that penalizes the absolute size of the coefficients, where the sum of absolute values of coefficients is multiplied by a weight coefficient λ and then added to the traditional loss function. With larger penalties, the estimates of the weaker factors shrink towards zero, so that only the strongest predictors remain in the model. The optimal λ was chosen via 10-fold block cross validation to minimize the average misclassification error. Subsequently, variables identified by least absolute shrinkage and selection operator (LASSO) regression analysis were entered into traditional logistic regression models without penalty (as there is no predictor of more interest than others, double selection was not performed).^12^ The package “glmnet” in R was used to perform the LASSO regression, and optimal λ was chosen using the cv. glmnet function.

*(4) Model evaluation*

Similar to the BRT, we obtained 100 models as a model assembly for RF and LASSO as well as by randomly splitting the data into training and test sets. The RCs of all predictors and the area under the curves (AUCs) for test sets were averaged over the 100 models in the assembly to represent the final estimation results and predictive performance of the model assembly. We selected the best algorithm in terms of the highest average test AUC to map the global distribution of RFGB species. To determine model-predicted high-risk areas for each RFGB species, we chose a cut-off value that maximizes sensitivity + specificity along the average receiver operating characteristic (ROC) curve of the model assembly of the chosen algorithm.^13,14^ Grids with an average predicted probability (over the 100 models) above the cut-off value were considered as having a high risk of presence of the corresponding RFGB species. For each species, the area and population size of the model-predicted high-risk areas were calculated.

**Supplementary references**

1. Zhang YY, Fang LQ, Yang Y, et al. Mapping the global distribution of spotted fever group rickettsiae: a systematic review with modelling analysis. *Lancet Digit Health* 2023; **5:** e5‒15.
2. Sage KM, Johnson TL, Schwan TG, et al. Ecological niche modeling and distribution of *Ornithodoros hermsi* associated with tick-borne relapsing fever in western North America. *PLoS Negl Trop Dis* 2017; **11:** e0006047.
3. Zhao GP, Wang YX, Fan ZW, et al. Mapping ticks and tick-borne pathogens in China. *Nat Commun* 2021; **12:** 1075.
4. Allen T, Murray KA, Zambrana-Torrelio C, et al. Global hotspots and correlates of emerging zoonotic diseases. *Nat Commun* 2017; **8:** 1124.
5. Miao D, Liu W, Fang LQ, et al. Mapping the global potential transmission hotspots for severe fever with thrombocytopenia syndrome by machine learning methods. *Emerg Microbes Infect* 2020; **9:** 817‒26.
6. Magalhães AR, Codeço CT, Svenning JC, Escobar LE, Van de Vuurst P, Gonçalves-Souza T. Neglected tropical diseases risk correlates with poverty and early ecosystem destruction. *Infect Dis Poverty* 2023; **12:** 32.
7. Barbet-Massin M, Jiguet F, Albert CH, Thuiller W. Selecting pseudo‐absences for species distribution models: how, where and how many? *Methods Ecol Evol* 2012; **3:** 327‒38.
8. Vanderwal J, Shoo L, Graham C, et al. Selecting pseudo-absence data for presence-only distribution modelling: How far should you stray from what you know? *Ecol Modell* 2009; **220:** 589‒94.
9. Valavi R, Elith J, Lahoz-Monfort JJ, Guillera-Arroita G. BLOCKCV: An R package for generating spatially or environmentally separated folds for k-fold cross-validation of species distribution models. *Methods Ecol Evol* 2019; **10:** 225‒32.
10. Wang T, Fan ZW, Ji Y, et al. Mapping the distributions of mosquitoes and mosquito‒borne arboviruses in China. *Viruses* 2022; **14:** 691.
11. Walter M, Vogelgesang JR, Rubel F, Brugger K. Tick-borne encephalitis virus and its European distribution in ticks and endothermic mammals. *Microorganisms* 2020; **8:** 1065.
12. Liang W, Liang H, Ou L, et al. Development and validation of a clinical risk score to predict the occurrence of critical illness in hospitalized patients with COVID-19. *JAMA* 2020; **180:** 1081‒89.
13. Schisterman EF, Perkins NJ, Liu A, Bondell H. Optimal cut-point and its corresponding Youden Index to discriminate individuals using pooled blood samples. *Epidemiology* 2005; **16:** 73‒81.
14. Ruopp MD, Perkins NJ, Whitcomb BW, Schisterman EF. Youden Index and optimal cut-point estimated from observations affected by a lower limit of detection. *Biom J* 2008; **50:** 419‒30.

# Supplementary Results

*Symptom characteristics of different RFGB infections*

Based on 374 patients infected with *Borrelia miyamotoi*, in addition to fever, the most common clinical symptoms included headaches, chills, and myalgia, possibly accompanied by some specific symptoms such as erythema migrans. 339 patients infected with *Borrelia hermsii* also presented significant influenza-like symptoms, as well as more frequent vomit, nausea and other gastrointestinal symptoms, with ocular symptoms (eye ache and red eyes) as specific symptoms, while 147 *Borrelia duttonii* infections most frequently showed diarrhea, with febrile convulsion and conjunctivitis as accompanied specific symptoms. In contrast to other species of TBRF infection, 137 patients infected with *Borrelia hispanica* showed more significant thrombocytopenia disorder, and 414 patients infected with *Borrelia crocidurae* presented higher frequency of headaches and weakness. LBRF infections had no prominent clinical symptoms other than fever (detailed in appendix 1 pp 33‒34).

*Ecological associations of vector tick occurrences*

The ecological niches of vector ticks are affected by environmental, ecoclimatic, and biological features. Among them, ecoclimates are the main associated factors of the habitat suitability index (HSI) for most species of vector ticks, but their impacts vary with tick species (appendix 1 p 37). For example, the distribution of *I. ricinus* and *A. americanum* is well-suitable to areas with annual mean temperature from 0 to 20 ℃, while *O. hermsi* is widespread in the areas with annual mean temperature above 20 ℃. Only the *I. persulcatus*, *I. ricinus* and *I. scapularis* are affected by the land cover factors, with the urban built-up land being the most predominant factor affecting their distribution. Isothermality is significantly associated with the HSI for most species of major vector ticks, with the exception of *I. ricinus* and *I. pacificus*, for which, in contrast, annual precipitation merely affects the distribution of *I. ricinus*. The HSI of *O. sonrai* is significantly affected by elevation, while that of *O. hermsi* is affected by mammalian richness, with a rapid increase in the frequency of tick occurrence followed by a stabilization when the richness index exceeds a threshold.

*Validation of tick niche modelling*

The relationship between relative uncertainty and the habitat suitability for each grid on tick species was mapped, indicating that the predicted results are more robust in the areas with higher HSI (appendix 1 pp 48‒54). Moreover, in agreement with the study by Alkishe et al.,^1^ suitable habitat for *I. ricinus* is concentrated in western and southern Europe, the southern Nordic region, and a small portion of North Africa and the Middle East. The suitable habitat predicted by our niche modelling for *I. scapularis*, *I. pacificus* and *A. americanum* are also similar with previous studies.^2,3,4^ In comparison to the study by Wang et al., our model performs better in East Asia for the prediction of suitable habitat for *I. persulcatus*.^5^ Unlike the study of Sage et al.,^6^ our niche modelling performes on a global grid-map with a resolution of 10 km × 10 km, using a database containing only point coordinates of *O. hermsi* and incorporating more independent variables, contributing to the relatively larger extent of areas with higher predicted suitability. Ecological modelling studies of *O. sonrai* are still insufficient.

*Ecological associations of RFGB occurrences*

By applying BRT, RF and LASSO, we have identified appropriate predictive models for each major RFGB and yielded their ecological niches. The predominant factor affecting the distribution of RFGB is the habitat suitability index of its main vector ticks and shows a significantly positive correlation (appendix 1 p 38). Moreover, areas with more urban built-up land, greater population density, and higher human footprint are more suitable for the survival of *Borrelia miyamotoi*. Changes in precipitation and temperature both significantly affect the presence of *Borrelia lonestari,* while the distribution of *Borrelia hermsii* is more sensitive to the variation of temperature. Mammalian richness and rodent richness are also positively associated with the occurrence of *Borrelia hermsii*, but the association decreases significantly in areas with mammalian richness greater than 60. Increased elevation is negatively associated with the distribution of *Borrelia crocidurae*, which is the opposite of the relationship between *Borrelia crocidurae* and human footprint. In addition, *Borrelia crocidurae* has a more suitable habitat in western Africa on the Old-World continent, while *Borrelia hermsii* exhibits a high risk along the western coast of North America on the New-World continent. Areas of high risk for *Borrelia miyamotoi* are concentrated on East Asia, Europe and the east and west coasts of North America, with scattered distributions throughout north of 30°N latitude.

**Supplementary references**

1. Alkishe AA, Peterson AT, Samy AM. Climate change influences on the potential geographic distribution of the disease vector tick *Ixodes ricinus*. *PLoS One* 2017; **12:** e0189092.
2. Ma D, Lun X, Li C, et al. Predicting the potential global distribution of *Amblyomma americanum* (Acari: Ixodidae) under near current and future climatic conditions, using the Maximum Entropy Model. *Biology* 2021; **10:** 1057.
3. Zhang L, Ma D, Li C, Zhou R, Wang J, Liu Q. Projecting the potential distribution areas of *Ixodes scapularis* (Acari: Ixodidae) driven by climate change. *Biology* 2022; **11:**107.
4. Eisen RJ, Feirer S, Padgett KA, et al. Modeling climate suitability of the western blacklegged tick in California. *J Med Entomol* 2018; **55:** 1133‒42.
5. Wang SS, Liu JY, Wang BY, et al. Geographical distribution of *Ixodes persulcatus* and associated pathogens: Analysis of integrated data from a China field survey and global published data. *One Health* 2023; **16:** 100508.
6. Sage KM, Johnson TL, Schwan TG, et al. Ecological niche modeling and distribution of *Ornithodoros hermsi* associated with tick-borne relapsing fever in western North America. *PLoS Negl Trop Dis* 2017; **11:** e0006047.

# PRISMA Checklist

We followed the Preferred Reporting Items for Systematic Reviews and Meta-analysis (PRISMA) statement (S1 PRISMA Checklist). The protocol was registered with PROSPERO (CRD42022382610).

| **Section and Topic** | **Item #** | **Checklist item** | **Location where item is reported** |
| --- | --- | --- | --- |
| **TITLE** | | |  |
| Title | 1 | Identify the report as a systematic review. | 1 |
| **ABSTRACT** | | |  |
| Abstract | 2 | See the PRISMA 2020 for Abstracts checklist. | N/A |
| **INTRODUCTION** | | |  |
| Rationale | 3 | Describe the rationale for the review in the context of existing knowledge. | N/A |
| Objectives | 4 | Provide an explicit statement of the objective(s) or question(s) the review addresses. | N/A |
| **METHODS** | | |  |
| Eligibility criteria | 5 | Specify the inclusion and exclusion criteria for the review and how studies were grouped for the syntheses. | 6 |
| Information sources | 6 | Specify all databases, registers, websites, organisations, reference lists and other sources searched or consulted to identify studies. Specify the date when each source was last searched or consulted. | 6 |
| Search strategy | 7 | Present the full search strategies for all databases, registers and websites, including any filters and limits used. | 6 |
| Selection process | 8 | Specify the methods used to decide whether a study met the inclusion criteria of the review, including how many reviewers screened each record and each report retrieved, whether they worked independently, and if applicable, details of automation tools used in the process. | 6 |
| Data collection process | 9 | Specify the methods used to collect data from reports, including how many reviewers collected data from each report, whether they worked independently, any processes for obtaining or confirming data from study investigators, and if applicable, details of automation tools used in the process. | 6 |
| Data items | 10a | List and define all outcomes for which data were sought. Specify whether all results that were compatible with each outcome domain in each study were sought (e.g. for all measures, time points, analyses), and if not, the methods used to decide which results to collect. | 6 |
|  | 10b | List and define all other variables for which data were sought (e.g. participant and intervention characteristics, funding sources). Describe any assumptions made about any missing or unclear information. | 7 |
| Study risk of bias assessment | 11 | Specify the methods used to assess risk of bias in the included studies, including details of the tool(s) used, how many reviewers assessed each study and whether they worked independently, and if applicable, details of automation tools used in the process. | 7 |
| Effect measures | 12 | Specify for each outcome the effect measure(s) (e.g. risk ratio, mean difference) used in the synthesis or presentation of results. | 8 |
| Synthesis methods | 13a | Describe the processes used to decide which studies were eligible for each synthesis (e.g. tabulating the study intervention characteristics and comparing against the planned groups for each synthesis (item #5)). | N/A |
|  | 13b | Describe any methods required to prepare the data for presentation or synthesis, such as handling of missing summary statistics, or data conversions. | N/A |
|  | 13c | Describe any methods used to tabulate or visually display results of individual studies and syntheses. | N/A |
|  | 13d | Describe any methods used to synthesize results and provide a rationale for the choice(s). If meta-analysis was performed, describe the model(s), method(s) to identify the presence and extent of statistical heterogeneity, and software package(s) used. | N/A |
|  | 13e | Describe any methods used to explore possible causes of heterogeneity among study results (e.g. subgroup analysis, meta-regression). | N/A |
|  | 13f | Describe any sensitivity analyses conducted to assess robustness of the synthesized results. | N/A |
| Reporting bias assessment | 14 | Describe any methods used to assess risk of bias due to missing results in a synthesis (arising from reporting biases). | N/A |
| Certainty assessment | 15 | Describe any methods used to assess certainty (or confidence) in the body of evidence for an outcome. | N/A |
| **RESULTS** | | |  |
| Study selection | 16a | Describe the results of the search and selection process, from the number of records identified in the search to the number of studies included in the review, ideally using a flow diagram. | 8 |
|  | 16b | Cite studies that might appear to meet the inclusion criteria, but which were excluded, and explain why they were excluded. | N/A |
| Study characteristics | 17 | Cite each included study and present its characteristics. | N/A |
| Risk of bias in studies | 18 | Present assessments of risk of bias for each included study. | N/A |
| Results of individual studies | 19 | For all outcomes, present, for each study: (a) summary statistics for each group (where appropriate) and (b) an effect estimate and its precision (e.g. confidence/credible interval), ideally using structured tables or plots. | 8–11 |
| Results of syntheses | 20a | For each synthesis, briefly summarise the characteristics and risk of bias among contributing studies. | N/A |
|  | 20b | Present results of all statistical syntheses conducted. If meta-analysis was done, present for each the summary estimate and its precision (e.g. confidence/credible interval) and measures of statistical heterogeneity. If comparing groups, describe the direction of the effect. | 8–11 |
|  | 20c | Present results of all investigations of possible causes of heterogeneity among study results. | N/A |
|  | 20d | Present results of all sensitivity analyses conducted to assess the robustness of the synthesized results. | N/A |
| Reporting biases | 21 | Present assessments of risk of bias due to missing results (arising from reporting biases) for each synthesis assessed. | N/A |
| Certainty of evidence | 22 | Present assessments of certainty (or confidence) in the body of evidence for each outcome assessed. | N/A |
| **DISCUSSION** | | |  |
| Discussion | 23a | Provide a general interpretation of the results in the context of other evidence. | 13–15 |
|  | 23b | Discuss any limitations of the evidence included in the review. | 15 |
|  | 23c | Discuss any limitations of the review processes used. | 15 |
|  | 23d | Discuss implications of the results for practice, policy, and future research. | 15 |
| **OTHER INFORMATION** | | |  |
| Registration and protocol | 24a | Provide registration information for the review, including register name and registration number, or state that the review was not registered. | 6 |
|  | 24b | Indicate where the review protocol can be accessed, or state that a protocol was not prepared. | 6 |
|  | 24c | Describe and explain any amendments to information provided at registration or in the protocol. | 6 |
| Support | 25 | Describe sources of financial or non-financial support for the review, and the role of the funders or sponsors in the review. | 16 |
| Competing interests | 26 | Declare any competing interests of review authors. | 16 |
| Availability of data, code and other materials | 27 | Report which of the following are publicly available and where they can be found: template data collection forms; data extracted from included studies; data used for all analyses; analytic code; any other materials used in the review. | S1 File,  S2 File,  S3 File,  S4 File. |

# Supplementary table 1: The inclusion and exclusion criteria of screening publications.

| **Criteria** | **Guidance** | **Outcome** |
| --- | --- | --- |
| **Title/Abstract screening** |  |  |
| #1: Disease | Does the Title/Abstract relate to disease of relapsing fever? | If Yes, remain and evaluate #2. If No, exclude. |
| #2: Pathogens | Does the Title/Abstract refer the pathogens which are detected from natural environment? | If Yes, remain and evaluate #3. If No, exclude. |
| #3: Tested objects | Does the Title/Abstract refer the pathogens which are detected from vectors, animals, or humans? | If Yes, remain and evaluate #4. If No, exclude. |
| #4: Not review | Does the Title/Abstract refer the article which is Not a review? (Not presenting new primary data) | If No, remain for full text review. If Yes, exclude. |
| **Full text screening** |  |  |
| #1: Re-screening | Does the article meet the screening criteria following? 1-pathogens belong to RFGB 2-infection in natural environment 3-detected from vectors, animals, or humans 4-not drug or vaccine trials 5-not research of transmission mechanism in vectors and animals 6-not molecular research of RFGB | If Yes, remain and evaluate #2. If No, exclude. |
| #2: Laboratory method | Does the article refer the specific detection methods? 1-detailed specimen used for testing (vectors or blood from animals or humans) 2- testing method for pathogens (microscopical, serological or molecular) 3- pathogen identified in the detection | If Yes, remain and evaluate #3. If No, exclude. |
| #3: Geographical information | Does the article provide the geographical information? 1-geographic location information at administrative division levels 2-exact locations or marked the latitude and longitude 3-explicit locations of getting infections or sampling | If Yes, remain for data set.  If No, exclude. |

RFGB, relapsing fever group *Borrelia*.

# Supplementary table 2: The laboratory tests used to detect RFGB in this study.

|  | **Detection methods** |
| --- | --- |
| **Detection in vectors** | (1) molecular detection with PCR or sequencing;  (2) isolation and cultivation of pathogens from samples;  (3) morphology identification under microscope. |
| **Detection in animals** | (1) molecular detection with PCR or sequencing;  (2) isolation and cultivation of pathogens from samples;  (3) morphology identification under microscope. |
| **Confirmed human cases** | (1) molecular detection and sequence determination;  (2) isolation and cultivation of pathogens from samples;  (3) morphology identification under microscope;  (4) a four-fold increase in titer of specific antibodies in blood sera. |
| **Serological tests in humans** | The seroconversion of RFGB specific antibody from acute phase to convalescent phase in patients with clinical diagnosis of relapsing fever. |

RFGB, relapsing fever group *Borrelia*; PCR, Polymerase Chain Reaction.

# Supplementary table 3: Variables extracted from selected studies.

| **Variables** | **Explanation** |
| --- | --- |
| **Basic information** |  |
| Reference ID | Unique identifier assigned to an article. |
| Article title | Article title that included in the review. |
| Authors | All Authors of the included article. |
| Publication time | Publication years and month of the included article. |
| Study period | The start and end year for the researches. |
| Study site | Locations of sampling at different level including country, the detailed address and explicit coordinates. The geographic center of the detailed locations was used if coordinates are not provided by author. |
| Detection method | molecular assay by PCR; isolation and cultivation; morphology identification; serological assay |
| RFGB species | Standard taxonomy term of RFGB tested in the study. |
| **RFGB detection from vectors/animals** |  |
| Species of vectors/animals | Vectors/animals species that underwent test |
| Number of tested all | Total number of samples that underwent test for specific pathogen. |
| Number of tested positive | Number of samples tested positive for specific pathogen. |
| **RFGB detection from human beings** |  |
| Study type | Case report; Case series; Surveillance; Outbreak events. |
| Number of tested individuals | Number of individuals tested for specific pathogen. |
| Number of individuals tested positive | Number of individuals tested as positive for specific pathogen. |
| Clinical manifestation | Number of human cases reporting each of the clinical symptom |

RFGB, relapsing fever group *Borrelia*.

# Supplementary table 4: The references for all the RFGB species.

| **RFGB species** | **Number of studies (n)** | **Reference ID** |
| --- | --- | --- |
| *Borrelia miyamotoi* | 223 | 6, 9, 13, 15, 17, 22, 32, 45, 63, 67, 73, 78, 82, 83, 84, 89, 93, 96, 113, 127, 128, 130, 133, 134, 135, 138, 140, 141, 145, 147, 148, 149, 150, 151, 152, 153, 158, 159, 160, 161, 162, 163, 164, 168, 171, 175, 177, 178, 179, 181, 184, 185, 187, 189, 191, 192, 193, 198, 199, 200, 201, 203, 207, 208, 209, 211, 214, 217, 223, 224, 225, 227, 228, 233, 234, 236, 240, 241, 243, 244, 249, 251, 253, 255, 256, 257, 260, 262, 263, 264, 266, 270, 273, 279, 282, 283, 284, 289, 295, 296, 300, 301, 302, 303, 305, 306, 310, 311, 313, 314, 315, 317, 318, 323, 324, 325, 327, 328, 330, 332, 334, 335, 336, 338, 340, 343, 344, 345, 346, 349, 350, 352, 353, 354, 356, 357, 358, 359, 360, 366, 375, 381, 385, 386, 390, 392, 394, 395, 396, 405, 407, 415, 420, 429, 431, 437, 438, 463, 464, 467, 471, 472, 473, 474, 475, 476, 479, 481, 482, 491, 493, 494, 501, 502, 519, 520, 522, 524, 525, 527, 529, 531, 533, 535, 539, 540, 542, 543, 551, 553, 554, 555, 558, 573, 574, 577, 578, 599, 606, 607, 608, 609, 611, 612, 619, 621, 622, 624, 625, 626, 627, 630, 633, 634, 636, 637, 649, 655, 656, 658, 668, 680, 691 |
| *Borrelia recurrentis* | 68 | 10, 12, 16, 24, 28, 29, 34, 35, 40, 42, 46, 51, 58, 60, 61, 62, 64, 86, 88, 91, 104,106, 108, 115, 120, 121, 124, 131, 143, 157, 170, 176, 196, 197, 212, 215, 218,220, 226, 230, 245, 247, 248, 254, 265, 326, 363, 364, 365, 368, 376, 401, 406,424, 426, 430, 433, 448, 450, 456, 470, 478, 483, 605, 617, 659, 662, 664 |
| *Borrelia hermsii* | 50 | 3, 14, 19, 30, 31, 33, 48, 52, 53, 76, 82, 85, 87, 94, 114, 118, 119, 123, 142, 146, 221, 222, 250, 286, 293, 299, 316, 322, 329, 374, 377, 380, 413, 421, 440, 452, 453, 454, 468, 469, 485, 492, 536, 552, 567, 569, 570, 598, 620, 682 |
| *Borrelia lonestari* | 40 | 79, 137, 150, 154, 155, 156, 180, 190, 213, 219, 225, 237, 290, 304, 309, 347, 348, 372, 402, 414, 418, 419, 422, 441, 544, 546, 576, 610, 629, 653, 658, 661, 667, 669, 670, 671, 672, 679, 683, 685 |
| *Borrelia crocidurae* | 38 | 8, 20, 21, 44, 47, 49, 56, 65, 72, 81, 90, 101, 111, 122, 139, 144, 271, 274, 281, 292, 312, 361, 370, 382, 397, 398, 399, 427, 428, 498, 528, 530, 560, 561, 566, 582, 597, 623 |
| *Borrelia persica* | 31 | 4, 23, 26, 27, 55, 70, 80, 107, 126, 172, 231, 246, 268, 285, 341, 355, 362, 367, 388, 432, 436, 439, 460, 477, 545, 565, 580, 586, 602, 603, 689 |
| *Borrelia turicatae* | 26 | 2, 7, 25, 36, 77, 103, 125, 169, 205, 277, 351, 384, 397, 421, 435, 447, 505, 508, 511, 538, 541, 568, 579, 644, 645, 666 |
| *Borrelia theileri* | 23 | 41, 186, 259, 339, 404, 512, 517, 518, 562, 639, 640, 641, 642, 643, 646, 647, 650, 651, 652, 654, 673, 674, 686 |
| *Borrelia duttonii* | 18 | 68, 74, 75, 81, 194, 239, 393, 425, 444, 459, 478, 572, 582, 584, 593, 614, 618, 676 |
| *Borrelia hispanica* | 14 | 44, 100, 105, 144, 166, 182, 397, 489, 515, 537, 538, 560, 575, 632 |
| *Borrelia anserina* | 9 | 267, 319, 404, 461, 496, 559, 675, 681, 687 |
| *Borrelia coriaceae* | 5 | 221, 222, 588, 628, 665 |
| *Borrelia johnsonii* | 4 | 82, 383, 510, 690 |
| *Borrelia parkeri* | 3 | 216, 369, 484 |
| *Borrelia venezuelensis* | 3 | 98, 112, 272, |
| *Candidatus* Borrelia fainii | 3 | 202, 337, 648 |
| *Candidatus* Borrelia kalaharica | 3 | 480, 532, 548 |
| *Borrelia* *latyschewii* | 2 | 495, 689 |
| *Borrelia merionesi* | 2 | 144,397 |
| *Borrelia microti* | 2 | 23, 490 |
| *Borrelia dugesii* | 1 | 694 |
| *Borrelia puertoricensis* | 1 | 547 |
| *Borrelia mazzottii* | 1 | 695 |
| *Borrelia tillae* | 1 | 635 |
| *Borrelia brasiliensis* | 1 | 487 |
| *Borrelia caucasica* | 1 | 465 |
| *Borrelia* sp. Cachapoal | 1 | 510 |
| *Candidatus* Borrelia texasensis | 1 | 677 |
| *Candidatus* Borrelia algerica | 1 | 557 |

RFGB, relapsing fever group *Borrelia*.

# Supplementary table 5: The spatial resolution, study duration and source of the included data.

| **Variable** | **Spatial resolution** | **Study duration** | **Source of data** | **Website** | **Reference** |
| --- | --- | --- | --- | --- | --- |
| Climate data | 0°2·5′ | 1975‒2019 | WorldClim | https://www.worldclim.org/ | Fick SE, Hijmans RJ. WorldClim 2: new 1-km spatial resolution climate surfaces for global land areas. Int. J. Climatol., 2017; 37: 4302-15.  Harris I, Jones PD, Osborn TJ, Lister DH. Updated high-resolution grids of monthly climatic observations – the CRU TS3.10 Dataset. Int. J. Climatol., 2014; 34: 623-42. |
| Leaf area index | 8km | 1981‒2019 | Resource and Environment Science and Data Center | https://www.resdc.cn/ | Yang L, Liu R, Chen JM. Retrospective retrieval of long-term consistent global leaf area index (1981-2011) from combined AVHRR and MODIS data. J Geophys Res Biogeosci, 2015; 117. |
| Land cover | 0·3km | 1992‒2019 | European Space Agency | https://maps.elie.ucl.ac.be/CCI/ | European Space Agency. ESA Land Cover Climate Change Initiative (Land_Cover_cci): Global Land Cover Maps, Version 2.0.7. https://catalogue.ceda.ac.uk/uuid/b382ebe6679d44b8b0e68ea4ef4b701c/ (accessed May 28, 2021) |
| Human Footprint | 1km | 2000‒2018 | Scientific Data | https://www.gisrsdata.com/ | Mu, Haowei; Li, Xuecao; Wen, Yanan; Huang, Jianxi; Du, Peijun; Su, Wei; et al. (2021): An annual global terrestrial Human Footprint dataset from 2000 to 2018. figshare. Figure. https://doi.org/10.6084/m9.figshare.16571064.v5 |
| Elevation | 1km | 2010 | EarthEnv (DEM90) | http://www.earthenv.org/ | Robinson N, Regetz J, Guralnick RP. EarthEnv-DEM90: A nearly-global, void-free, multi-scale smoothed, 90m digital elevation model from fused ASTER and SRTM data. ISPRS, 2014; 87: 57-67. |
| Livestock density | 1km | 2010; 2015 | Food and Agriculture Organization (FAO) | http://www.fao.org/livestock-systems/en/ | Gilbert M, Nicolas G, Cinardi G, et al. Global distribution data for cattle, buffaloes, horses, sheep, goats, pigs, chickens and ducks in 2010. Sci Data, 2018; 5: 180227. |
| Mammalian richness | 0°0′30″ | 2013 | International Union for Conservation of Nature (IUCN) | https://sedac.ciesin.columbia.edu/ | International Union for Conservation of Nature - IUCN, and Center for International Earth Science Information Network - CIESIN - Columbia University. 2015. Gridded Species Distribution: Global Mammal Richness Grids, 2015 Release. Palisades, NY: NASA Socioeconomic Data and Applications Center (SEDAC). |
| Rodent richness | 10km | 2018 | BiodiversityMapping.org | https://biodiversitymapping.org/index.php/permissions/ | Jenkins, C.N. & K. Van Houtan. (2016). Global and regional priorities for marine biodiversity protection. Pimm, SL, CN Jenkins, R Abell, TM Brooks, JL Gittleman, LN Joppa, PH Raven, CM Roberts, JO Sexton (2014) The biodiversity of species and their rates of extinction, distribution, and protection. Science 344(6187): 1246752. |
| Population number | 1km | 2020 | WorldPop 2020 | https://www.worldpop.org/ | WorldPop. Population counts, unconstrained global mosaics 2000-2020 (1 km resolution), 2020. https://www.worldpop.org/geodata/listing?id=64/ (accessed Apr 12, 2021). |
| Global downscaled GDP | 0°2·5′ | 1990, 2025 | NASA Socioeconomic Data and Applications Center (SEDAC) | https://sedac.ciesin.columbia.edu/ | Gaffin SR, Xing X, Yetman G. Global 15 x 15 Minute Grids of the Downscaled GDP Based on the SRES B2 Scenario, 1990 and 2025. Palisades, NY: NASA Socioeconomic Data and Applications Center (SEDAC); 2004. |

# GDP, gross domestic product.Supplementary table 6: Ecological factors potentially associated with the ticks and tick pathogens used in the modelling analysis.

| **Variable** | **Reference** | **Content** | **Method** | **Usage and/or result** |
| --- | --- | --- | --- | --- |
| BIO1‒19 | Kylie Sage et al. [1] | RFGB and tick in America | Model: MaxEnt | Used in the RFGB and tick ecological niche model. |
|  | Alkishe AA., et al. [2] | Tick in Europe | Model: MaxEnt | Used in the tick ecological niche model. |
|  | Zhang L, et.al. [3] | Tick in America | Model: MaxEnt | Precipitation of the driest month contributed 19.6% in the model. |
|  | Eisen RJ, et al. [4] | Tick in America | Model: Logistic regression, RF and MaxEnt | Precipitation of coldest quarter contributed 37.8‒70.3 in the model. |
|  | Wang SS, et.al. [5] | Tick in global scale | Model: MaxEnt | Used in the tick ecological niche model. |
| Leaf area index | Fuller T, et.al. [6] | Monkeypox disease in the Congo Basin | Model: Logistic regression and MaxEnt | Used in the monkeypox ecological niche model. |
|  | Zhang YY, et.al. [7] | Tick-borne pathogens in global scale | Model: Logistic regression and RF | Used in tick-borne pathogens ecological niche model. |
| Elevation | Kylie Sage et al. [1] | RFGB and tick in America | Model: MaxEnt | Contributed 18.1% in the model, and elevation (1700m) could most affect monkeypox ecological suitability. |
|  | Miao D, et.al. [8] | Tick and tick-borne pathogens in China | Model: Logistic regression | Used in tick ecological niche model. |
| Mixed cropland and nature vegetation | Allen T, et.al. [9] | Emerging zoonotic diseases in global scale | Model: Logistic regression | Land cover was used in the analysis of zoonotic pathogens ecological niche model. |
| Shrubland | Allen T, et.al. [9] | Emerging zoonotic diseases in global scale | Model: Logistic regression | Land cover was used in the analysis of zoonotic pathogens ecological niche model. |
| Mixed tree, shrub and herbaceous | Allen T, et.al. [9] | Emerging zoonotic diseases in global scale | Model: Logistic regression | Land cover was used in the analysis of zoonotic pathogens ecological niche model. |
| Grassland | Allen T, et.al. [9] | Emerging zoonotic diseases in global scale | Model: Logistic regression | Land cover was used in the analysis of zoonotic pathogens ecological niche model. |
| Sparse vegetation land | Allen T, et.al. [9] | Emerging zoonotic diseases in global scale | Model: Logistic regression | Land cover was used in the analysis of zoonotic pathogens ecological niche model. |
| Flooded vegetation | Allen T, et.al. [9] | Emerging zoonotic diseases in global scale | Model: Logistic regression | Land cover was used in the analysis of zoonotic pathogens ecological niche model. |
| Bare areas | Allen T, et.al. [9] | Emerging zoonotic diseases in global scale | Model: Logistic regression | Land cover was used in the analysis of zoonotic pathogens ecological niche model. |
| Water body | Allen T, et.al. [9] | Emerging zoonotic diseases in global scale | Model: Logistic regression | Land cover was used in the analysis of zoonotic pathogens ecological niche model. |
| Cropland | Allen T, et.al. [9] | Emerging zoonotic diseases in global scale | Model: Logistic regression | Land cover was used in the analysis of zoonotic pathogens ecological niche model. |
| Urban construction land | Allen T, et.al. [9] | Emerging zoonotic diseases in global scale | Model: Logistic regression | Land cover was used in the analysis of zoonotic pathogens ecological niche model. |
| Population count | Miao D, et.al. [8] | Tick and tick-borne pathogens in China | Model: Logistic regression | Contributed 19.7% in the model |
|  | Allen T, et.al. [9] | Emerging zoonotic diseases in global scale | Model: Logistic regression | Used in the pathogens ecological niche model. |
|  | Jones KE, et al. [10] | Emerging disease in global scale | Model: Logistic regression | Used in the pathogens ecological niche model. |
| Global Downscaled GDP | Miao D, et.al. [11] | Tick and tick-borne pathogens in China | Model: Logistic regression | Used in tick ecological niche model. |
|  | Magalhães AR, et.al. [12] | Natural focal disease in global scale | Model: MaxEnt | The most significant predictive variables for relative variable importance = 37 ± 13% standard error. |
| Human Footprint | Gallardo B, et.al. [13] | The global distribution of invaders | Model: MaxEnt | Factors related to the human footprint explained a substantial amount (23% on average) of species distributions. |
|  | Skinner EB, et.al. [14] | The vector-borne diseases | Model：RF | Human footprint is an important predictor of local occurrence and that its nonlinear effects vary predictably with the transmission ecology of each vector-borne diseases |
| Rodentia richness | Usman S, et.al. [15] | Transmission dynamic of MPXV | Model: SEIR | Used in the monkeypox dynamic model. |
| Mammalian richness | Olival KJ, et.al. [16] | Zoonotic spillover from mammals | Model: GAM | Used in the spillover model. |

**Supplementary references**

1. Sage KM, Johnson TL, Schwan TG, et al. Ecological niche modeling and distribution of *Ornithodoros hermsi* associated with tick-borne relapsing fever in western North America. *PLoS Negl Trop Dis* 2017; **11:** e0006047.
2. Alkishe AA, Peterson AT, Samy AM. Climate change influences on the potential geographic distribution of the disease vector tick *Ixodes ricinus*. *PLoS One* 2017; **12:** e0189092.
3. Zhang L, Ma D, Li C, Zhou R, Wang J, Liu Q. Projecting the potential distribution areas of *Ixodes scapularis* (Acari: Ixodidae) driven by climate change. *Biology* 2022; **11:**107.
4. Eisen RJ, Feirer S, Padgett KA, et al. Modeling climate suitability of the western blacklegged tick in California. *J Med Entomol* 2018; **55:** 1133‒42.
5. Wang SS, Liu JY, Wang BY, et al. Geographical distribution of *Ixodes persulcatus* and associated pathogens: Analysis of integrated data from a China field survey and global published data. *One Health* 2023; **16:** 100508.
6. Fuller T, Thomassen HA, Mulembakani PM, et al. Using remote sensing to map the risk of human monkeypox virus in the Congo Basin. *Ecohealth* 2011; **8:** 14‒25.
7. Zhang YY, Liu W, Fang LQ, et al. Mapping the global distribution of spotted fever group rickettsiae: a systematic review with modelling analysis. *Lancet Digit Health* 2023; **5:** e5‒15.
8. Miao D, Liu W, Fang LQ, et al. Mapping the global potential transmission hotspots for severe fever with thrombocytopenia syndrome by machine learning methods. *Emerg Microbes Infect* 2020; **9:** 817‒26.
9. Allen T, Murray KA, Zambrana-Torrelio C, et al. Global hotspots and correlates of emerging zoonotic diseases. *Nat Commun* 2017; **8:** 1124.
10. Jones KE, Patel NG, Levy MA, et al. Global trends in emerging infectious diseases. *Microbes Infect* 2008; **451:** 990‒93.
11. Miao D, Liu MJ, Wang YX, et al. Epidemiology and ecology of severe fever with thrombocytopenia syndrome in China, 2010‒2018. *Clin Infect Dis* 2021; **73:** e3851‒58.
12. Magalhães AR, Codeço CT, Svenning JC, et al. Neglected tropical diseases risk correlates with poverty and early ecosystem destruction. *Infect Dis Poverty* 2023; **12:** 32.
13. Gallardo B, Zieritz A, Aldridge DC. The importance of the human footprint in shaping the global distribution of terrestrial, freshwater and marine invaders. *PLoS One* 2015; **10:** e0125801.
14. Skinner EB, Glidden CK, MacDonald AJ, Mordecai EA. Human footprint is associated with shifts in the assemblages of major vector-borne diseases. *Nat Sustain* 2023; **6:** 652‒61.
15. Sulaiman Usman, Ibrahim Isa Adamu. Modeling the transmission dynamics of the monkeypox virus infection with treatment and vaccination interventions. *J App Math Phys* 2017; **5:** 2335.
16. Olival KJ, Hosseini PR, Zambrana-Torrelio C, Ross N, Bogich TL, Daszak P. Host and viral traits predict zoonotic spillover from mammals. *Nature* 2017; **546:** 646‒50.

# Supplementary table 7: Variables used for ecological modelling in this study.

| **Classification of variables** | **Variable** | **Description** |
| --- | --- | --- |
| **Ecoclimatic variables** |  |  |
|  | BIO1 | Annual mean temperature (℃) |
|  | BIO2 | Mean diurnal range (Mean of monthly (max temp-min temp)) (℃) |
|  | BIO3 | Isothermality (BIO2/ BIO7) (×100) |
|  | BIO4 | Temperature seasonality (standard deviation×100) |
|  | BIO5 | Max temperature of warmest month (℃) |
|  | BIO6 | Min temperature of coldest month (℃) |
|  | BIO7 | Annual range of temperature (BIO5- BIO6) (℃) |
|  | BIO8 | Mean temperature of wettest quarter (℃) |
|  | BIO9 | Mean temperature of driest quarter (℃) |
|  | BIO10 | Mean temperature of warmest quarter (℃) |
|  | BIO11 | Mean temperature of coldest quarter (℃) |
|  | BIO12 | Annual precipitation (mm) |
|  | BIO13 | Precipitation of wettest month (mm) |
|  | BIO14 | Precipitation of driest month (mm) |
|  | BIO15 | Precipitation seasonality (Coefficient of variation) |
|  | BIO16 | Precipitation of wettest quarter (mm) |
|  | BIO17 | Precipitation of driest quarter (mm) |
|  | BIO18 | Precipitation of warmest quarter (mm) |
|  | BIO19 | Precipitation of coldest quarter (mm) |
| **Environmental variables** |  |  |
|  | Elevation | Average elevation (m) |
|  | Leaf area index | area of leaves (m²) over a unit of land (m²) |
|  | Cropland | Percentage coverage of cropland (%) |
|  | Mixed cropland and nature vegetation | Percentage coverage of mixed cropland and nature vegetation (%) |
|  | Forest | Percentage coverage of forest (%) |
|  | Shrubland | Percentage coverage of shrubland (%) |
|  | Mixed tree, shrub and herbaceous | Percentage coverage of mixed tree, shrub and herbaceous (%) |
|  | Grassland | Percentage coverage of grassland (%) |
|  | Lichens and mosses | Percentage coverage of lichens and mosses (%) |
|  | Sparse vegetation land | Percentage coverage of sparse vegetation land (%) |
|  | Flooded vegetation | Percentage coverage of flooded vegetation (%) |
|  | Urban built-up land | Percentage coverage of urban construction land (%) |
|  | Bare areas | Percentage coverage of bare areas (%) |
|  | Water body | Percentage coverage of inland water body (%) |
|  | Ice and snow | Percentage coverage of ice and snow (%) |
| **Biological variables** |  |  |
|  | Buffalo | Density of buffalo (heads per km²) |
|  | Cattle | Density of cattle (heads per km²) |
|  | Goat | Density of goat (heads per km²) |
|  | Sheep | Density of sheep (heads per km²) |
|  | Horse | Density of horse (heads per km²) |
|  | Mammalian richness* | The number of mammal species per km² |
|  | Rodent richness | The number of rodent species per km² |
| **Socioeconomic variables** |  |  |
|  | Population number | Average of population counts per km² |
|  | Global downscaled GDP | The assessment of GDP per raster |
|  | Human Footprint^#^ | The annual dynamics of the global human footprint per km² |

* These datasets were extracted from the NASA Socioeconomic Data and Applications Center (SEDAC) Gridded Species Distribution collection created from vector data files acquired from the International Union for Conservation of Nature (IUCN) Red List collection. The data represent the species of mammals at one kilometer resolution.

^#^ The dataset was extracted from the studies published on Scientific Data. The annual dynamics of the global human footprint from 2000 to 2018 were mapped with the Mollweide equal-area projection at 1km resolution, using eight variables including built environments, population density, nighttime light, croplands, pasture lands, roadways, railways, and navigable waterways as inputs, which reflect different aspects of human pressures. GDP, gross domestic product.

# Supplementary table 8: The 16 RFGB species with reports of human infection.

| **RFGB species** | **Number of reports/papers** | **Number of occurrence locations** | **Number of occurrence locations after 1980** |
| --- | --- | --- | --- |
| *Borrelia miyamotoi** | 223 | 704 | 704 |
| *Borrelia recurrentis* | 68 | 54 | 10 |
| *Borrelia hermsii** | 50 | 102 | 78 |
| *Borrelia lonestari** | 40 | 135 | 135 |
| *Borrelia crocidurae** | 38 | 176 | 164 |
| *Borrelia persica* | 31 | 65 | 34 |
| *Borrelia turicatae* | 26 | 51 | 33 |
| *Borrelia duttonii* | 18 | 24 | 18 |
| *Borrelia hispanica* | 14 | 25 | 20 |
| *Borrelia johnsonii* | 4 | 4 | 4 |
| *Borrelia venezuelensis* | 3 | 20 | 1 |
| *Candidatus* Borrelia fainii | 3 | 4 | 4 |
| *Candidatus* Borrelia kalaharica | 3 | 3 | 3 |
| *Borrelia microti* | 2 | 3 | 3 |
| *Borrelia caucasica* | 1 | 2 | 2 |
| *Candidatus* Borrelia algerica | 1 | 1 | 1 |

* Four major species of RFGB were included in the niche model based on the number of publications and occurrence locations in the database. RFGB, relapsing fever group *Borrelia*.

# Supplementary table 9: Detection of the four major RFGB species in ticks.

| **Species** | ***Borrelia lonestari* in ticks (N, %)** | ***Borrelia miyamotoi* in ticks (N, %)** | ***Borrelia hermsii* in ticks (N, %)** | ***Borrelia crocidurae* in ticks (N, %)** |
| --- | --- | --- | --- | --- |
| *Ornithodoros hermsi** |  |  | 37 (100) |  |
| *Ornithodoros sonrai** |  |  |  | 691 (86·48) |
| *Ornithodoros erraticus*^§^ |  |  |  | 103 (12·89) |
| *Ornithodoros marocanus* |  |  |  | 5 (0·63) |
| *Ixodes scapularis** | 57 (7·97) | 2488 (42·65) |  |  |
| *Ixodes ricinus** |  | 1320 (22·63) |  |  |
| *Ixodes persulcatus** |  | 1205 (20·66) |  |  |
| *Ixodes pacificus** |  | 590 (10·11) |  |  |
| *Amblyomma americanum** | 492 (68·81) | 28 (0·48) |  |  |
| *Rhipicephalus sanguineus* | 59 (8·25) |  |  |  |
| *Amblyomma maculatum* | 37 (5·17) | 1 (0·02) |  |  |
| *Haemaphysalis bandicota* | 27 (3·78) |  |  |  |
| *Dermacentor variabilis* | 18 (2·52) | 25 (0·43) |  |  |
| *Rhipicephalus microplus* | 9 (1·26) |  |  |  |
| *Haemaphysalis nepalensis* | 6 (0·84) |  |  |  |
| *Dermacentor albipictus* | 4 (0·56) |  |  |  |
| *Carios capensis* | 2 (0·28) |  |  |  |
| *Ixodes ovatus* | 2 (0·28) | 11 (0·19) |  |  |
| *Amblyomma testudinariuml* | 2 (0·28) | 7 (0·12) |  |  |
| *Ixodes pavlovskyi* |  | 42 (0·72) |  |  |
| *Haemaphysalis longicornis* |  | 30 (0·51) |  |  |
| *Ixodes hexagonus* |  | 20 (0·34) |  |  |
| *Dermacentor reticulatus* |  | 17 (0·29) |  |  |
| *Ixodes dentatus* |  | 15 (0·26) |  |  |
| *Ixodes granulatus* |  | 14 (0·24) |  |  |
| *Rhipicephalus microplus* |  | 7 (0·12) |  |  |
| *Ixodes nipponensis* |  | 4 (0·07) |  |  |
| *Ixodes frontalis* |  | 3 (0·06) |  |  |
| *Haemaphysalis japonica* |  | 2 (0·04) |  |  |
| *Dermacentor nuttalli* |  | 1 (0·02) |  |  |
| *Dermacentor silvarum* |  | 1 (0·02) |  |  |
| *Haemaphysalis concinna* |  | 1 (0·02) |  |  |
| *Haemaphysalis inermis* |  | 1 (0·02) |  |  |

*Seven tick species were determined as the main vectors of the four RFGB species based on the number and proportion of detecting with RFGB in ticks, which were included in the niche model. N represented the number of ticks with this RFGB detected in each tick species. RFGB, relapsing fever group *Borrelia*.

# Supplementary table 10: The number of studies and occurrence locations for the seven tick species.

| **Tick species** | **Number of studies collected** | **Number of studies included** | **Number of locations collected** | **Number of locations used in modelling analysis** |
| --- | --- | --- | --- | --- |
| *Ornithodoros hermsi* | 58 | 8 | 69 | 54 |
| *Ornithodoros sonrai* | 49 | 8 | 329 | 262 |
| *Ixodes scapularis* | 11643 | 292 | 5383 | 2326 |
| *Ixodes ricinus* | 12050 | 197 | 6130 | 4775 |
| *Ixodes persulcatus* | 9688 | 155 | 1439 | 726 |
| *Ixodes pacificus* | 793 | 129 | 1619 | 482 |
| *Amblyomma americanum* | 4765 | 106 | 3438 | 2087 |

| Proportion of sampling | 3:1* | |  | 4:1* | |  | 5:1* | |  | 3:1^#^ | |
| --- | --- | --- | --- | --- | --- | --- | --- | --- | --- | --- | --- |
|  | **Kappa (95% CI)** | **F1 Score (95% CI)** |  | **Kappa (95% CI)** | **F1 Score (95% CI)** |  | **Kappa (95% CI)** | **F1 Score (95% CI)** |  | **Kappa (95% CI)** | **F1 Score (95% CI)** |
| *Ixodes persulcatus* | 0·845(0·833‒0·858) | 0·885(0·849‒0·994) |  | 0·800(0·788‒0·813) | 0·823(0·801‒0·988) |  | 0·733(0·709‒0·801) | 0·762(0·721‒0·979) |  | 0·866(0·854‒0·877) | 0·901(0·861‒0·996) |
| *Ixodes ricinus* | 0·931 (0·928‒0·947) | 0·967 (0·941‒0·999) |  | 0·901 (0·885‒0·931) | 0·933 (0·912‒0·976) |  | 0·873 (0·828‒0·907) | 0·900 (0·893‒0·951) |  | 0·917 (0·908‒0·943) | 0·963 (0·933‒0·999) |
| *Ixodes scapularis* | 0·915 (0·908‒0·954) | 0·961 (0·930‒0·999) |  | 0·890 (0·857‒0·915) | 0·926 (0·911‒0·971) |  | 0·854 (0·818‒0·893) | 0·899 (0·883‒0·925) |  | 0·908 (0·878‒0·935) | 0·955 (0·903‒0·999) |
| *Ixodes pacificus* | 0·928 (0·919‒0·938) | 0·947 (0·926‒0·999) |  | 0·897 (0·919‒0·938) | 0·906 (0·886‒0·907) |  | 0·859 (0·816‒0·909) | 0·877 (0·809‒0·913) |  | 0·946 (0·938‒0·955) | 0·960 (0·937‒0·999) |
| *Amblyomma americanum* | 0·913 (0·904‒0·920) | 0·942 (0·921‒0·999) |  | 0·880 (0·897‒0·911) | 0·896 (0·855‒0·918) |  | 0·833 (0·801‒0·892) | 0·863 (0·803‒0·916) |  | 0·920 (0·917‒0·922) | 0·960 (0·938‒0·999) |
| *Ornithodoros hermsi* | 0·460(0·400‒0·521) | 0·499(0·269‒0·931) |  | 0·376(0·320‒0·432) | 0·400(0·241‒0·913) |  | 0·286(0·238‒0·334) | 0·305(0·222‒0·894) |  | 0·759(0·692‒0·826) | 0·826(0·212‒0·988) |
| *Ornithodoros sonrai* | 0·784(0·755‒0·814) | 0·844(0·822‒0·990) |  | 0·792(0·766‒0·818) | 0·820(0·808‒0·988) |  | 0·734(0·707‒0·761) | 0·760(0·774‒0·979) |  | 0·844(0·819‒0·868) | 0·879(0·846‒0·994) |

# Supplementary table 11: Kappa coefficient and F1 score of the BRT models obtained for each tick species with different sampling methods and ratios.

*The “control” grids were randomly sampled from the background grids at different ratios. ^#^ The “control” grids were randomly sampled from the grids within the study area at 3:1.

# Supplementary table 12: Vectors with record of RFGB detecting and evidence of biting human.

| Vector | Species | Reference |
| --- | --- | --- |
| Tick | *Amblyomma americanum* | Madison‒Antenucci S, Kramer LD, Gebhardt LL, Kauffman E. Emerging Tick-Borne Diseases. *Clin Microbiol Rev* 2020; **33:** e00083‒18. |
|  | *Amblyomma variegatum* | Petney TN, Horak IG, Rechav Y. The ecology of the African vectors of heartwater, with particular reference to *Amblyomma hebraeum* and *Amblyomma variegatum*. Onderstepoort. *J Vet Res* 1987; **54:** 381‒95. |
|  | *Amblyomma maculatum* | Zemtsova GE, Watkins NE, JRhipicephalus, Levin ML. Multiplex qPCR assay for identification and differentiation of *Amblyomma americanum*, *Amblyomma cajennense*, and *Amblyomma maculatum* (Ixodida: Ixodidae) tick species in the eastern United States. *J Med Entomol* 2014; **51:** 795‒803. |
|  | *Amblyomma testudinarium* | Chao LL, Lu CW, Lin YF, Shih CM. Molecular and morphological identification of a human biting tick, *Amblyomma testudinarium* (Acari: Ixodidae), in Taiwan. *Exp Appl Acarol* 2017; **71:** 401‒14. |
|  | *Dermacentor variabilis* | Madison‒Antenucci S, Kramer LD, Gebhardt LL, Kauffman E. Emerging Tick-borne diseases. *Clin Microbiol Rev* 2020; **33:** e00083‒18. |
|  | *Dermacentor nuttalli* | Khasnatinov MA, Liapunov AV, Manzarova EL, Kulakova NV, Petrova IV, Danchinova GA. The diversity and prevalence of hard ticks attacking human hosts in Eastern Siberia (Russian Federation) with first description of invasion of non-endemic tick species. *Parasitol Res* 2016; **115:** 501‒10. |
|  | *Dermacentor reticulatus* | Khasnatinov MA, Liapunov AV, Manzarova EL, Kulakova NV, Petrova IV, Danchinova GA. The diversity and prevalence of hard ticks attacking human hosts in Eastern Siberia (Russian Federation) with first description of invasion of non-endemic tick species. *Parasitol Res* 2016; **115:** 501‒10. |
|  | *Dermacentor silvarum* | Guo WB, Shi WQ, Wang Q, et al. Distribution of *Dermacentor silvarum* and Associated Pathogens: Meta-Analysis of Global Published Data and a Field Survey in China. *Int J Environ Res Public Health* 2021; **18:** 4430. |
|  | *Haemaphysalis longicornis* | Madison‒Antenucci S, Kramer LD, Gebhardt LL, Kauffman E. Emerging tick-borne diseases. *Clin Microbiol Rev* 2020; **33:** e00083‒18. |
|  | *Haemaphysalis concinna* | Khasnatinov MA, Liapunov AV, Manzarova EL, Kulakova NV, Petrova IV, Danchinova GA. The diversity and prevalence of hard ticks attacking human hosts in Eastern Siberia (Russian Federation) with first description of invasion of non-endemic tick species. *Parasitol Res* 2016; **115:** 501‒10. |
|  | *Haemaphysalis flava* | Ozawa A, Yamaguchi N, Hayakawa K, Matsuo I, Niizuma K, Ohkido M. A case of tick bite (*Haemaphysalis flava*) consideration of tularemia infection through tick bite. *Nihon Hifuka Gakkai Zasshi* 1982; **92:** 1415‒21. |
|  | *Haemaphysalis nepalensis* | H HOOGSTRAAL. Haemaphysalis nepalensis sp. n. from a Himalayan rodent and man, and description of the male of *H. aponommoides* Warburton (n. comb.) (Ixodoidea, Ixodidae). *J Parasit* 1962; **48:** 195‒203. |
|  | *Haemaphysalis japonica* | Sasaki K, Honma M, Nakao M, et al. Survey to detect Tick-borne encephalitis virus from human-feeding ticks in Hokkaido, Japan. *J Dermatol* 2021; **48:** 1094‒97. |
|  | *Ixodes pacificus* | David T Gauthier, Christopher D Paddock, et al. Characterization of a novel transitional group Rickettsia species (*Rickettsia tillamookensis* sp. nov.) from the western black-legged tick, *Ixodes pacificus*. *Int J Syst Evol Microbiol* 2021; **71:** 004880. |
|  | *Ixodes persulcatus* | Madison‒Antenucci S, Kramer LD, Gebhardt LL, Kauffman E. Emerging tick-borne diseases. *Clin Microbiol Rev* 2020; **33:** e00083‒18. |
|  | *Ixodes ricinus* | Madison‒Antenucci S, Kramer LD, Gebhardt LL, Kauffman E. Emerging tick-borne diseases. *Clin Microbiol Rev* 2020; **33:** e00083‒18. |
|  | *Ixodes scapularis* | Madison‒Antenucci S, Kramer LD, Gebhardt LL, Kauffman E. Emerging tick-borne diseases. *Clin Microbiol Rev* 2020; **33:** e00083‒18. |
|  | *Ixodes ovatus* | Sasaki K, Honma M, Nakao M, et al. Survey to detect tick-borne encephalitis virus from human-feeding ticks in Hokkaido, Japan. *J Dermatol* 2021; **48:** 1094‒97. |
|  | *Ixodes frontalis* | Gilot B, Beaucournu JC, Chastel C. Collecting with the flagging method and fixing on man of *Ixodes* (Trichotoixodes) *frontalis* (Panzer, 1795). *Parasite* 1997; **4:** 197‒99. |
|  | *Ixodes hexagonus* | Faulde MK, Rutenfranz M, Hepke J, Rogge M, Görner A, Keth A. Human tick infestation pattern, tick-bite rate, and associated Borrelia burgdorferi s.l. infection risk during occupational tick exposure at the Seedorf military training area, northwestern Germany. *Ticks Tick Borne Dis* 2014; **5:** 594‒99. |
|  | *Ixodes nipponensis* | Lee SH, Shin NR, Kim CM, et al. First identification of *Anaplasma phagocytophilum* in both a biting tick *Ixodes nipponensis* and a patient in Korea: a case report. *BMC Infect Dis* 2020; **20:** 826. |
|  | *Ixodes dentatus* | J F Anderson, D Mathiesen, E Fikrig, et al. Novel *Borrelia burgdorferi* isolates from *Ixodes scapularis* and *Ixodes dentatus* ticks feeding on humans. *J Clin Microbiol* 1996; **34:** 524‒29. |
|  | *Rhipicephalus sanguineus* | Madison‒Antenucci S, Kramer LD, Gebhardt LL, Kauffman E. Emerging ttick-borne diseases. *Clin Microbiol Rev* 2020; **33:** e00083‒18. |
|  | *Rhipicephalus microplus* | Szabó MPJ, Martins TF, Barbieri ARM, et al. Ticks biting humans in the Brazilian savannah: attachment sites and exposure risk in relation to species, life stage and season. *Ticks Tick Borne Dis* 2020; **11:** 101328. |
|  | *Rhipicephalus annulatus* | Keskin A, Keskin A, Bursali A, Tekin S. Ticks (Acari: Ixodida) parasitizing humans in Corum and Yozgat provinces, Turkey. *Exp Appl Acarol* 2015; **67:** 607‒16. |
|  | *Rhipicephalus bursa* | Keskin A, Keskin A, Bursali A, Tekin S. Ticks (Acari: Ixodida) parasitizing humans in Corum and Yozgat provinces, Turkey. *Exp Appl Acarol* 2015; **67:** 607‒16. |
|  | *Argas miniatus* | Dehhaghi M, Kazemi Shariat Panahi H, Holmes EC, Hudson BJ, Schloeffel R, Guillemin GJ. Human tick-borne diseases in Australia. *Front Cell Infect Microbiol* 2019; **9:** 3. |
|  | *Argas persicus* | Dehhaghi M, Kazemi Shariat Panahi H, Holmes EC, Hudson BJ, Schloeffel R, Guillemin GJ. Human tick-borne diseases in Australia. *Front Cell Infect Microbiol* 2019; **9:** 3. |
|  | *Carios kelleyi* | Nadolny RM, Kennedy AC, Farris CM, et al. *Carios kelleyi* (Acari: Ixodida: Argasidae) Infected with Rickettsial Agents Documented Infesting Housing in Kansas, United States. *J Med Entomol*v 2021; **58:** 2398‒405. |
|  | *Hyalomma aegyptium* | Kar S, Rodriguez SE, Akyildiz G, Cajimat MNB, Bircan R, Mears MC, Bente DA, Keles AG. Crimean-Congo hemorrhagic fever virus in tortoises and *Hyalomma aegyptium* ticks in East Thrace, Turkey: potential of a cryptic transmission cycle. *Parasit Vectors* 2020; **13:** 201. |
|  | *Ornithodoros verrucosus* | Filatov S, Krishnavajhala A, Armstrong BA, et al. Isolation and Molecular Characterization of Tick-borne relapsing fever *Borrelia* Infecting *Ornithodoros (Pavlovskyella) verrucosus* ticks collected in Ukraine. *J Infect Dis* 2020; **221:** 804‒11. |
|  | *Ornithodoros erraticus* | Palma M, Lopes de Carvalho I, Figueiredo M, et al. *Borrelia hispanica* in *Ornithodoros erraticus*, Portugal. *Clin Microbiol Rev* 2012; **18:** 696‒701. |
|  | *Ornithodoros faini* | Yongjin Qiu, Ryo Nakao, Hiroki Kawabata. Human borreliosis caused by a New World relapsing fever *Borrelia*-like organism in the Old World. *Clin Infect Dis* 2019; 69: 107‒12. |
|  | *Ornithodoros hermsi* | Mark S Dworkin, Tom G Schwan, Stephanie M Borchardt. Tick-borne relapsing fever. *Infect Dis Clin North Am* 2008; **22:** 449‒68. |
|  | *Ornithodoros* *mimon* | Labruna MB, Marcili A, Venzal JM, et al. New records and human parasitism by *Ornithodoros mimon* (Acari: Argasidae) in Brazil. *J Med Entomol* 2014; **51:** 283‒87. |
|  | *Ornithodoros moubata* | Verónica Díaz‒Martín, Raúl Manzano‒Román, et al. Development of vaccines against *Ornithodoros* soft ticks: an update. *Ticks Tick Borne Dis* 2015; **6:** 211‒20. |
|  | *Ornithodoros parkeri* | Mark S Dworkin, Tom G Schwan, Stephanie M Borchardt. Tick-borne relapsing fever. *Infect Dis Clin North Am* 2008; **22:** 449‒68. |
|  | *Ornithodoros puertoricensis* | Bermúdez SE, Castillo E, López JE, et al. New records of *Ornithodoros puertoricensis* Fox 1947 (Ixodida: Argasidae) parasitizing humans in rural and urban dwellings, Panama. *Ticks Tick Borne Dis* 2017; **8:** 466‒69. |
|  | *Ornithodoros rietcorreai* | Bitencourth K, Borsoi ABP, Gazeta GS, et al. Human parasitism and toxicosis by *Ornithodoros rietcorreai* (Acari: Argasidae) in an urban area of Northeastern Brazil. *Ticks Tick Borne Dis* 2018; **9:** 1494‒98. |
|  | *Ornithodoros turicata* | Mark S Dworkin, Tom G Schwan, Stephanie M Borchardt. Tick-borne relapsing fever. *Infect Dis Clin North Am* 2008; **22:** 449‒68. |
|  | *Ornithodoros savignyi* | Sally J Cutler, Jibrin M Idris, Akeem O Ahmed, Nusirat Elelu. *Ornithodoros savignyi*, the tick vector of "*Candidatus* Borrelia kalaharica" in Nigeria. *J Clin Microbiol* 2018; **56:** e00532‒18. |
|  | *Ornithodoros sonrai* | Laurence Vial, Georges Diatta, Jean‒François Trape. Incidence of tick-borne relapsing fever in west Africa: longitudinal study. *Lancet* 2006; **368:** 37‒43. |
|  | *Ornithodoros tholozani* | B BABUDIERI. Relapsing fever in Jordan. *Bull World Health Organ* 1957; **16:** 911‒28. |
| Louse | *Pediculus humanus capitis* | Coates SJ, Thomas C, Chosidow O, Engelman D, Chang AY. Ectoparasites: Pediculosis and tungiasis. *J Am Acad Dermatol* 2020; **82:** 551‒69. |
|  | *Pediculus humanus corporis* | Yassina Bechah, Christian Capo, Jean‒Louis Mege, Didier Raoult. Epidemic typhus. *Lancet Infect Dis* 2008; **8:** 417‒26. |

# Supplementary table 13: The coinfection of *Borrelia miyamotoi* with Lyme disease spirochete in ticks.

| **Relapsing fever group *Borrelia*** | ***Borrelia burgdorferi* sensu lato** | **Detected vectors** |
| --- | --- | --- |
| *Borrelia miyamotoi* | *Borrelia afzelii* | *Ixodes ricinus, Ixodes persulcatus, Ixodes pacificus* |
| *Borrelia miyamotoi* | *Borrelia bavariensis* | *Ixodes ricinus* |
| *Borrelia miyamotoi* | *Borrelia burgdorferi* | *Ixodes ricinus, Ixodes persulcatus, Ixodes pacificus, Ixodes scapularis, Haemaphysalis longicornis* |
| *Borrelia miyamotoi* | *Borrelia garinii* | *Ixodes ricinus, Ixodes persulcatus* |
| *Borrelia miyamotoi* | *Borrelia valaisiana* | *Ixodes ricinus, Ixodes persulcatus* |

# Supplementary table 14: The number of human cases with RFGB infection by diagnosis methods.

| **Classification** | | **RFGB species** | **No. human cases** | **Diagnosis methods** | | | **Reported clinical manifestations**^§^ **(n, %)** |
| --- | --- | --- | --- | --- | --- | --- | --- |
|  |  |  |  | **Molecular assays**  **(n, %)*** | **Morphology identification (n, %)*** | **Serological assay (n, %)*** |  |
|  | | Overall | 21 876 | 2 042 (9·3) | 19 610 (89·7) | 224 (1·0) | 12 445 (100) ^§^ |
| **LBRF** | *Borrelia recurrentis* | | 17 084 | 81 (0·5) | 17 001 (99·5) | 2 (~0·0) | 8 974 (72·1) |
| **TBRF** | Subtotal | | 4 792 | 1 961 (41·0) | 2 609 (54·4) | 222 (4·6) | 3 471 (37·9) |
|  | *Borrelia persica* | | 2 045 | 128 (6.3) | 1 917 (93·7) |  | 2 045 (16·5) |
|  | *Borrelia crocidurae* | | 894 | 851 (95·2) | 38 (4·2) | 5 (0·6) | 414 (3·3) |
|  | *Borrelia miyamotoi* | | 825 | 820 (99·4) |  | 5 (0·6) | 374 (3·0) |
|  | *Borrelia hermsii* | | 458 | 40 (8·7) | 218 (47·6) | 200 (43·7) | 339 (2·7) |
|  | *Borrelia duttonii* | | 177 | 53 (29·9) | 124 (70·1) |  | 147 (1·2) |
|  | *Borrelia hispanica* | | 150 | 54 (36·0) | 87 (58·0) | 9 (6·0) | 137 (1·1) |
|  | *Borrelia venezuelensis* | | 223 |  | 223 (100) |  |  |
|  | *Borrelia turicatae* | | 9 | 4 (44·4) | 2 (22·2) | 3 (33·3) | 9 (0·1) |
|  | *Candidatus* Borrelia kalaharica | | 2 | 2 (100·0) |  |  | 2 (~0·0) |
|  | *Borrelia caucasica* | | 1 | 1 (100·0) |  |  | 1 (~0·0) |
|  | *Borrelia lonestari* | | 1 | 1 (100·0) |  |  | 1 (~0·0) |
|  | *Borrelia microti* | | 1 | 1 (100·0) |  |  | 1 (~0·0) |
|  | *Candidatus* Borrelia fainii | | 1 | 1 (100·0) |  |  | 1 (~0·0) |
|  | *Candidatus* Borrelia algerica | | 4 | 4 (100·0) |  |  |  |
|  | *Candidatus* Borrelia johnsonii | | 1 | 1 (100·0) |  |  |  |

Only 21876 human cases with determined RFGB species were counted, and 4707 human cases with no determined RFGB species were not shown in table.

*Data are presented as the numbers and proportions (%) of cases that were diagnosed by each method.

^§^ Data are presented as the number and proportion (%) cases that reported clinical manifestation among all cases.

Serological assay refers to the cases confirmed by IgG positive serum sample in convalescence.

RFGB, relapsing fever group *Borrelia*. LBRF, louse-borne relapsing fever. TBRF, tick-borne relapsing fever.

Supplementary table 15: Clinical characteristics of human infections with RFGB.

| **Symptoms** | **TBRF (No. of reported cases)** | | | | | |  | **LBRF (No. of reported cases)** |
| --- | --- | --- | --- | --- | --- | --- | --- | --- |
|  | ***Borrelia persica***  **(n=2045)** | ***Borrelia*** ***crocidurae***  **(n=414)** | ***Borrelia miyamotoi***  **(n=374)** | ***Borrelia hermsii***  **(n=339)** | ***Borrelia duttonii***  **(n=147)** | ***Borrelia hispanica***  **(n=137)** |  | ***Borrelia recurrentis***  **(n=8974)** |
| **Influenza-like symptom** | | | | | | | | |
| Fever | 2 000 (97·8) | 397 (95·9) | 313 (83·7) | 261‒267 (77·0‒78·8) | 136‒147 (92·5‒100·0) | 134 (97·8) |  | 6827 (76·1) |
| Headaches | 198 (9·7) | **389 (94·0)** | **276 (73·8)** | **240‒241 (70·8‒71·1)** | 50‒52 (34·0‒35·4) | 65‒67 (47·4‒48·9) |  | 1334‒1367 (14·9‒15·2) |
| Chills | 277 (13·5) | 10‒13 (2·4‒3·1) | **221‒224 (59·1‒60·0)** | **184‒189 (54·3‒55·8)** | 16‒20 (10·9‒13·6) | 49 (35·8) |  | 582‒642 (6·5‒7·2) |
| Myalgia | 105‒108 (5·1‒5·3) |  | **187 (50·0)** | 221 (65·2) | 18‒20 (12·2‒13·6) | 50 (36·5) |  | 551‒572 (6·1‒6·4) |
| Cough | 43‒47 (2·1‒2·3) |  |  | 21‒28 (6·2‒8·3) |  |  |  | 226‒298 (2·5‒3·3) |
| Weakness | 76‒80 (3·7‒3·9) | **334 (80·7)** | 95‒103 (25·4‒27·5) | 65‒76 (19·2‒22·4) |  |  |  | 56‒150 (0·6‒1·7) |
| Sweats | 144 (7·0) |  | 143‒151 (38·2‒40·4) | 70‒79 (20·6‒23·3) |  |  |  |  |
| Fatigue |  |  | 44‒54 (11·8‒14·4) | 20‒30 (5·9‒8·8) |  |  |  |  |
| **Gastrointestinal symptoms** | | | | | | | | |
| Vomit | 59‒63 (2·9‒3·1) | 66 (15·9) | 65‒74 (17·4‒20·0) | **107‒111 (31·6‒32·7)** |  | **57 (41·6)** |  | 493‒543 (5·5‒6·1) |
| Abdominal pain | 93‒97 (4·5‒4·7) |  | 10‒21 (2·7‒5·6) | 64‒72 (18·9‒21·2) |  | 37‒39 (27·0‒28·5) |  | 435‒514 (4·8‒5·7) |
| Anorexia |  |  |  | 53‒65 (15·6‒19·2) |  |  |  | 303‒378 (3·4‒4·2) |
| Diarrhea |  |  |  | 27‒35 (8·0‒10·3) | **59‒62 (40·1‒42·2)** | 21 (15·3) |  | 133‒202 (1·5‒2·3) |
| Nausea | 71‒75 (3·5‒3·7) |  | 33‒43 (8·8‒11·5) | **104‒115 (30·7‒33·9)** |  |  |  |  |
| Constipation |  |  |  |  | 18‒21 (12·2‒14·3) |  |  | 161‒249 (1·8‒2·8) |
| **Neurological symptoms** | | | | | | | | |
| Malaise | 36‒42 (1·8‒2·1) |  | 45‒56 (12·0‒15·0) |  |  |  |  | 170‒248 (1·9‒2·8) |
| Neck stiffness |  |  |  | 37‒48 (10·9‒14·2) |  |  |  | 42‒133 (0·5‒1·5) |
| Febrile  convulsion |  |  |  |  | **22‒25 (15·0‒17·0)** |  |  |  |
| **Hematologic disorder** | | | | | | | | |
| Hepatosplenomegaly |  |  |  |  |  |  |  | 634‒711 (7·1‒7·9) |
| Epistaxis | 17 (0·8) |  |  |  | 13‒16 (8·8‒10·9) |  |  | 391‒443 (4·4‒4·9) |
| Jaundice | 25‒32 (1·2‒1·6) |  | 41‒52 (11·0‒13·9) |  |  |  |  | 543‒621 (6·1‒6·9) |
| Thrombocyto-penia | 50 (2·4) |  | 41‒50 (11·0‒13·4) | 76‒85 (22·4‒25·1) |  | **97‒99 (70·8‒72·3)** |  | 120‒200 (1·3‒2·2) |
| Anemia |  |  |  |  | 18‒21 (12·2‒14·3) | 34‒37 (24·8‒27·0) |  | 104‒194 (1·2‒2·2) |
| **Other symptoms** | | | | | | | | |
| Arthralgias | 44‒48 (2·2‒2·3) |  | 94‒101 (25·1‒27·0) | 93‒98 (27·4‒28·9) | 12‒16 (8·2‒10·9) | 30‒32 (21·9‒23·3) |  | 557‒598 (6·2‒6·7) |
| Rash | 13‒19 (0·6‒0·9) |  |  | 22‒31 (6·5‒9·1) |  |  |  | 271‒337 (3·0‒3·8) |
| Erythema migrans |  |  | **9‒22 (2·4‒5·9)** |  |  |  |  |  |
| Dehydration |  |  | 45‒56 (12·0‒15·0) |  |  |  |  | 35‒82 (0·4‒0·9) |
| Dyspnea |  |  | 23‒35 (6·1‒9·4) |  |  |  |  | 47‒138 (0·5‒1·5) |
| Eye ache |  |  |  | **43‒54 (12·7‒15·9)** |  |  |  |  |
| Red eyes |  |  |  | **29 (6·0)** |  |  |  |  |
| Conjunctivitis |  |  |  |  | **12‒21 (8·2‒14·3)** |  |  |  |

Data are presented as numbers of positive cases and proportions (%). Only RFGB species with ≥10 cases are shown. Symptoms of less than 10 cases in all RFGB species were not displayed. Erythema migrans was the symptom presented only in cases with *Borrelia miyamotoi* infection and was specially reserved.

When calculating the frequency of each clinical feature, we divided publications into two groups, case reports and case series. A case report describes clinical features of a single patient in detail, for which it is reasonable to assume unmentioned symptoms as absent. In contrast, a case series study summarizes clinical characteristics of a group of confirmed patients, for which it is unclear if unmentioned symptoms are truly absent from the whole group or just rare, especially when the group size is large. For case series studies, we therefore made a conservative assumption that the frequency of an unreported symptom could vary from 0 to the minimum frequency of all reported symptoms. Consequently, we report a range for each symptom if relevant data involve case series. The data of the symptoms reported by only one species of RFGB infection cases and predominant symptom were bolded. RFGB, relapsing fever group *Borrelia*. LBRF, louse-borne relapsing fever. TBRF, tick-borne relapsing fever.

# Supplementary table 16: The demographic features and the main clinical data of 226 case reports.

| **Pathogen types** | | **Gender (n=226)** | |  | **Mean age of patients (n=226)** | **Recurrent fever (n=153)** | **Thrombocy­topenia (n=89)** | **Anemia (n=26)** | **Taking Doxycycline (n=76)** | **JHR (n=38)** | **Death (n=7)** |
| --- | --- | --- | --- | --- | --- | --- | --- | --- | --- | --- | --- |
|  |  | **Male (n=86)** | **Female (n=140)** |  |  |  |  |  |  |  |  |
| *Borrelia crocidurae* | 11 | | 11 |  | 34·2 | 15 | 6 | 3 | 10 | 1 | 0 |
| *Borrelia duttonii* | 3 | | 5 |  | 23·5 | 3 | 0 | 0 | 1 | 2 | 2 |
| *Borrelia hermsii* | 16 | | 23 |  | 37·0 | 32 | 18 | 2 | 11 | 9 | 1 |
| *Borrelia hispanica* | 8 | | 5 |  | 10·9 | 12 | 7 | 2 | 1 | 2 | 0 |
| *Borrelia lonestari* | 0 | | 1 |  | 74 | 0 | 0 | 0 | 1 | 0 | 0 |
| *Borrelia microti* | 1 | | 0 |  | 33 | 1 | 1 | 1 | 0 | 0 | 0 |
| *Borrelia miyamotoi* | 12 | | 16 |  | 58·1 | 10 | 13 | 2 | 9 | 1 | 0 |
| *Borrelia persica* | 2 | | 12 |  | 21·6 | 11 | 3 | 0 | 4 | 5 | 0 |
| *Borrelia recurrentis* | 6 | | 27 |  | 24·9 | 19 | 23 | 12 | 14 | 10 | 1 |
| *Borrelia turicatae* | 1 | | 5 |  | 29·9 | 5 | 3 | 0 | 2 | 0 | 0 |
| *Candidatus* Borrelia fainii | 0 | | 1 |  | 35 | 1 | 0 | 0 | 0 | 0 | 0 |
| *Candidatus* Borrelia kalaharica | 2 | | 0 |  | 48·5 | 2 | 1 | 0 | 2 | 1 | 0 |
| Tick-borne *Borrelia* | 24 | | 34 |  | 26·4 | 38 | 14 | 4 | 21 | 7 | 3 |

Of the therapeutic agents, we show only the most frequently used doxycycline. Variables including lymphopenia and use of amoxicillin, piperacillin, minocycline, cefotaxime, ofloxacin, ampicillin, ciprofloxacin, chloramphenicol were not showed due to the low reporting proportions (less than 5%) in the cases.

# Supplementary table 17: The logistic regression analysis of JHR occurrence in relapsing fever cases.

| **Variables** | **Univariate analysis** | |  | **Multivariate analysis** | |
| --- | --- | --- | --- | --- | --- |
|  | **OR (95% CI)** | **P value** |  | **OR (95% CI)** | **P value** |
| Age | 0·999 (0·981‒1·015) | 0·865 |  |  |  |
| Gender | 0·769 (0·379‒1·586) | 0·469 |  |  |  |
| Thrombocytopenia | 2·401 (1·186‒4·956) | 0·016 |  | **2·437 (1·158‒5·255)** | **0·020** |
| Episodes of fever | 1·326 (0·961‒1·820) | 0·080 |  | **1·432(1·014‒2·025)** | **0·040** |
| Anemia | 1·961 (0·716‒4·890) | 0·164 |  |  |  |
| Medical usage |  |  |  |  |  |
| Ceftriaxone | 2·716 (1·163‒6·100) | 0·017 |  | 2·291(0·955‒5·283) | 0·056 |
| Doxycycline | 1·715 (0·835‒3·488) | 0·137 |  |  |  |
| Erythromycin | 0·595 (0·032‒3·384) | 0·629 |  |  |  |
| Penicillin | 1·325 (0·361‒3·920) | 0·635 |  |  |  |
| Tetracycline | 1·207 (0·453‒2·875) | 0·686 |  |  |  |

In the univariate analysis, the variables with p values less than 0·1 were included into the multivariate analysis, and those meeting the significant level (p <0·05) in the multivariate model were bolded. Variables including lymphopenia and use of amoxicillin, piperacillin, minocycline, cefotaxime, ofloxacin, ampicillin, ciprofloxacin, chloramphenicol were not included in the analysis due to the low reporting proportions (less than 5%) in the cases. JHR, Jarisch-Heyxheimer reaction. OR, odds ratio. CI, confidence interval.

# Supplementary table 18: The RCs of significant contributors to the occurrence of seven main species of ticks based on BRT models.

| **Variable** | ***I. persulcatus*** | ***I. ricinus*** | ***I. scapularis*** | ***I. pacificus*** | ***A. americanum*** | ***O. hermsi*** | ***O. sonrai*** |
| --- | --- | --- | --- | --- | --- | --- | --- |
| Annual mean temperature | 5·9 (5·3‒6·5) | 5·0 (4·7‒5·3) |  |  | 13·1 (7·6‒18·6) | 3·1 (1·5‒4·7) |  |
| Isothermality | 8·1 (7·3‒8·9) |  | 3·9 (3·2‒4·6) |  | 4·9 (4·2‒5·6) | 3·2 (1·5‒4·9) | 4·7 (3·7‒5·7) |
| Temperature seasonality | 5·1 (4·5‒5·7) | 12·2 (11·1‒13·3) |  |  |  |  |  |
| Max temperature of warmest month | 3·4 (2·7‒4·1) |  |  |  |  |  |  |
| Annual range of temperature |  | 12·9 (10·6‒15·2) |  |  |  |  |  |
| Mean temperature of wettest quarter |  |  |  | 3·2 (2·8‒3·6) |  | 6·4 (4·0‒8·8) |  |
| Mean temperature of driest quarter | 4·8 (4·3‒5·3) |  |  |  |  |  |  |
| Mean temperature of warmest quarter | 3·4 (3·0‒3·8) |  | 41·0 (38·7‒43·3) |  | 29·0 (23·1‒34·9) |  |  |
| Annual precipitation |  | 23·3 (21·0‒25·6) |  |  |  |  |  |
| Precipitation of wettest month |  |  | 3·9 (1·9‒5·9) | 3·4 (2·1‒4·7) | 29·7 (22·9‒36·5) |  |  |
| Precipitation of driest month |  |  | 16·0 (14·7‒17·3) |  |  |  |  |
| Precipitation seasonality |  |  |  |  |  |  | 24·4 (21·9‒26·9) |
| Precipitation of driest quarter |  |  |  |  | 8·5 (6·7‒10·3) |  |  |
| Precipitation of warmest quarter |  | 4·3 (3·4‒5·2) | 4·2 (3·0‒5·4) | 62·9 (61·6‒64·2) |  |  | 3·0 (2·4‒3·6) |
| Precipitation of coldest quarter |  |  |  | 16·1 (14·6‒17·6) |  | 4·3 (2·7‒5·9) |  |
| Forest |  | 3·8 (3·3‒4·3) |  |  |  |  |  |
| Urban built-up land | 7·2 (6·8‒7·6) |  | 12·6 (11·1‒14·1) |  |  |  |  |
| Leaf area index | 19·2 (18·3‒20·1) |  |  |  |  |  |  |
| Elevation |  |  |  |  |  |  | 15·8 (14·3‒17·3) |
| Buffalo | 16·8 (15·8‒17·8) |  |  |  |  |  |  |
| Goat | 3·8 (3·4‒4·2) | 3·4 (3·1‒3·7) |  |  |  |  |  |
| Horse |  | 7·2 (6·4‒8·0) |  |  |  |  | 5·0 (3·8‒6·2) |
| Sheep |  |  |  |  |  |  | 8·4 (6·6‒10·2) |
| Mammalian richness | 3·1 (2·8‒3·4) |  |  |  |  | 52·8 (46·0‒59·6) |  |
| Rodent richness | 3·2 (2·7‒3·7) |  |  |  |  | 14·0 (7·3‒20·7) |  |

# The mean (95% CI) RCs of each model were displayed. We only demonstrated effects with mean RC >3%. RC, relative contribution. BRT, boosted regression trees.

#

# Supplementary table 19: The RCs of significant contributors to the spatial distribution of the four major RFGB species based on RF models.

| **Variable** | ***Borrelia miyamotoi*** | ***Borrelia lonestari*** | ***Borrelia hermsii*** | ***Borrelia crocidurae*** |
| --- | --- | --- | --- | --- |
| Annual mean temperature | 3·2 (1·6‒4·8) | 6·8 (5·6‒8·0) | 3·5 (3·2‒3·8) |  |
| Isothermality |  |  | 4·2 (3·7‒4·7) |  |
| Temperature seasonality |  |  | 4·4 (3·9‒4·9) |  |
| Max temperature of warmest month |  | 3·2 (2·5‒3·9) |  |  |
| Min temperature of coldest month |  | 3·0 (2·3‒3·7) | 4·3 (3·8‒4·8) |  |
| Mean temperature of wettest quarter |  |  | 3·4 (3·0‒3·8) |  |
| Mean temperature of driest quarter |  |  | 4·9 (4·3‒5·5) |  |
| Mean temperature of warmest quarter |  | 9·2 (8·0‒10·4) |  |  |
| Mean temperature of coldest quarter |  |  | 4·4 (4·0‒4·8) |  |
| Annual precipitation |  | 3·5 (2·7‒4·3) |  |  |
| Precipitation of wettest month |  | 5·9 (4·8‒7·0) |  |  |
| Precipitation seasonality |  |  |  | 8·0 (6·1‒9·9) |
| Precipitation of wettest quarter |  | 6·7 (5·5‒7·9) |  |  |
| Precipitation of warmest quarter |  | 4·1 (3·1‒5·1) | 4·0 (3·5‒4·5) |  |
| Cropland | 3·4 (1·6‒5·2) |  |  |  |
| Urban built-up land | 9·9 (5·1‒14·7) |  |  |  |
| Elevation |  |  |  | 8·7 (6·8‒10·6) |
| Goat |  |  |  | 3·1 (2·2‒4·0) |
| Horse |  | 3·2 (2·3‒4·1) |  | 6·3 (4·6‒8·0) |
| Sheep |  |  |  | 3·7 (2·6‒4·8) |
| Mammalian richness |  |  | 8·1 (7·3‒8·9) |  |
| Rodent richness |  |  | 5·4 (4·8‒6·0) |  |
| Habitat suitability index of *O. hermsi* |  |  | 16·4 (15·3‒17·5) |  |
| Habitat suitability index of *O. sonrai* |  |  |  | 19·8 (16·7‒22·9) |
| Habitat suitability index of *A. americanum* |  | 17·0 (15·4‒18·6) |  |  |
| Habitat suitability index of ticks | 21·2 (13·8‒28·6) |  |  |  |
| Human Footprint | 6·1 (2·8‒9·4) |  |  | 3·2 (2·2‒4·2) |
| Population density | 6·0 (3·2‒8·8) | 3·3 (2·5‒4·1) |  |  |

# The mean (95% CI) RCs of each model were displayed. We only demonstrated effects with mean RC >3%. RC, relative contribution; RFGB, relapsing fever group *Borrelia*; RF, random forest.

# Supplementary table 20: Comparison of model performance for RFGB niche modelling of the four RFGB species by using different thresholds for polygon occurrences.

| RFGB species | Threshold | Accuracy | AUC | Sensitivity | Specificity | F1 Score | Kappa |
| --- | --- | --- | --- | --- | --- | --- | --- |
| *B. crocidurae* | 400km^2^ | 0·889 (0·883‒0·895) | 0·960 (0·915‒0·989) | 0·919 (0·908‒0·929) | 0·879 (0·872‒0·886) | 0·803 (0·195‒0·986) | 0·727 (0·713‒0·741) |
|  | 900km^2^ | 0·897 (0·891‒0·902) | 0·963 (0·918‒0·990) | 0·914 (0·903‒0·924) | 0·891 (0·884‒0·897) | 0·813 (0·201‒0·987) | 0·742 (0·728‒0·756) |
| *B. hermsii* | 400km^2^ | 0·926 (0·919‒0·933) | 0·972 (0·930‒0·998) | 0·983 (0·975‒0·988) | 0·907 (0·898‒0·916) | 0·870 (0·239‒0·993) | 0·819 (0·802‒0·836) |
|  | 900km^2^ | 0·930 (0·923‒0·936) | 0·975 (0·944‒0·999) | 0·979 (0·971‒0·985) | 0·914 (0·905‒0·922) | 0·875 (0·243‒0·994) | 0·827 (0·812‒0·843) |
| *B. lonestari* | 400km^2^ | 0·934 (0·927‒0·940) | 0·973 (0·924‒0·997) | 0·974 (0·965‒0·981) | 0·921 (0·913‒0·929) | 0·879 (0·246‒0·994) | 0·835 (0·819‒0·851) |
|  | 900km^2^ | 0·968 (0·964‒0·973) | 0·987 (0·961‒0·998) | 0·985 (0·977‒0·990) | 0·963 (0·957‒0·968) | 0·940 (0·291‒0·998) | 0·919 (0·907‒0·930) |
| *B. miyamotoi* | 400km^2^ | 0·932 (0·929‒0·934) | 0·978 (0·963‒0·988) | 0·935 (0·930‒0·940) | 0·930 (0·927‒0·933) | 0·872 (0·240‒0·993) | 0·826 (0·819‒0·833) |
|  | 900km^2^ | 0·936 (0·933‒0·938) | 0·980 (0·966‒0·990) | 0·945 (0·940‒0·950) | 0·933 (0·930‒0·936) | 0·880 (0·246‒0·994) | 0·837 (0·830‒0·843) |

# Supplementary figure 1: The networks of co-occurring symptoms in the relapsing fever cases. The size of the points indicates the frequency of symptom that are reported alone. The thickness of the lines indicates the frequency of two connected symptoms that are simultaneously reported.

#
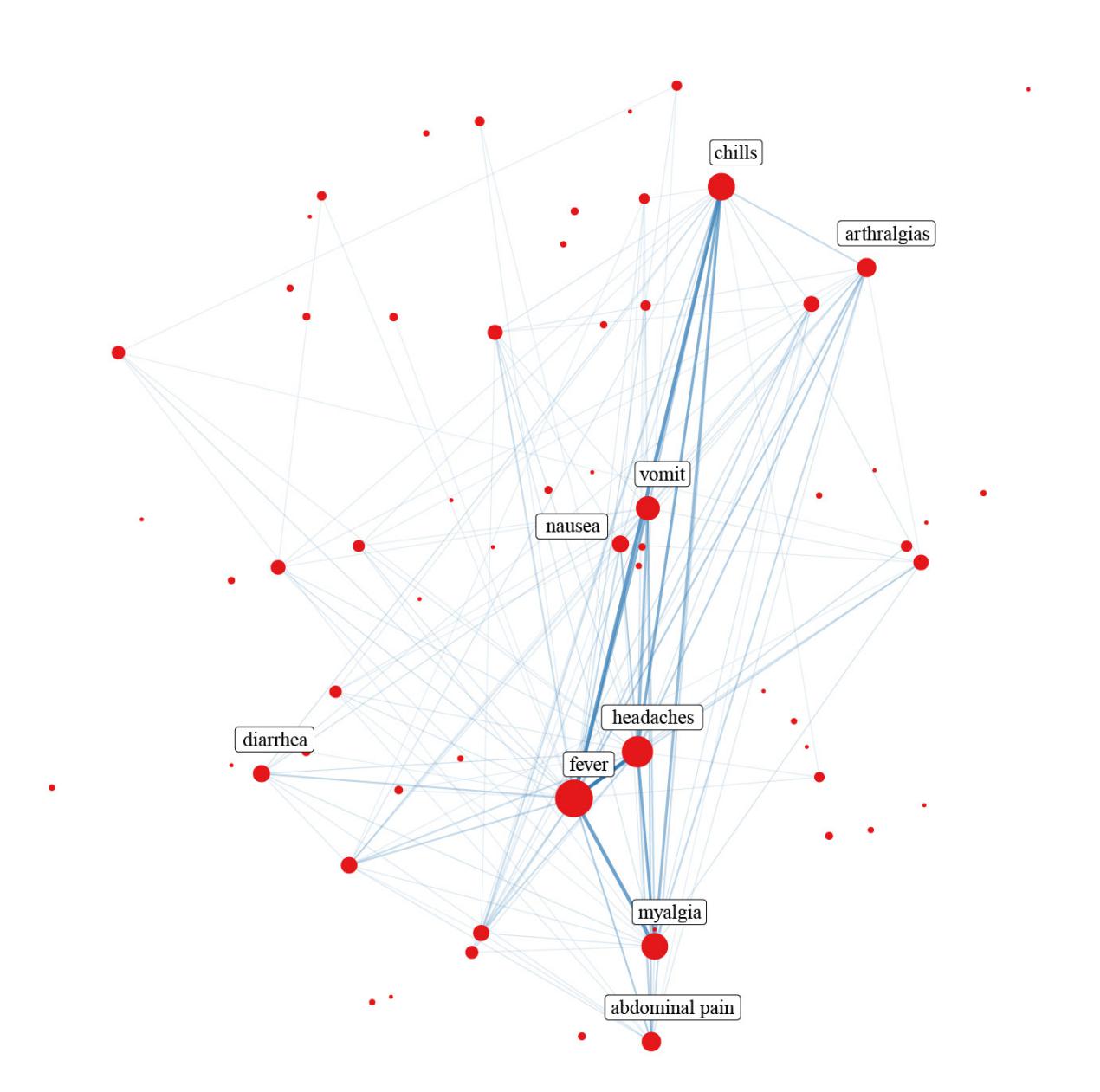


Supplementary figure 2: Effects of major predictors (RCs >3%) for presence of *Ixodes persulcatus* based on BRT models. The mean curves (red) and 95% percentiles (blue, ecoclimatic variables; red, environmental variables; yellow, biological variables) show the predicted habitat suitability index. The histograms show the frequency distributions of the predictors.


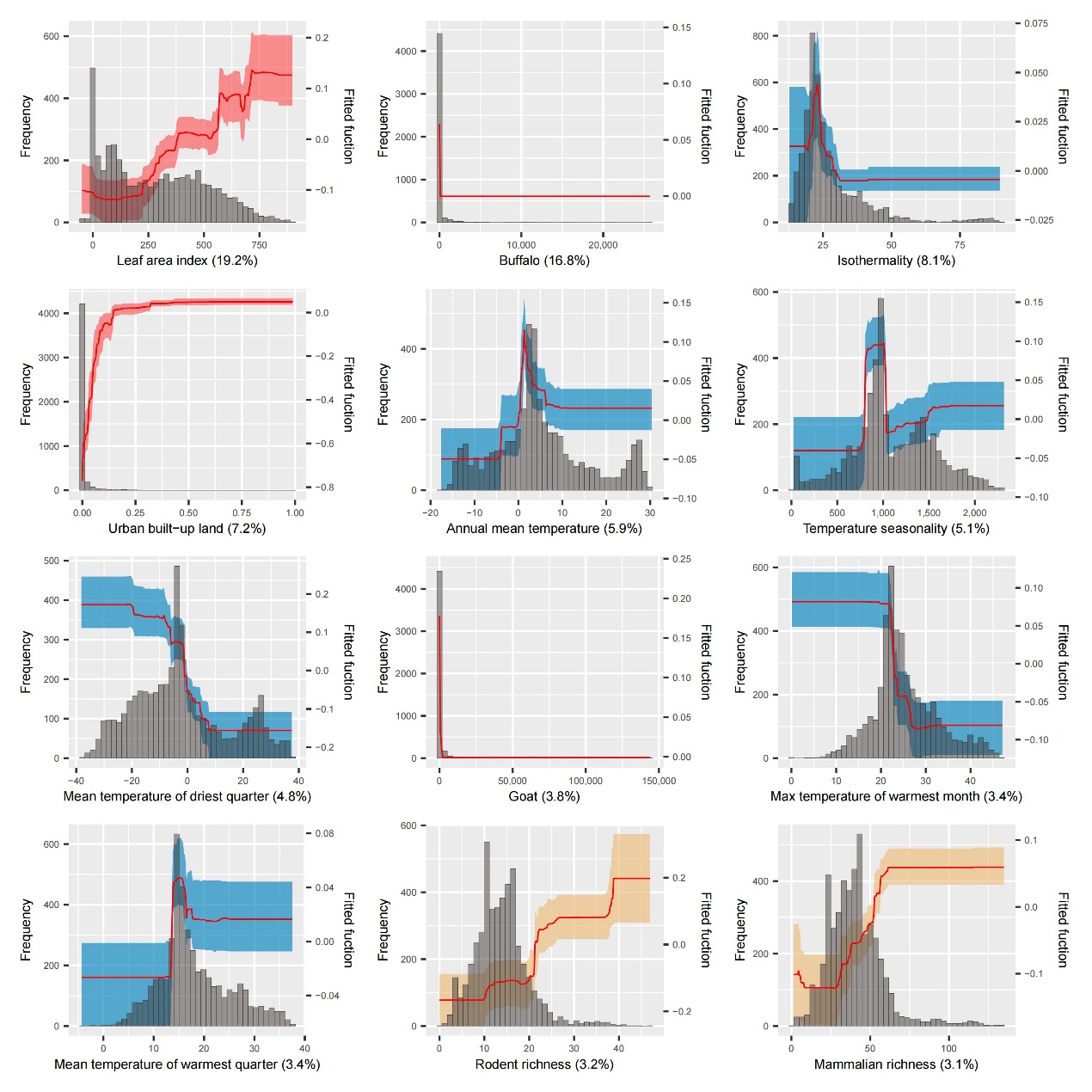


RC, relative contribution. BRT, boosted regression trees.

Supplementary figure 3: Effects of major predictors (RCs >3%) for presence of *Ixodes* *ricinus* based on BRT models. The mean curves (red) and 95% percentiles (blue, ecoclimatic variables; red, environmental variables; yellow, biological variables) show the predicted habitat suitability index. The histograms show the frequency distributions of the predictors.


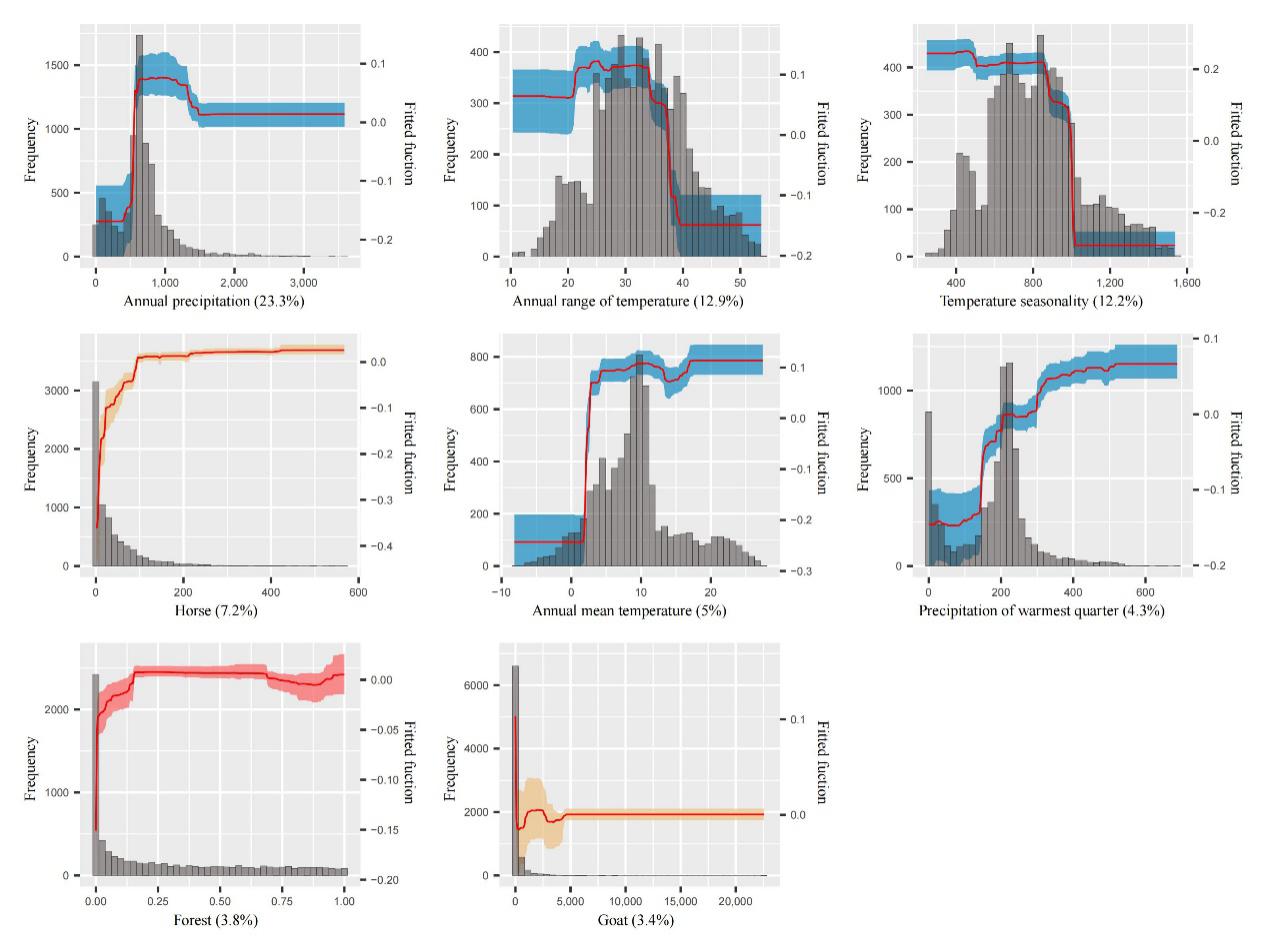


RC, relative contribution. BRT, boosted regression trees.

Supplementary figure 4: Effects of major predictors (RCs >3%) for presence of *Ixodes* *scapularis* based on BRT models. The mean curves (red) and 95% percentiles (blue, ecoclimatic variables; red, environmental variables) show the predicted habitat suitability index. The histograms show the frequency distributions of the predictors.


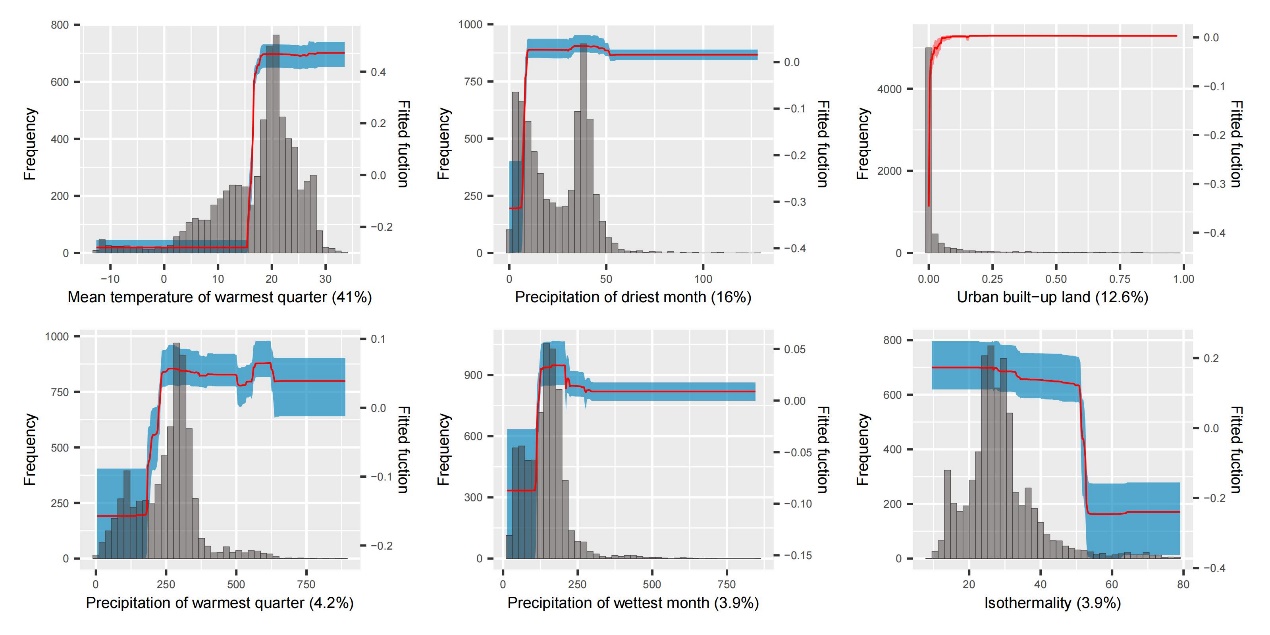


RC, relative contribution. BRT, boosted regression trees.

Supplementary figure 5: Effects of major predictors (RCs >3%) for presence of *Ixodes* *pacificus* based on BRT models. The mean curves (red) and 95% percentiles (blue, ecoclimatic variables) show the predicted habitat suitability index. The histograms show the frequency distributions of the predictors.


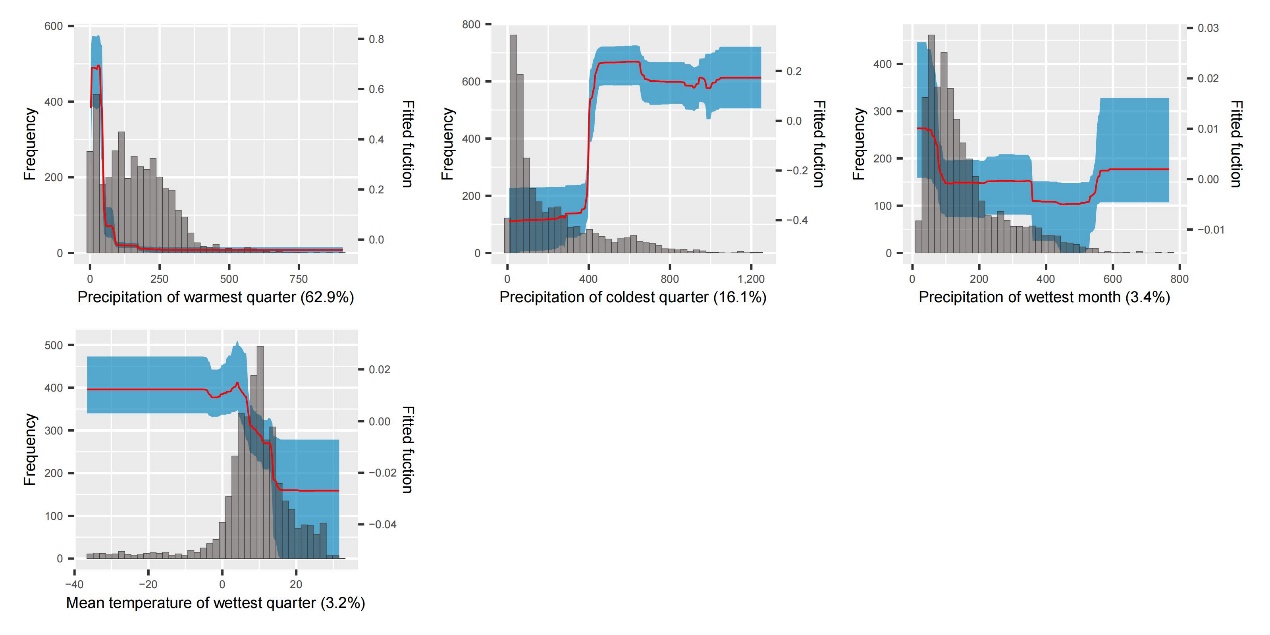


RC, relative contribution. BRT, boosted regression trees.

Supplementary figure 6: Effects of major predictors (RCs >3%) for presence of *Amblyomma americanum* based on BRT models. The mean curves (red) and 95% percentiles (blue, ecoclimatic variables) show the predicted habitat suitability index. The histograms show the frequency distributions of the predictors.


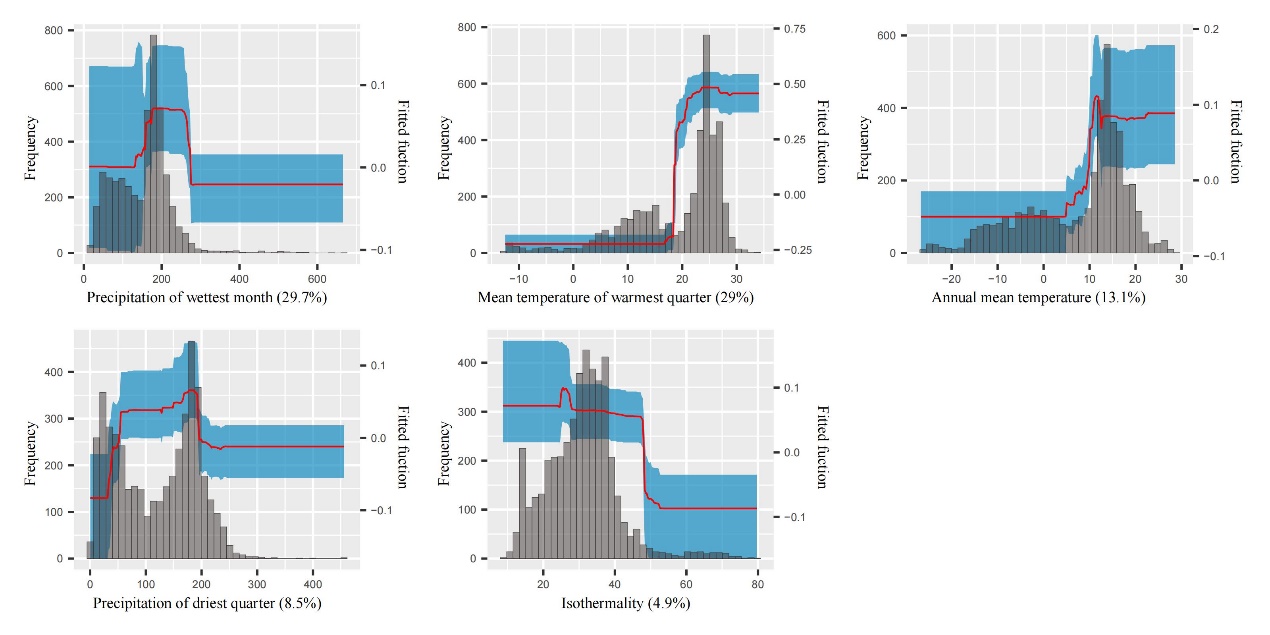


RC, relative contribution. BRT, boosted regression trees.

Supplementary figure 7: Effects of major predictors (RCs >3%) for presence of *Ornithodoros hermsi* based on BRT models. The mean curves (red) and 95% percentiles (blue, ecoclimatic variables; yellow, biological variables) show the predicted habitat suitability index. The histograms show the frequency distributions of the predictors.


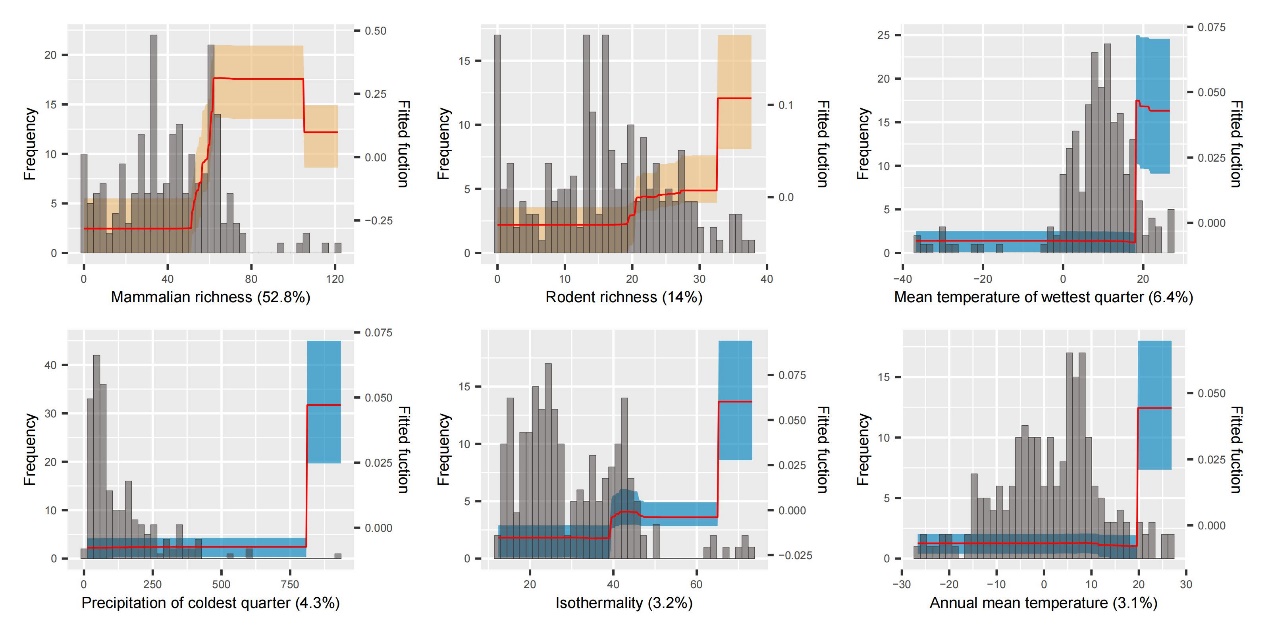


RC, relative contribution. BRT, boosted regression trees.

Supplementary figure 8: Effects of major predictors (RCs >3%) for presence of *Ornithodoros* *sonrai* based on BRT models. The mean curves (red) and 95% percentiles (blue, ecoclimatic variables; red, environmental variables; yellow, biological variables) show the predicted habitat suitability index. The histograms show the frequency distributions of the predictors.


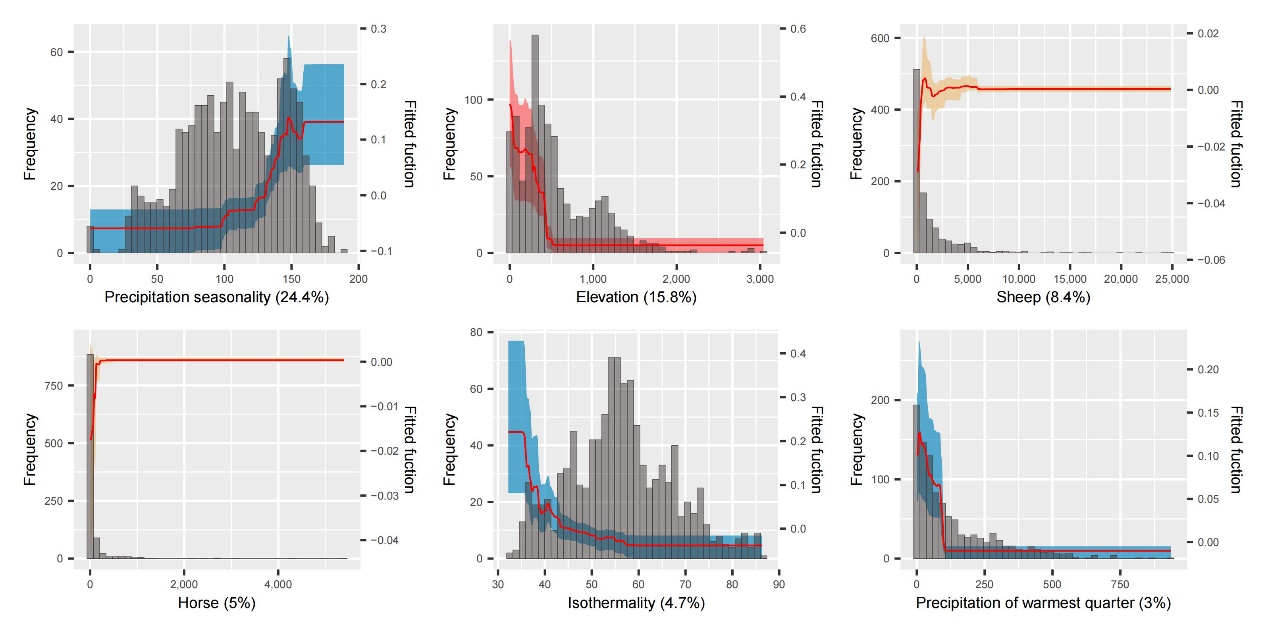


RC, relative contribution. BRT, boosted regression trees.

Supplementary figure 9: The predicted and recorded distributions of *Ixodes persulcatus* in Eurasia. (A) Recorded locations of *Ixodes persulcatus*. (B) Predicted HSI of *Ixodes persulcatus* based on the BRT model. (C) Predicted HSI and relative uncertainty into four segments by their corresponding 80th, 90th and 95th percentiles, respectively, with the colors from light to deep representing the values from low to high.


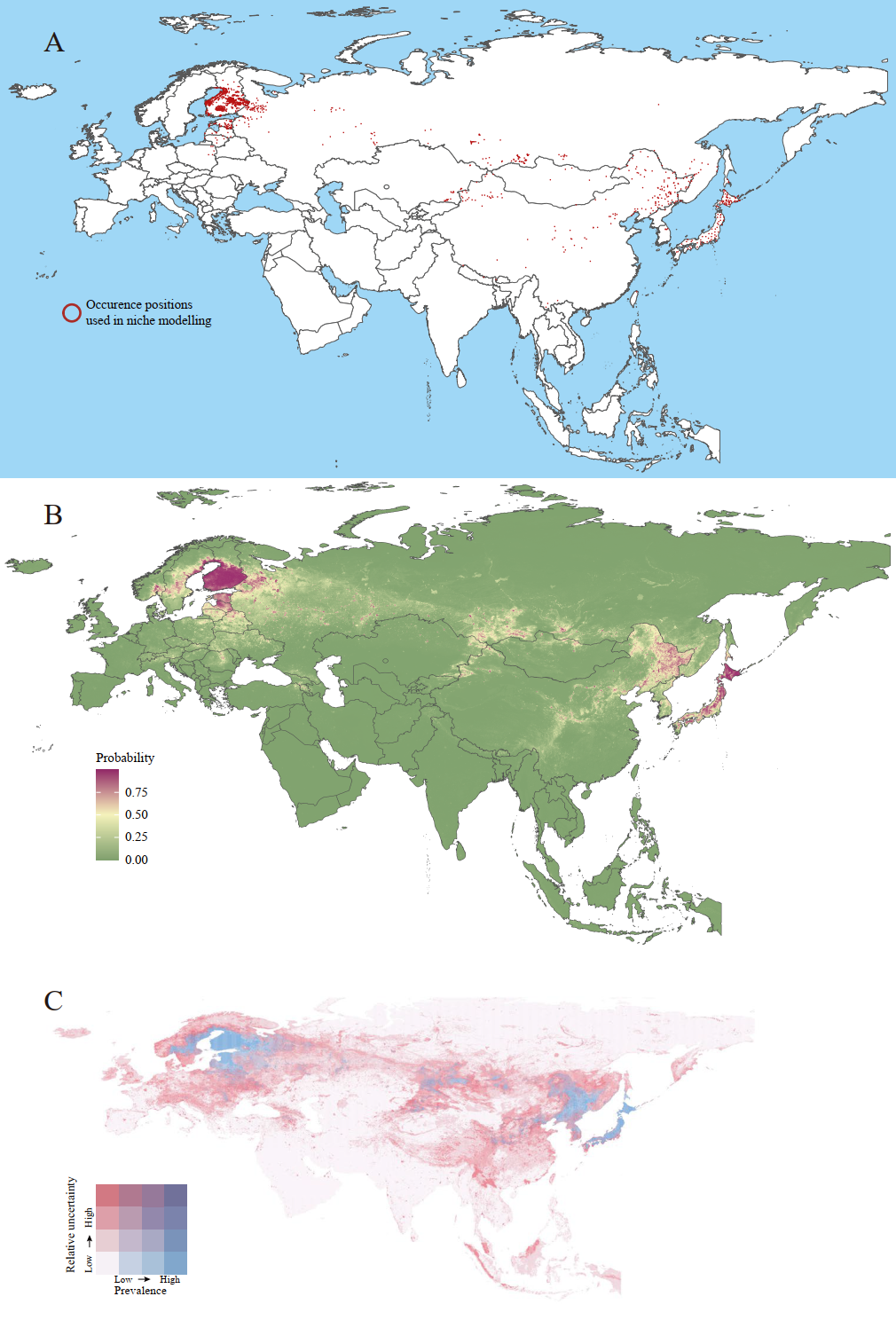


Supplementary figure 10: The regional predicted and recorded distributions of *Ixodes ricinus*. (A) Recorded locations of *Ixodes ricinus*. (B) Predicted HSI of *Ixodes ricinus* based on the BRT model. (C) Predicted HSI and relative uncertainty into four segments by their corresponding 80th, 90th and 95th percentiles, respectively, with the colors from light to deep representing the values from low to high.


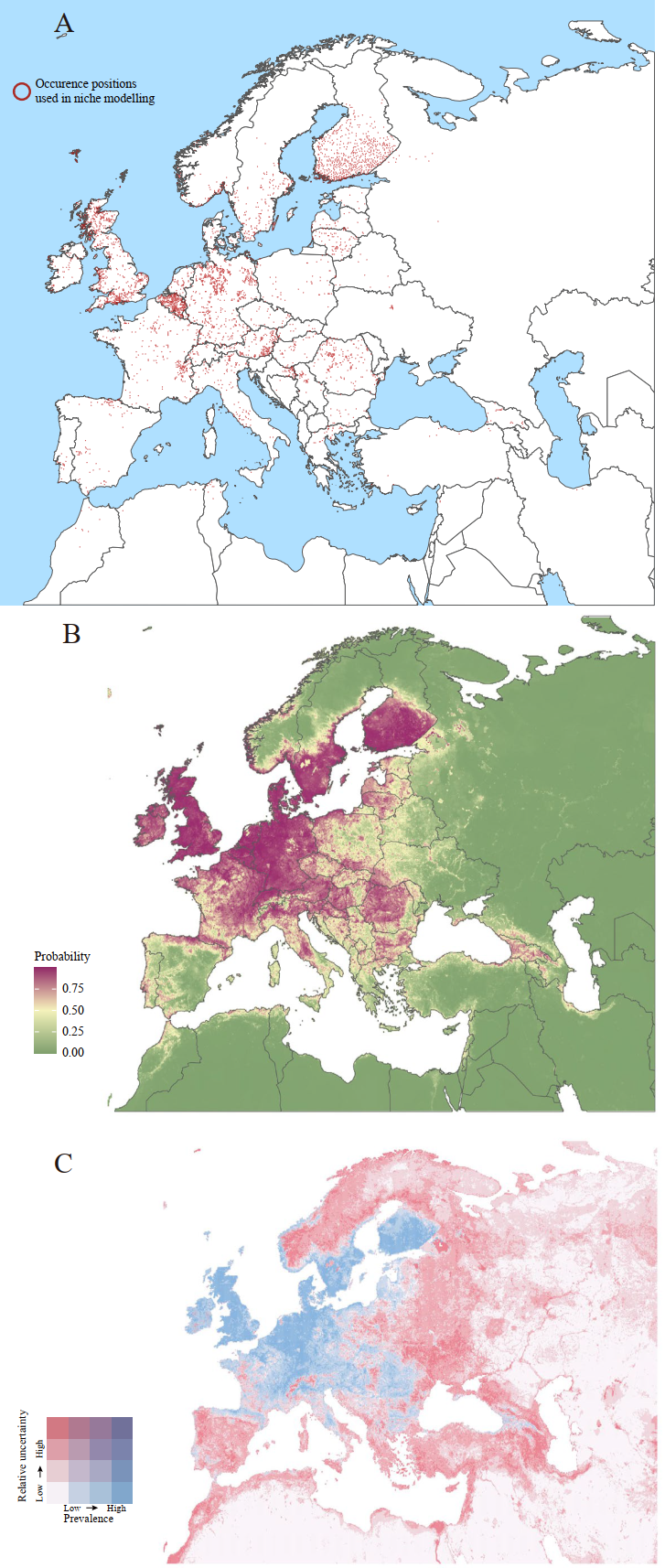


Supplementary figure 11: The predicted and recorded distributions of *Ixodes scapularis* in North America. (A) Recorded locations of *Ixodes scapularis*. (B) Predicted HSI of *Ixodes scapularis* based on the BRT model. (C) PredicthisHSI and relative uncertainty into four segments by their correspond^in^g 80^th^, 90th ^an^d 95th percentiles, respectively, with the colors from light to deep representing the values from low to high.


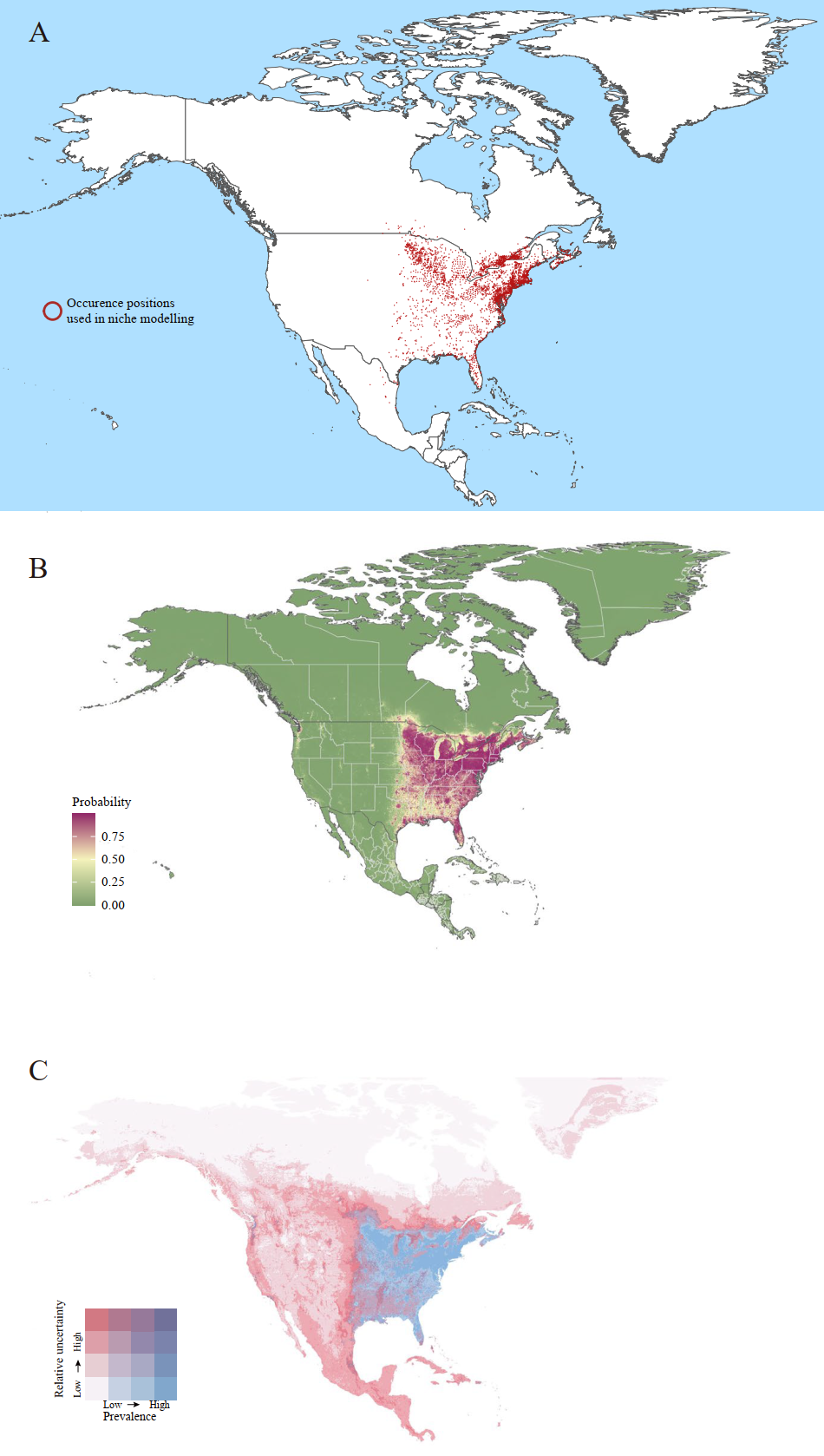


Supplementary figure 12: The predicted and recorded distributions of *Ixodes pacificus* in North America. (A) Recorded locations of *Ixodes pacificus*. (B) Predicted HSI of *Ixodes pacificus* based on the BRT model. (C) Predicted HSI and relative uncertainty into four segments by their corresponding 80th, 90th and 95th percentiles, respectively, with the colors from light to deep representing the values from low to high.


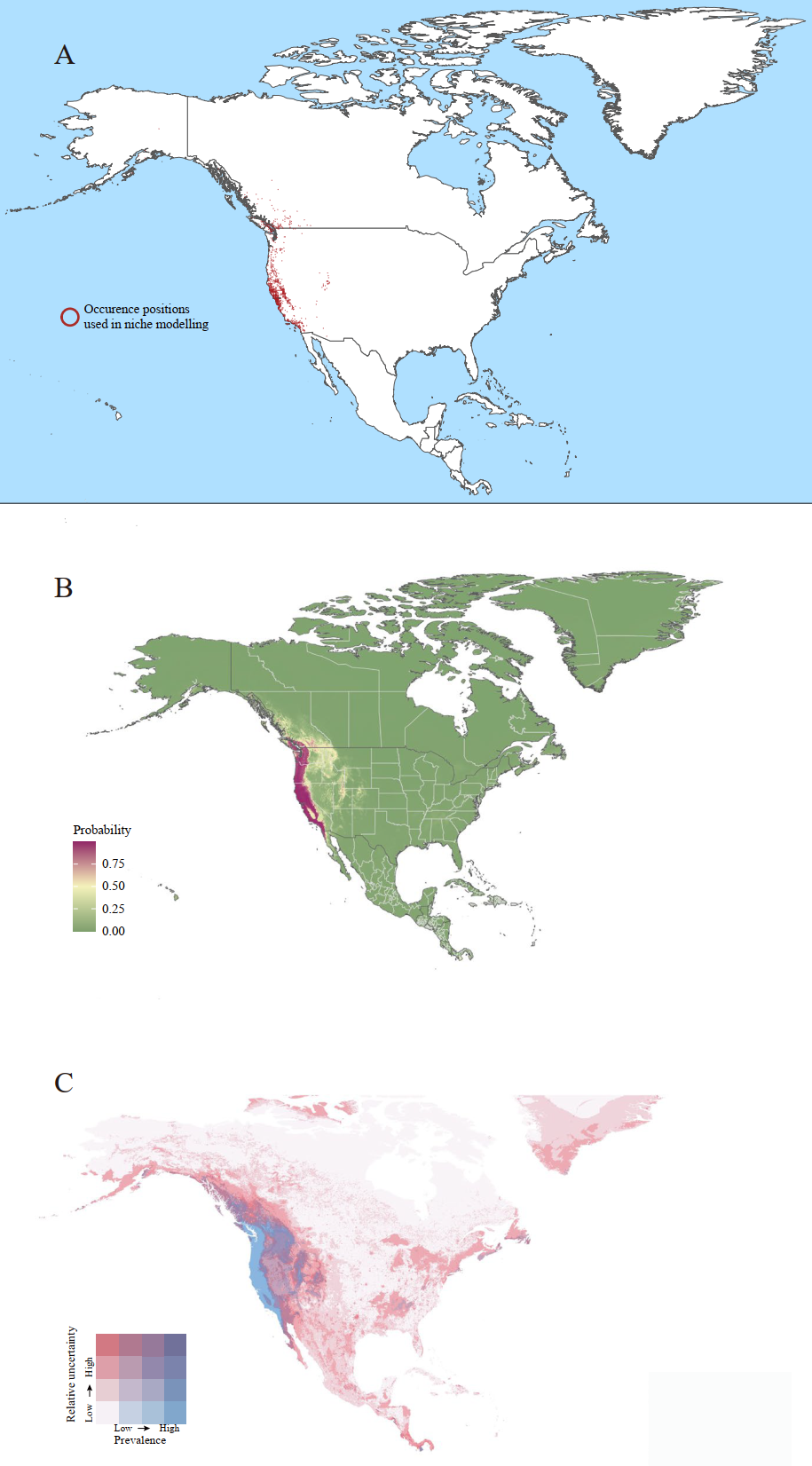


Supplementary figure 13: The predicted and recorded distributions of *Amblyomma americanum* in North America. (A) Recorded locations of *Amblyomma americanum.* (B) Predicted HSI of *Amblyomma americanum* based on the BRT model. (C) Predicted HSI and relative uncertainty into four segments by their corresponding 80th, 90th and 95th percentiles, respectively, with the colors from light to deep representing the values from low to high.


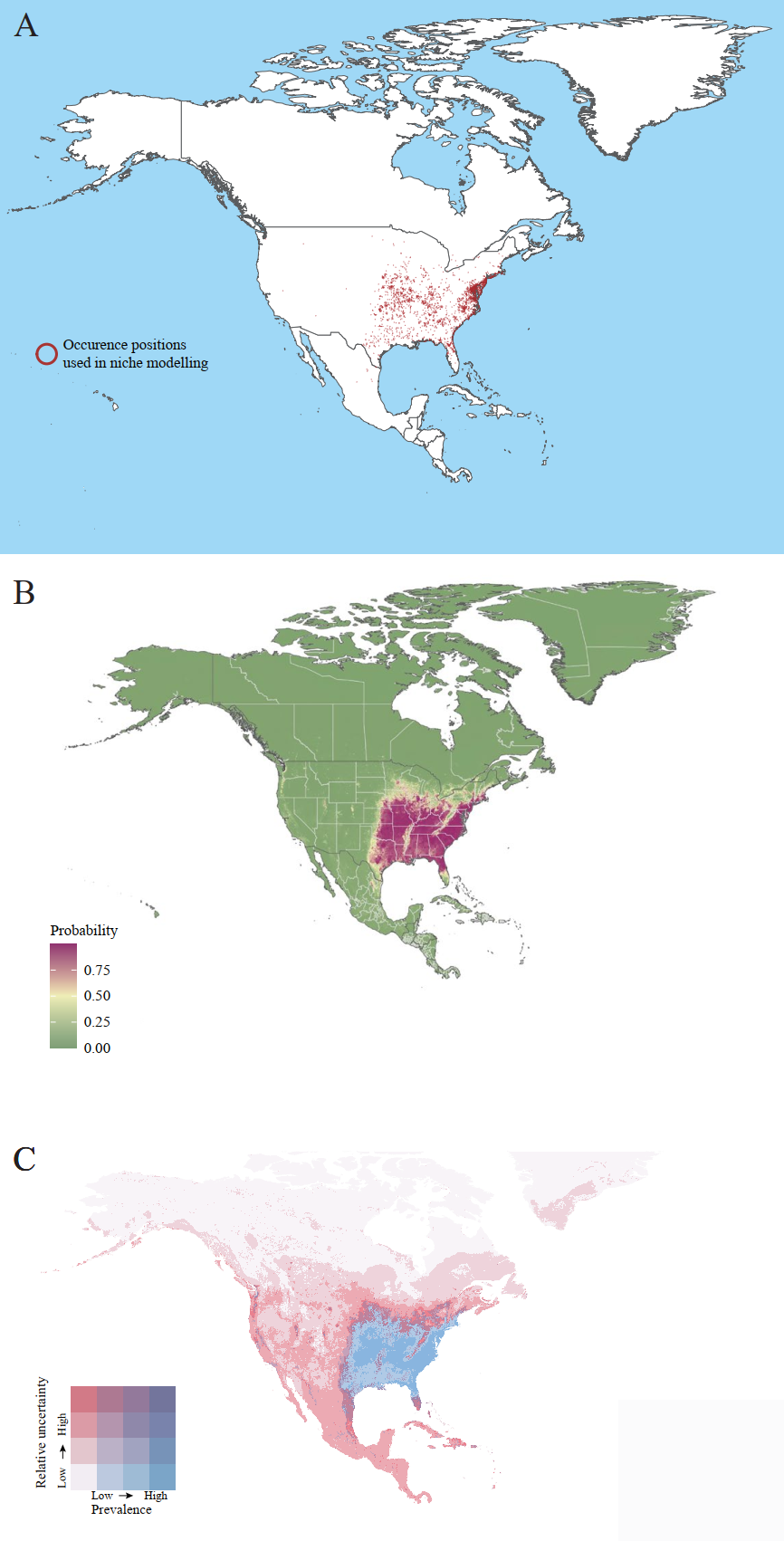


Supplementary figure 14: The predicted and recorded distributions of *Ornithodoros hermsi* in North America. (A) Recorded locations of *Ornithodoros hermsi.* (B) Predicted HSI of *Ornithodoros hermsi* based on the BRT model. (C) Predicted HSI and relative uncertainty into four segments by their corresponding 80th, 90th and 95th percentiles, respectively, with the colors from light to deep representing the values from low to high.


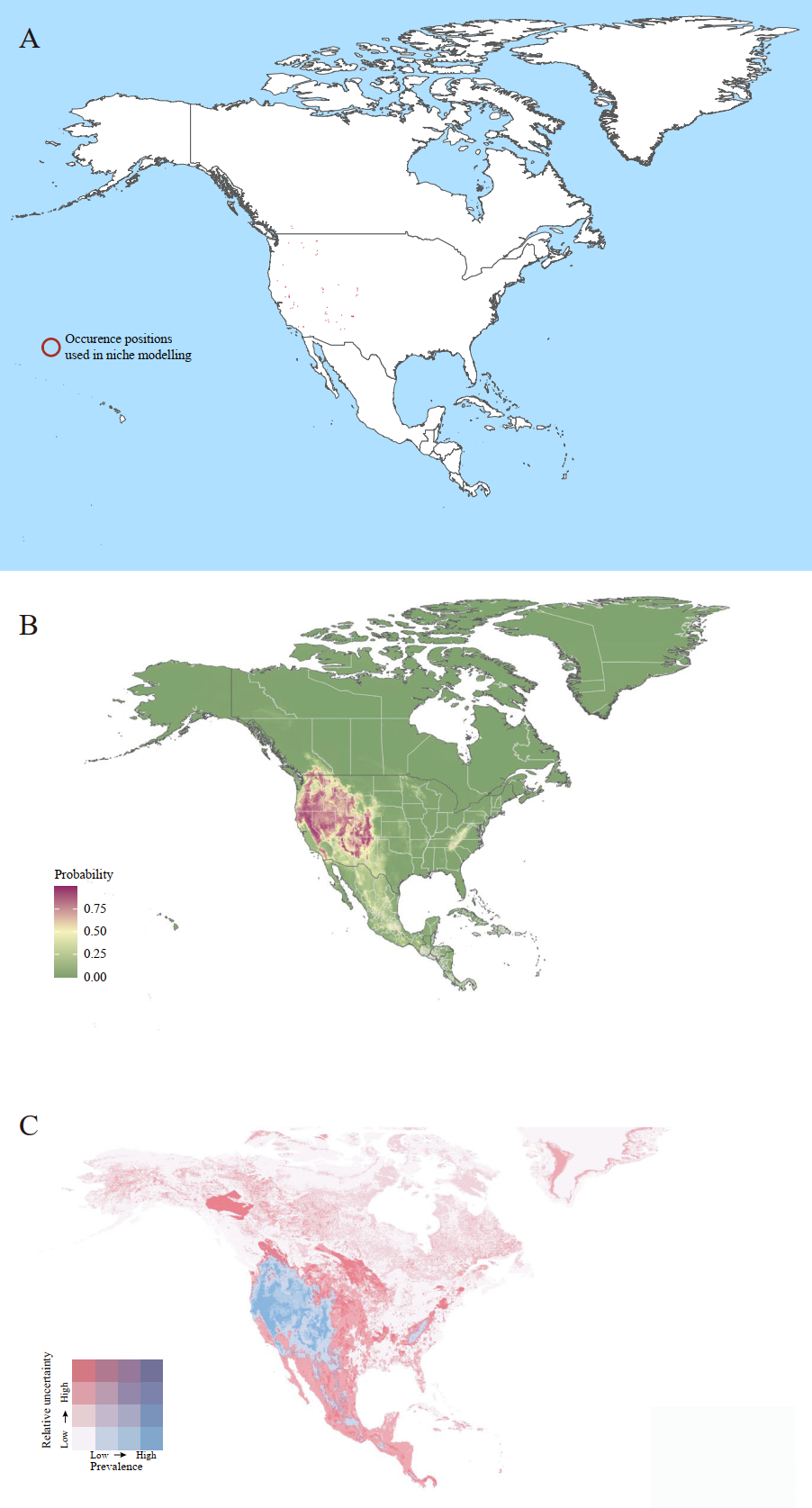


Supplementary figure 15: The predicted and recorded distributions of *Ornithodoros sonrai* in Africa. (A) Recorded locations of *Ornithodoros sonrai.* (B) Predicted HSI of *Ornithodoros sonrai* based on the BRT model. (C) Predicted HSI and relative uncertainty into four segments by their corresponding 80th, 90th and 95th percentiles, respectively, with the colors from light to deep representing the values from low to high.


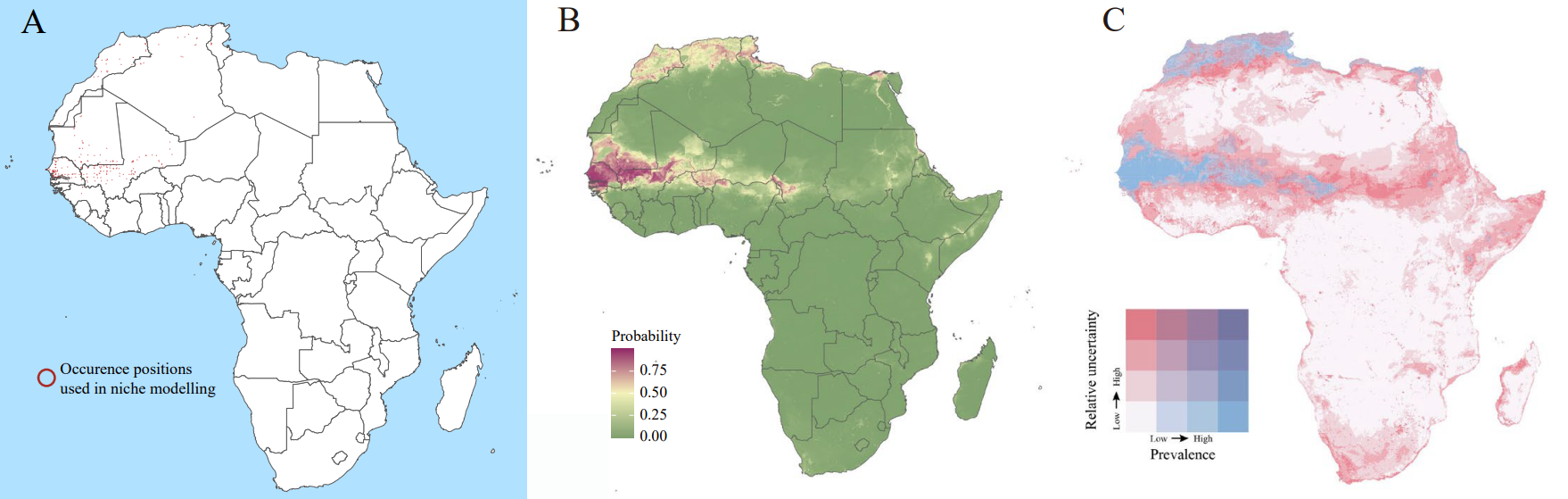


Supplementary figure 16: Predictive performance of the three machine-learning algorithms. ROC curves and AUC values of the BRT (left, red), RF (middle, orange) and LASSO regression (right, blue) over 100 models are shown. Roman numerals 1-4 correspond to the four major RFGB species: (I) *Borrelia miyamotoi*; (II) *Borrelia lonestari*; (III) *Borrelia hermsii*; (IV) *Borrelia crocidurae*.


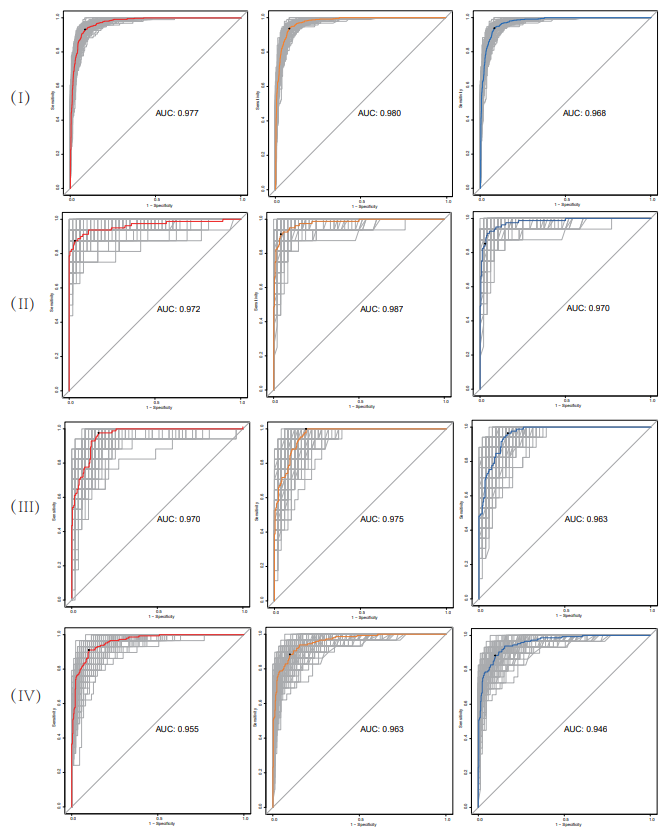


ROC, receiver operating characteristic. AUC, area under the curve. BRT, boosted regression trees. RF, random forest. LASSO, least absolute shrinkage and selection operator. RFGB, relapsing fever group *Borrelia*.

Supplementary figure 17: Effects of major predictors (RCs >3%) for presence of *Borrelia miyamotoi* based on RF models. The mean curves (red) and 95% percentiles (blue, ecoclimatic variables; red, environmental variables; yellow, biological variables; green, socioeconomic variables) show the predicted probability of occurrence. The histograms show the frequency distributions of the predictors.


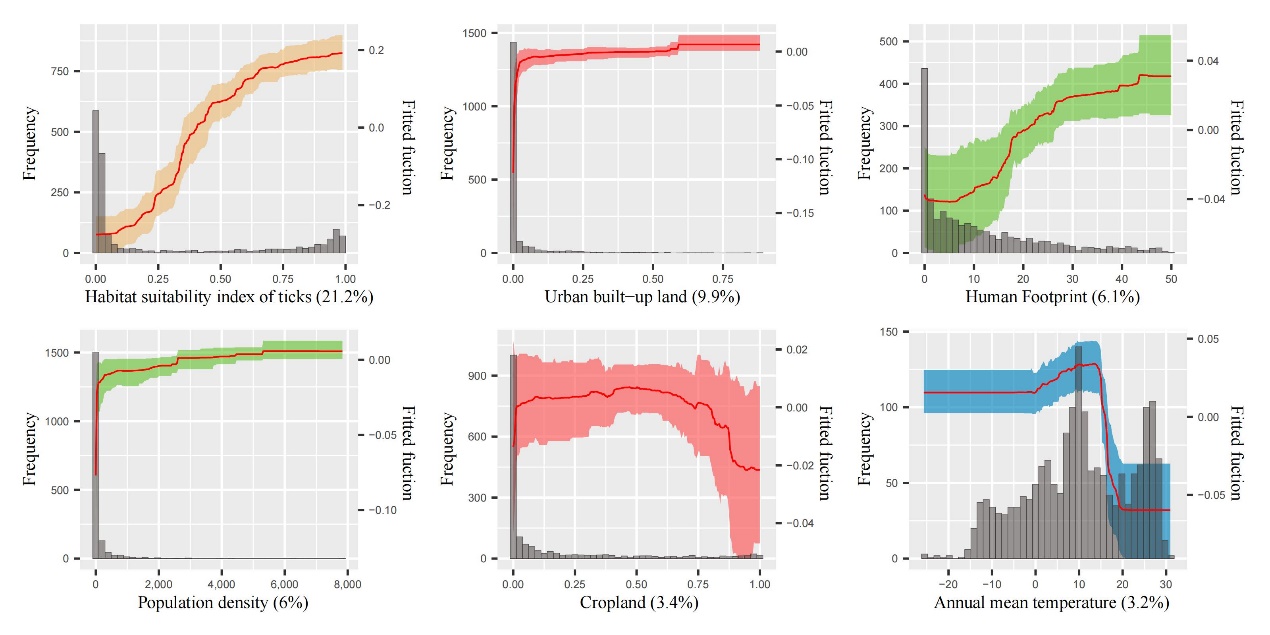


RC, relative contribution. RF, random forest.

Supplementary figure 18: Effects of major predictors (RCs >3%) for presence of *Borrelia lonestari* based on RF models. The mean curves (red) and 95% percentiles (blue, ecoclimatic variables; yellow, biological variables) show the predicted probability of occurrence. The histograms show the frequency distributions of the predictors.

**
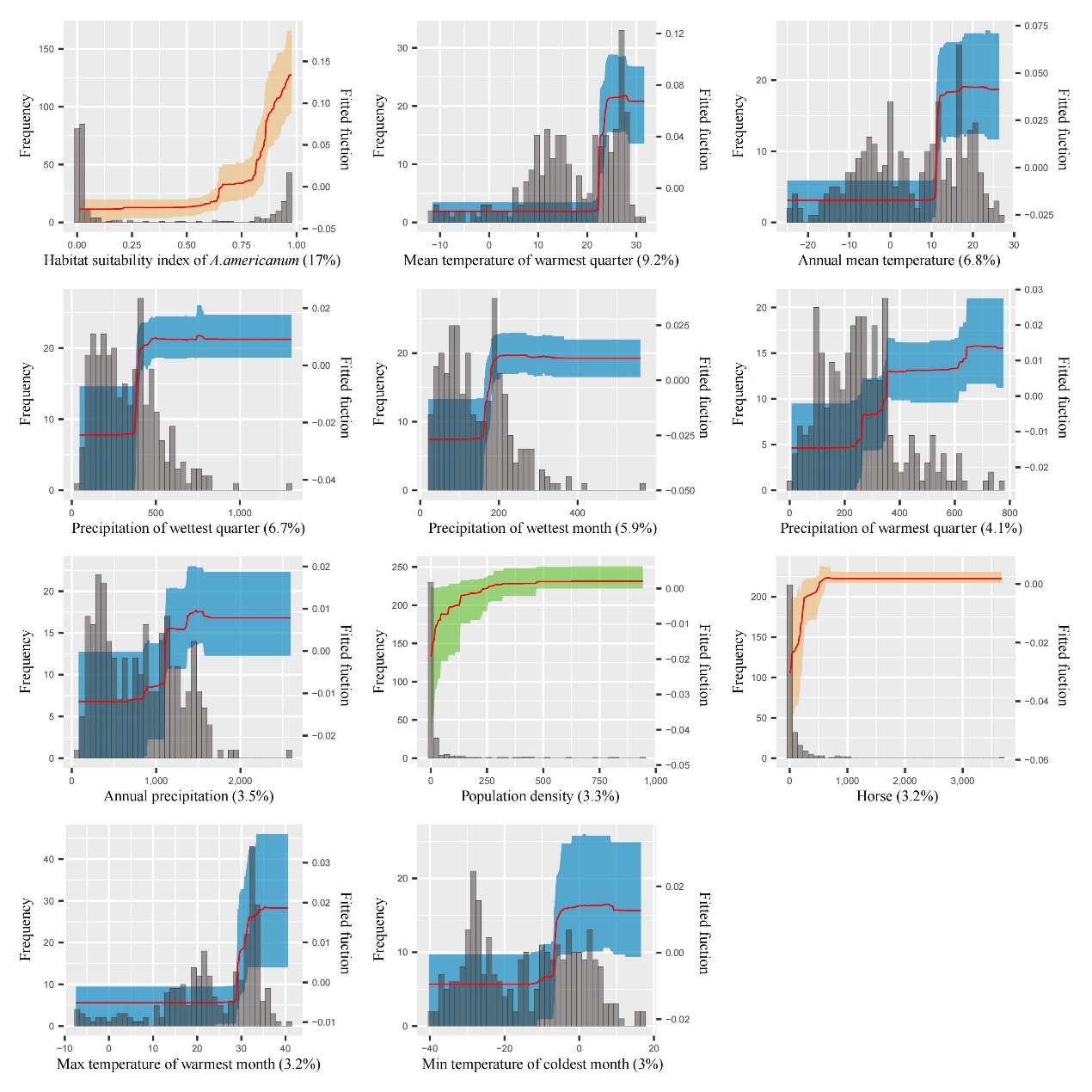
**

RC, relative contribution. RF, random forest.

Supplementary figure 19: Effects of major predictors (RCs >3%) for presence of *Borrelia hermsii* based on RF models. The mean curves (red) and 95% percentiles (blue, ecoclimatic variables; yellow, biological variables; green, socioeconomic variables) show the predicted probability of occurrence. The histograms show the frequency distributions of the predictors.

**
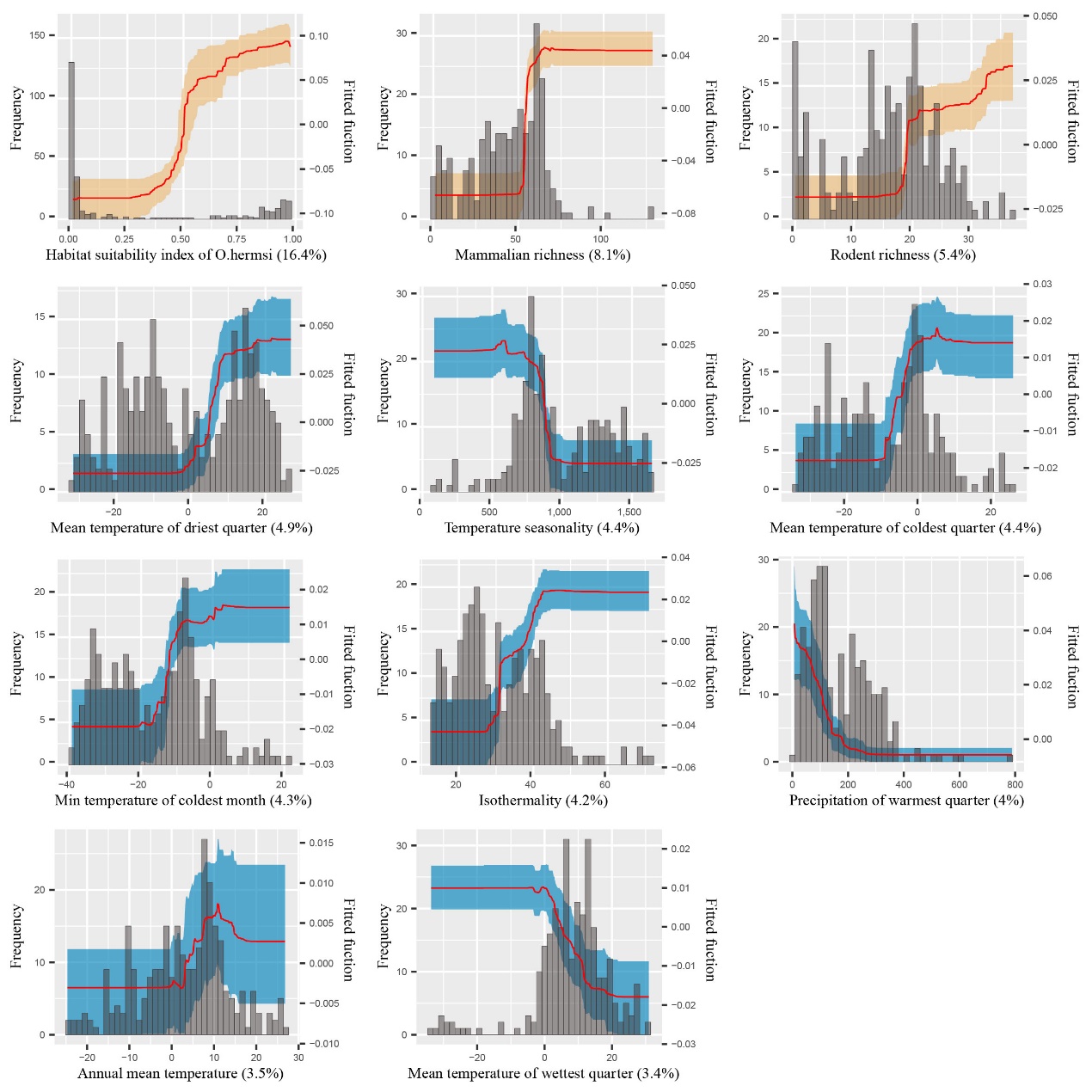
**

RC, relative contribution. RF, random forest.

Supplementary figure 20: Effects of major predictors (RCs >3%) for presence of *Borrelia crocidurae* based on RF models. The mean curves (red) and 95% percentiles (blue, ecoclimatic variables; red, environmental variables; yellow, biological variables) show the predicted probability of occurrence. The histograms show the frequency distributions of the predictors.

**
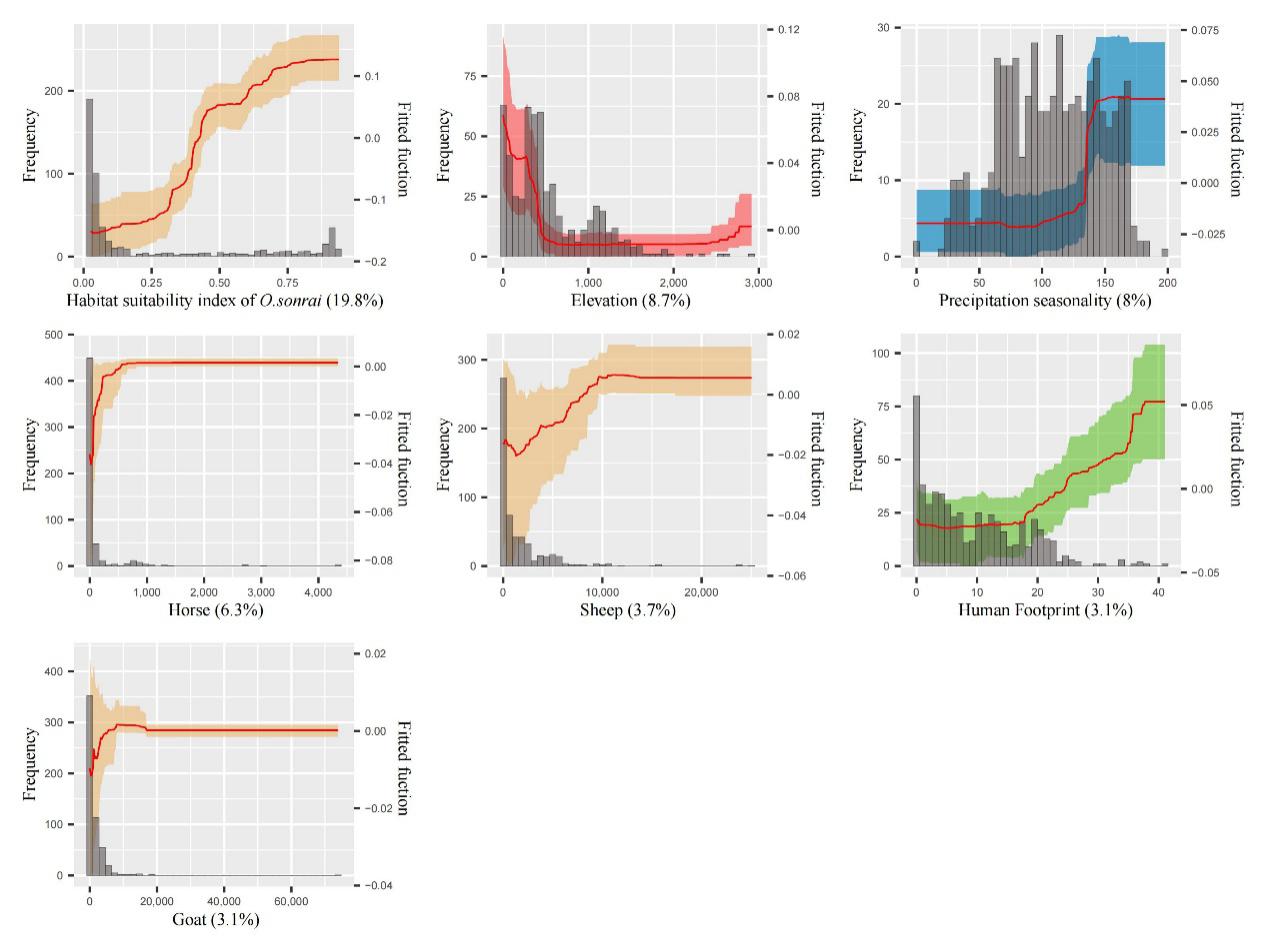
**

RC, relative contribution. RF, random forest.

Supplementary figure 21: The predicted and recorded distributions of *Borrelia miyamotoi* within global range. (A) Predicted risk probability of *Borrelia miyamotoi* based on the random forest model. (B) Recorded locations of *Borrelia miyamotoi* detected from arthropod vectors, animals and humans.


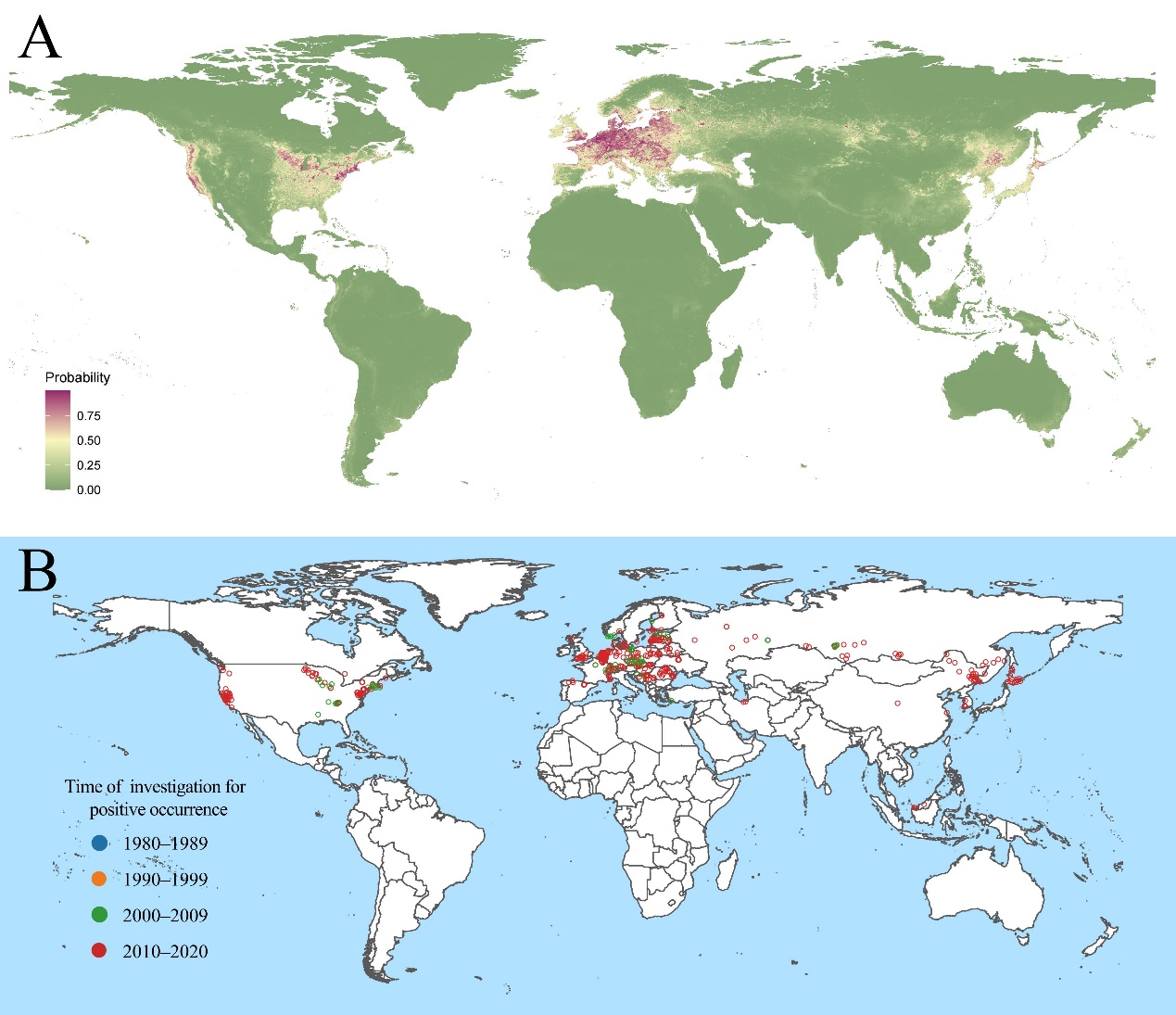


Supplementary figure 22: The predicted and recorded distributions of *Borrelia lonestari* in North America. (A) Predicted risk probability of *Borrelia lonestari* based on the random forest model. (B) Recorded locations of *Borrelia lonestari* detected from arthropod vectors, animals and humans.


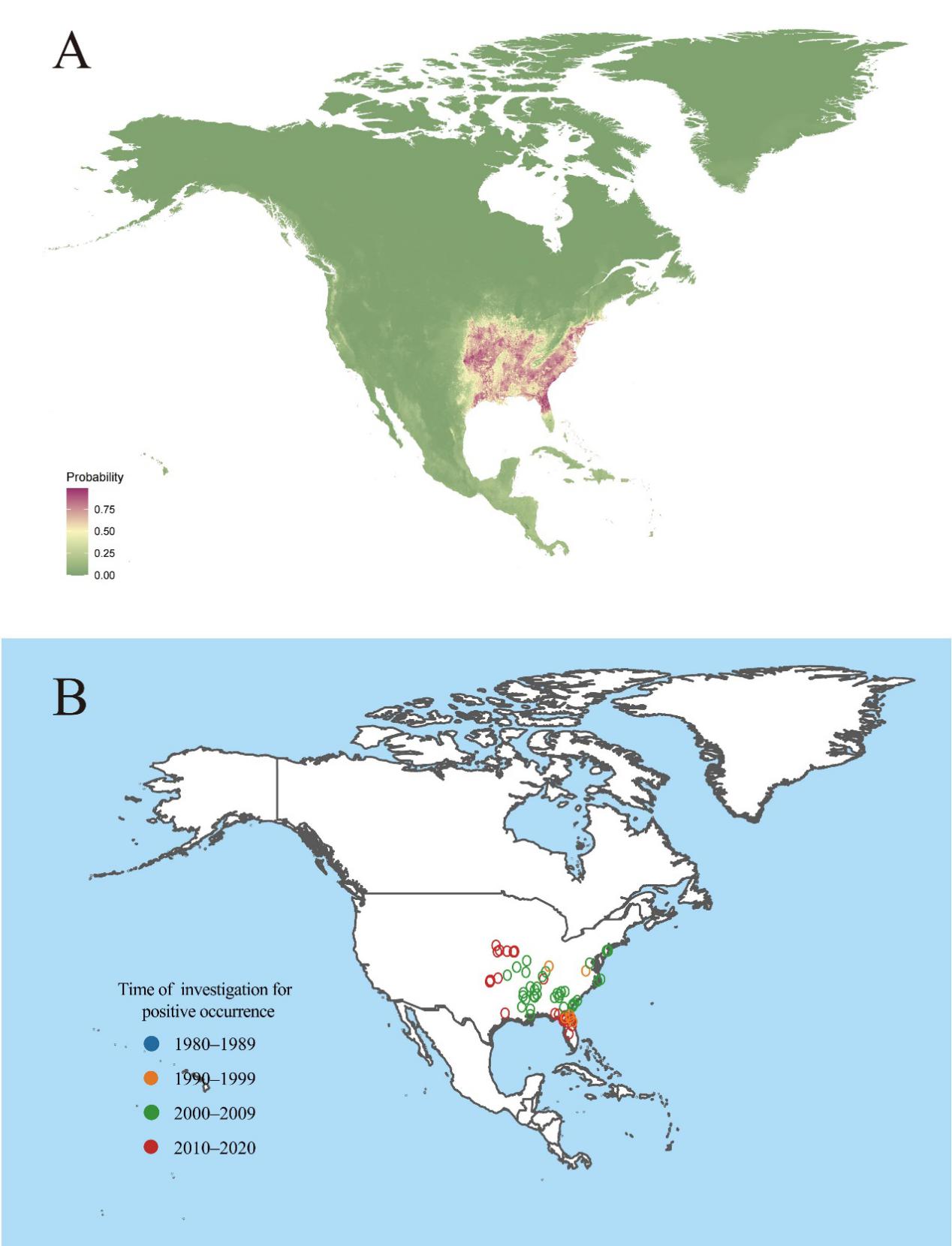


Supplementary figure 23: The predicted and recorded distributions of *Borrelia hermsii* in North America. (A) Predicted risk probability of *Borrelia hermsii* based on the random forest model. (B) Recorded locations of *Borrelia hermsii* detected from arthropod vectors, animals and humans.


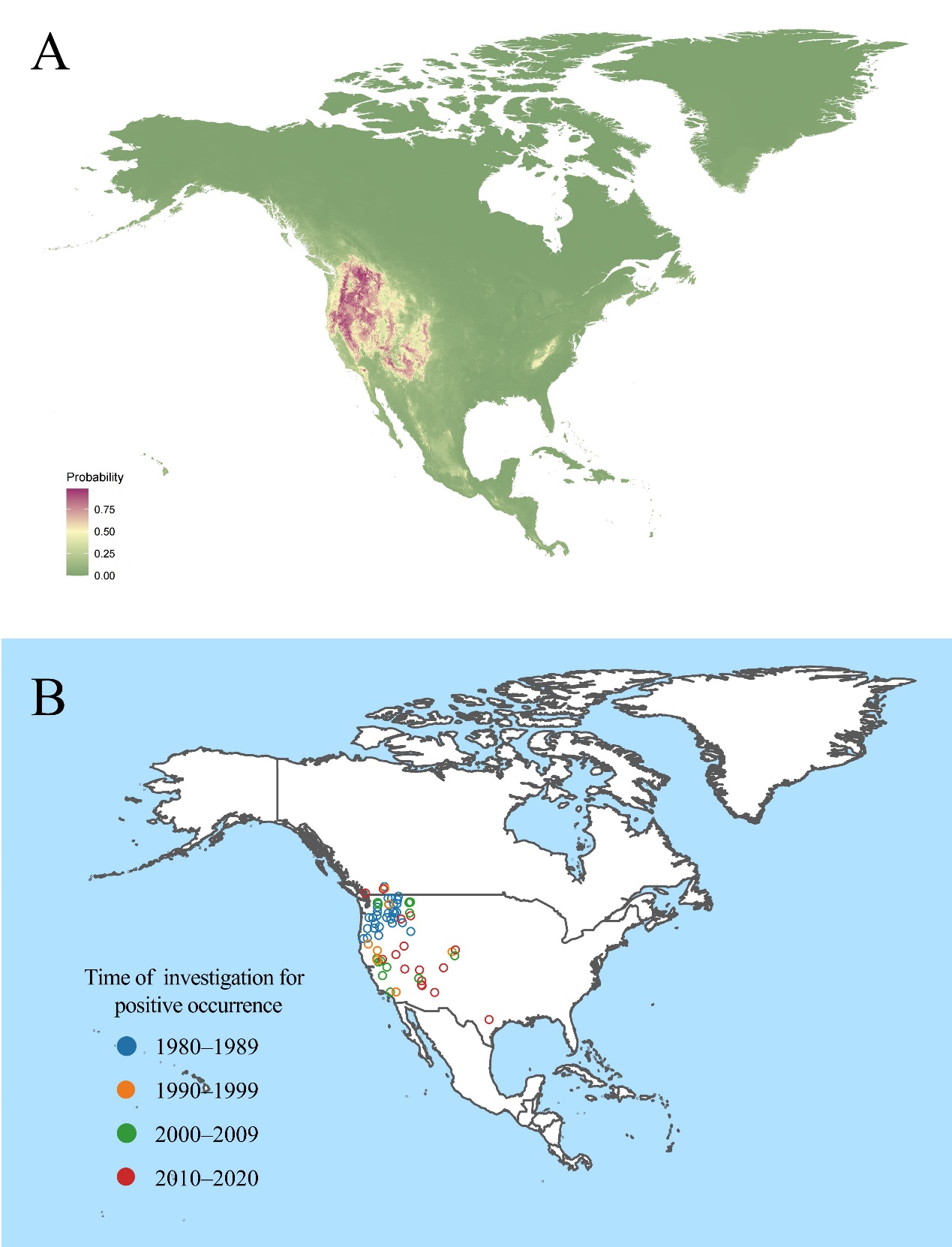


Supplementary figure 24: The predicted and recorded distributions of *Borrelia crocidurae* in Africa. (A) Predicted risk probability of *Borrelia crocidurae* based on the random forest model. (B) Recorded locations of *Borrelia crocidurae* detected from arthropod vectors, animals and humans.


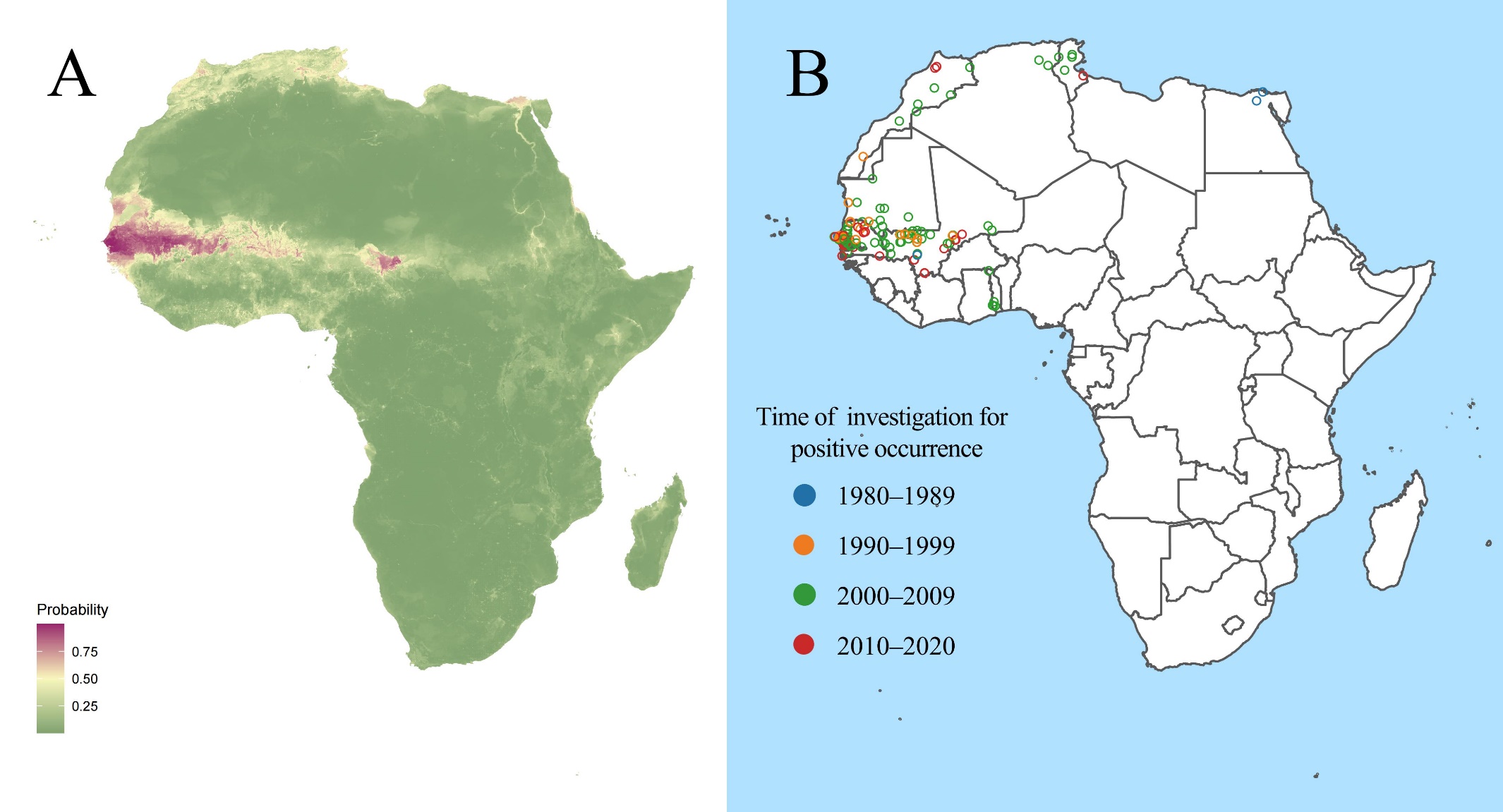


# Supplementary References

1. Theodoulou M. Tick-borne relapsing fever in Cyprus. *Cyprus Med J* 1952; **5**: 860–62.

2. Vázquez-Guerrero E, Adan-Bante NP, Mercado-Uribe MC, et al. Case report: a retrospective serological analysis indicating human exposure to tickborne relapsing fever spirochetes in Sonora, Mexico. *PLoS Negl Trop Dis* 2019; **13**: e0007215.

3. Thompson RS, Burgdorfer W, Russell R, Francis BJ. Outbreak of tick-borne relapsing fever in Spokane county, Washington. *JAMA* 1969; **210**: 1045–50.

4. Moemenbellah-Fard MD, Benafshi O, Rafinejad J, Ashraf H. Tick-borne relapsing fever in a new highland endemic focus of western Iran. *Ann Trop Med Parasitol* 2009; **103**: 529–37.

5. McNamara JJ, Kay HH. Relapsing fever (*Borrelia*) in an adolescent tourist in Israel. *J Adolesc Health Care* 1988; **9**: 421–23.

6. Xu G, Luo CY, Ribbe F, et al. *Borrelia miyamotoi* in human-biting ticks, United States, 2013-2019. *Emerg Infect Dis* 2021; **27**: 3193–95.

7. Wilder HK, Wozniak E, Huddleston E, et al. Case Report: A retrospective serological analysis indicating human exposure to tick-borne relapsing fever spirochetes in Texas. *PLoS Negl Trop Dis* 2015; **9**: e0003617.

8. Vial L, Diatta G, Tall A, et al. Incidence of tick-borne relapsing fever in west Africa: longitudinal study. *Lancet* 2006; **368**: 37–43.

9. Takano A, Toyomane K, Konnai S, et al. Tick surveillance for relapsing fever spirochete *Borrelia miyamotoi* in Hokkaido, Japan. *PLoS One* 2014; **9**: e104532.

10. Sundnes KO, Haimanot AT. Haimanot. Epidemic of louse-borne relapsing fever in Ethiopia. *Lancet* 1993; **342**: 1213–15.

11. Simon JW. Tick borne relapsing fever imported into the United Kingdom. *J R Army Med Corps* 1985; **131**: 65–67.

12. Seilmaier M, Guggemos W, Wieser A, et al. Louse-borne-relapsing-fever in refugees from the horn of Africa; a case series of 25 patients. *Dtsch Med Wochenschr* 2016; **141**: e133–42.

13. Scoles GA, Papero M, Beati L, Fish D. A relapsing fever group spirochete transmitted by *Ixodes scapularis* ticks. *Vector Borne Zoonotic Dis* 2001; **1**: 21–34.

14. Schwan TG, Raffel SJ, Schrumpf ME, et al. Tick-borne relapsing fever and *Borrelia hermsii*, Los Angeles County, California, USA. *Emerg Infect Dis* 2009; **15**: 1026–31.

15. Heglasová I, Rudenko N, Golovchenko M, et al. Ticks, fleas and rodent-hosts analyzed for the presence of *Borrelia miyamotoi* in Slovakia: the first record of *Borrelia miyamotoi* in a *Haemaphysalis inermis* tick. *Ticks Tick Borne Dis* 2020; **11**: 101456.

16. Salih SY, Mustafa D, Abdel Wahab SM, Ahmed MA, Omer A. Louse-borne relapsing fever: I. A clinical and laboratory study of 363 cases in the Sudan. *Trans R Soc Trop Med Hyg* 1977; **71**: 43–48.

17. Ruyts SC, Tack W, Ampoorter E, et al. Year-to-year variation in the density of *Ixodes ricinus* ticks and the prevalence of the rodent-associated human pathogens *Borrelia afzelii* and *B. miyamotoi* in different forest types. *Ticks Tick Borne Dis* 2018; **9**: 141–45.

18. Roscoe C, Epperly T. Tick-borne relapsing fever. *Am Fam Physician* 2005; **72**: 2039–44.

19. Paul WS, Maupin G, Scott-Wright AO, Craven RB, Dennis DT. Outbreak of tick-borne relapsing fever at the north rim of the Grand Canyon: evidence for effectiveness of preventive measures. *Am J Trop Med Hyg* 2002; **66**: 71–75.

20. Parola P, Diatta G, Socolovschi C, et al. Tick-borne relapsing fever borreliosis, rural Senegal. *Emerg Infect Dis* 2011; **17**: 883–85.

21. Ndiaye EHI, Diouf FS, Ndiaye M, et al. Tick-borne relapsing fever borreliosis, a major public health problem overlooked in Senegal. *PLoS Negl Trop Dis* 2021; **15**: e0009184.

22. Molloy PJ, Telford SR, 3rd, Chowdri HR, et al. *Borrelia miyamotoi* disease in the Northeastern United States: a case series. *Ann Intern Med* 2015; **163**: 91–98.

23. Masoumi Asl H, Goya MM, Vatandoost H, et al. The epidemiology of tick-borne relapsing fever in Iran during 1997-2006. *Travel Med Infect Dis* 2009; **7**: 160–64.

24. Lucchini A, Lipani F, Costa C, et al. Louseborne relapsing fever among East African refugees, Italy, 2015. *Emerg Infect Dis* 2016; **22**: 298–301.

25. Linnemann CC Jr, Barber LC, Dine MS, Body AE. Tick-borne relapsing fever in the Eastern United States. *Am J Dis Child* 1978; **132**: 40–42.

26. Kutsuna S, Kawabata H, Kasahara K, Takano A, Mikasa K. The first case of imported relapsing fever in Japan. *Am J Trop Med Hyg* 2013; **89**: 460–61.

27. Hashavya S, Gross I, Gross M, et al. Tickborne relapsing fever, Jerusalem, Israel, 2004-2018. *Emerg Infect Dis* 2020; **26**: 2420–23.

28. Grecchi C, Zanotti P, Pontarelli A, et al. Louse-borne relapsing fever in a refugee from Mali. *Infection* 2017; **45**: 373–76.

29. Garnham PC, Davies CW. An epidemic of louse-borne relapsing fever in Kenya. *Trans R Soc Trop Med Hyg* 1947; **41**: 141–70.

30. Forrester JD, Kjemtrup AM, Fritz CL, et al. Tickborne relapsing fever - United States, 1990-2011. *Morb Mortal Wkly Rep* 2015; **64**: 58–60.

31. Dworkin MS, Anderson DE, Jr., Schwan TG, et al. Tick-borne relapsing fever in the northwestern United States and southwestern Canada. *Clin Infect Dis* 1998; **26**: 122–31.

32. Cochez C, Heyman P, Heylen D, et al. The presence of *Borrelia miyamotoi*, a relapsing fever spirochaete, in questing *Ixodes ricinus* in Belgium and in The Netherlands. *Zoonoses Public Health* 2015; **62**: 331–33.

33. Christensen J, Fischer RJ, McCoy BN, Raffel SJ, Schwan TG. Tickborne relapsing fever, Bitterroot Valley, Montana, USA. *Emerg Infect Dis* 2015; **21**: 217–23.

34. Borgnolo G, Hailu B, Ciancarelli A, Almaviva M, Woldemariam T. Louse-borne relapsing fever. a clinical and an epidemiological study of 389 patients in Asella Hospital, Ethiopia. *Trop Geogr Med* 1993; **45**: 66–69.

35. Bodman RI, Stewart IS. Louse-borne relapsing fever in Persia. *Br Med J* 1948; **1**: 291–93.

36. Bissett JD, Ledet S, Krishnavajhala A, et al. Detection of tickborne relapsing fever spirochete, Austin, Texas, USA. *Emerg Infect Dis* 2018; **24**: 2003–09.

37. Barclay AJ, Coulter JB. Tick-borne relapsing fever in central Tanzania. *Trans R Soc Trop Med Hyg* 1990; **84**: 852–56.

38. Ayazi P, Mahyar A, Oveisi S, Esmailzadehha N, Nooroozi S. Tick-borne relapsing fever in children in the north-west of Iran, Qazvin. *Prague Med Rep* 2015; **116**: 193–202.

39. Centers for Disease Control. Outbreak of relapsing fever--Grand Canyon National Park, Arizona, 1990. *Morb Mortal Wkly Rep* 1991; **40**: 296–97.

40. Zammarchi L, Antonelli A, Bartolini L, et al. Louse-borne relapsing fever with meningeal involvement in an immigrant from Somalia to Italy, October 2015. *Vector Borne Zoonotic Dis* 2016; **16**: 352–55.

41. Yparraguirre LA, Machado-Ferreira E, Ullmann AJ, et al. A hard tick relapsing fever group spirochete in a Brazilian *Rhipicephalus* (Boophilus) *microplus*. *Vector Borne Zoonotic Dis* 2007; **7**: 717–21.

42. Yimer M, Mulu W, Ayalew W, Abera B. Louse-borne relapsing fever profile at Felegehiwot referral hospital, Bahir Dar city, Ethiopia: a retrospective study. *BMC Res Notes* 2014; **7**: 250.

43. Yimer M, Abera B, Mulu W, Bezabih B, Mohammed J. Prevalence and risk factors of louse-borne relapsing fever in high risk populations in Bahir Dar city northwest, Ethiopia. *BMC Res Notes* 2014; **7**: 615.

44. Wyplosz B, Mihaila-Amrouche L, Baixench MT, et al. Imported tickborne relapsing fever, France. *Emerg Infect Dis* 2005; **11**: 1801–03.

45. Wroblewski D, Gebhardt L, Prusinski MA, et al. Detection of *Borrelia miyamotoi* and other tick-borne pathogens in human clinical specimens and *Ixodes scapularis* ticks in New York State, 2012-2015. *Ticks Tick Borne Dis* 2017; **8**: 407–11.

46. Wolff BP. Asiatic relapsing fever; report of 134 cases treated with mapharsen. *Ann Intern Med* 1946; **24**: 203–16.

47. Vial L, Durand P, Arnathau C, et al. Molecular divergences of the *Ornithodoros sonrai* soft tick species, a vector of human relapsing fever in West Africa. *Microbes Infect* 2006; **8**: 2605–11.

48. Trevejo RT, Schriefer ME, Gage KL, et al. An interstate outbreak of tick-borne relapsing fever among vacationers at a Rocky Mountain cabin. *Am J Trop Med Hyg* 1998; **58**: 743–47.

49. Trape JF, Duplantier JM, Bouganali H, et al. Tick-borne borreliosis in west Africa. *Lancet* 1991; **337**: 473–75.

50. Takano A, Sugimori C, Fujita H, et al. A novel relapsing fever *Borrelia* sp. infects the salivary glands of the molted hard tick, *Amblyomma geoemydae*. *Ticks Tick Borne Dis* 2012; **3**: 259–61.

51. Perine PL, Teklu B. Antibiotic treatment of louse-borne relapsing fever in Ethiopia: a report of 377 cases. *Am J Trop Med Hyg* 1983; **32**: 1096–100.

52. Spiller GW. Tick-borne relapsing fever due to *Borrelia hermsii* in British Columbia. *CMAJ* 1986; **134**: 46–47.

53. Schwan TG, Policastro PF, Miller Z, et al. Tick-borne relapsing fever caused by *Borrelia hermsii*, Montana. *Emerg Infect Dis* 2003; **9**: 1151–54.

54. Sidi G, Davidovitch N, Balicer RD, et al. Tickborne relapsing fever in Israel. *Emerg Infect Dis* 2005; **11**: 1784–86.

55. Shirani D, Rakhshanpoor A, Cutler SJ, Ghazinezhad B, Naddaf SR. A case of canine borreliosis in Iran caused by *Borrelia persica*. *Ticks Tick Borne Dis* 2016; **7**: 424–26.

56. Schwan TG, Anderson JM, Lopez JE, et al. Endemic foci of the tick-borne relapsing fever spirochete *Borrelia crocidurae* in Mali, West Africa, and the potential for human infection. *PLoS Negl Trop Dis* 2012; **6**: e1924.

57. Sato K, Sakakibara K, Masuzawa T, Ohnishi M, Kawabata H. Case control study: serological evidence that *Borrelia miyamotoi* disease occurs nationwide in Japan. *J Infect Chemother* 2018; **24**: 828–33.

58. Rummens JL, Louwagie A, Van Hoof A, et al. Relapsing fever imported into Belgium: a case report. *Acta Clin Belg* 1987; **42**: 210–14.

59. Rosenthal E. Relapsing fever in Cape Town, a case report. *S Afr Med J,* 1982; **61**: 801–02.

60. Ramos JM, Reyes F, Tesfamariam A, Malmierca E. Louse-borne relapsing fever and malaria co-infection in Ethiopia. *Trop Doct* 2007; **37**: 121–22.

61. Ramos JM, Malmierca E, Reyes F, et al. Characteristics of louse-borne relapsing fever in Ethiopian children and adults. *Ann Trop Med Parasitol* 2004; **98**: 191–96.

62. Ramos JM, Malmierca E, Reyes F, Tesfamariam A. Louse-borne relapsing fever in Ethiopian children: experience of a rural hospital. *Trop Doct* 2009; **39**: 34–36.

63. Platonov AE, Karan LS, Kolyasnikova NM, et al. Humans infected with relapsing fever spirochete *Borrelia miyamotoi*, Russia. *Emerg Infect Dis* 2011; **17**: 1816–23.

64. Perine PL, Parry EH, Vukotich D, Warrell DA, Bryceson AD. Bleeding in louse-borne relapsing fever. I. Clinical studies in 37 patients. *Trans R Soc Trop Med Hyg* 1971; **65**: 776–81.

65. Patrat-Delon S, Drogoul AS, Le Ho H, et al. Recurrent tick-borne fever: a possible diagnosis in patients returning from Senegal. *Med Mal Infect* 2008; **38**: 396–99.

66. Parola P, Ryelandt J, Mangold AJ, et al. Relapsing fever *Borrelia* in *Ornithodoros* ticks from Bolivia. *Ann Trop Med Parasitol* 2011; **105**: 407–11.

67. Page S, Daschkin C, Anniko S, et al. First report of *Borrelia miyamotoi* in an *Ixodes ricinus* tick in Augsburg, Germany. *Exp Appl Acarol* 2018; **74**: 191–99.

68. Naddaf SR, Ghazinezhad B, Sedaghat MM, Asl HM, Cutler SJ. Tickborne relapsing fever in southern Iran, 2011-2013. *Emerg Infect Dis* 2015; **21**: 1078–80.

69. Muñoz-Leal S, Faccini-Martínez Á A, Teixeira BM, et al. Relapsing fever group *borreliae* in human-biting soft ticks, Brazil. *Emerg Infect Dis* 2021; **27**: 322–24.

70. Muigg V, Seth-Smith HMB, Goldenberger D, et al. Tick-borne relapsing fever caused by *Borrelia persica* in traveler to central Asia, 2019. *Emerg Infect Dis* 2020; **26**: 824–26.

71. Moran-Gilad J, Levine H, Schwartz E, et al. Postexposure prophylaxis of tick-borne relapsing fever: lessons learned from recent outbreaks in Israel. *Vector Borne Zoonotic Dis* 2013; **13**: 791–97.

72. Mediannikov O, Socolovschi C, Bassene H, et al. *Borrelia crocidurae* infection in acutely febrile patients, Senegal. *Emerg Infect Dis* 2014; **20**: 1335–38.

73. Cross ST, Kapuscinski ML, Perino J, et al. Co-infection patterns in individual *Ixodes scapularis* ticks reveal associations between viral, eukaryotic and bacterial microorganisms. *Viruses* 2018; **10**: 388.

74. McCall PJ, Hume JC, Motshegwa K, et al. Does tick-borne relapsing fever have an animal reservoir in East Africa? *Vector Borne Zoonotic Dis* 2007; **7**: 659–66.

75. Makwabe CM. Tick borne relapsing fever in Tanzanian children. *Cent Afr J Med* 1984; **30**: 148–50.

76. Mafi N, Yaglom HD, Levy C, et al. Tick-borne relapsing fever in the White Mountains, Arizona, USA, 2013-2018. *Emerg Infect Dis* 2019; **25**: 649–53.

77. Krishnavajhala A, Armstrong BA, Kneubehl AR, et al. Diversity and distribution of the tick-borne relapsing fever spirochete *Borrelia turicatae*. *PLoS Negl Trop Dis* 2021; **15**: e0009868.

78. Lynn GE, Graham CB, Horiuchi K, et al. Prevalence and geographic distribution of *Borrelia miyamotoi* in host-seeking *Ixodes pacificus* (Acari: Ixodidae) nymphs in Mendocino County, California. *J Med Entomol* 2018; **55**: 711–16.

79. Maegli A, Loy JD, Cortinas R. Note on *Ehrlichia chaffeensis, Ehrlichia ewingii,* and "*Borrelia lonestari*" infection in lone star ticks (Acari: Ixodidae), Nebraska, USA. *Ticks Tick Borne Dis* 2016; **7**: 154–58.

80. Kleinerman G, Eshed T, Nachum-Biala Y, King R, Baneth G. Transmission of the human relapsing fever spirochete *Borrelia persica* by the Argasid tick *Ornithodoros tholozani* involves blood meals from wildlife animal reservoirs and mainly transstadial transfer. *Appl Environ Microbiol* 2021; **87**: e03117–20.

81. Kisinza WN, McCall PJ, Mitani H, Talbert A, Fukunaga M. A newly identified tick-borne *Borrelia* species and relapsing fever in Tanzania. *Lancet* 2003; **362**: 1283–84.

82. Kingry LC, Anacker M, Pritt B, et al. Surveillance for and discovery of *Borrelia* Species in US patients suspected of tickborne illness. *Clin Infect Dis* 2018; **66**: 1864–71.

83. Kiewra D, Stańczak J, Richter M. *Ixodes ricinus* ticks (Acari, Ixodidae) as a vector of *Borrelia burgdorferi* sensu lato and *Borrelia miyamotoi* in Lower Silesia, Poland--preliminary study. *Ticks Tick Borne Dis* 2014; **5**: 892–97.

84. Keesing F, McHenry DJ, Hersh MH, Ostfeld RS. Spatial and temporal patterns of the emerging tick-borne pathogen *Borrelia miyamotoi* in blacklegged ticks (*Ixodes scapularis*) in New York. *Parasit Vectors* 2021; **14**: 51.

85. Johnson TL, Fischer RJ, Raffel SJ, Schwan TG. Host associations and genomic diversity of *Borrelia hermsii* in an endemic focus of tick-borne relapsing fever in western North America. *Parasit Vectors* 2016; **9**: 575.

86. Hytönen J, Khawaja T, Grönroos JO, et al. Louse-borne relapsing fever in Finland in two asylum seekers from Somalia. *Apmis* 2017; **125**: 59–62.

87. Hussein H, Showler A, Tan DH. Tick-borne relapsing fever in pregnancy. *CMAJ* 2014; **186**: 131–34.

88. Hoch M, Wieser A, Löscher T, et al. Louse-borne relapsing fever (*Borrelia recurrentis*) diagnosed in 15 refugees from northeast Africa: epidemiology and preventive control measures, Bavaria, Germany, July to October 2015. *Euro Surveill* 2015; **20**: 1560.

89. Gugliotta JL, Goethert HK, Berardi VP, Telford SR. Meningoencephalitis from *Borrelia miyamotoi* in an immunocompromised patient. *N Engl J Med* 2013; **368**: 240–45.

90. Gras E, Bailly E, Le Brun C, Lemaignen A, Lanotte P. *Borrelia crocidurae* tick-borne relapsing fever upon return from Senegal. *Med Mal Infect* 2019; **49**: 624–625.

91. Goldenberger D, Claas GJ, Bloch-Infanger C, et al. Louse-borne relapsing fever (*Borrelia recurrentis*) in an Eritrean refugee arriving in Switzerland, August 2015. *Euro Surveill* 2015; **20**: 2–5.

92. Gill JS, Ullmann AJ, Loftis AD, et al. Novel relapsing fever spirochete in bat tick. *Emerg Infect Dis* 2008; **14**: 522–23.

93. Gao Y, Lv XL, Han SZ, et al. First detection of *Borrelia miyamotoi* infections in ticks and humans from the northeast of Inner Mongolia, China. *Acta Trop* 2021; **217**: 105857.

94. Gaither M, Schumacher M, Nieto N, et al. Where are the ticks? Solving the mystery of a tickborne relapsing fever outbreak at a youth camp. *J Environ Health* 2016; **78**: 8–11.

95. Fry AS. An epidemic of fifty-four cases of relapsing fever observed in Birjand, East Persia. *Ind Med Gaz* 1920; **55**: 2–8.

96. Fonville M, Friesema IH, Hengeveld PD, et al. Human exposure to tickborne relapsing fever spirochete *Borrelia miyamotoi*, the Netherlands. *Emerg Infect Dis* 2014; **20**: 1244–45.

97. Flanigan TP, Schwan TG, Armstrong C, Van Voris LP, Salata RA. Relapsing fever in the US Virgin Islands: a previously unrecognized focus of infection. *J Infect Dis* 1991; **163**: 1391–92.

98. Dunn, L. H. Studies on the South American tick, *Ornithodoros venezuelensis* Brumpt, in Colombia. *J Parasitol* 1927; **13**: 249–55.

99. Eckbo EJ, Charles M, Wolber R, Yu G. Relapsing fever in a traveller returning from Senegal. *CMAJ* 2021; **193**: e285–88.

100. Domínguez MC, Vergara S, Gómez MC, Roldán ME. Epidemiology of tick-borne relapsing fever in endemic area, Spain. *Emerg Infect Dis* 2020; **26**: 849–56.

101. Diatta G, Mediannikov O, Boyer S, et al. An alternative strategy of preventive control of tick-borne relapsing fever in rural areas of Sine-Saloum, Senegal. *Am J Trop Med Hyg* 2016; **95**: 537–45.

102. De Zulueta J, Nasrallah S, Karam JS, et al. Finding of tick-borne relapsing fever in Jordan by the Malaria Eradication Service. *Ann Trop Med Parasitol* 1971; **65**: 491–95.

103. Davis H, Vincent JM, Lynch J. Tick-borne relapsing fever caused by *Borrelia turicatae*. *Pediatr Infect Dis J* 2002; **21**: 703–05.

104. Darcis G, Hayette MP, Bontems S, et al. Louse-borne relapsing fever in a refugee from Somalia arriving in Belgium. *J Travel Med* 2016; **23**: 9.

105. Croche Santander B, Sánchez Carrión A, Campos E, et al. Tick-borne relapsing fever in a rural area of southern Spain. *An Pediatr (Barc)* 2015; **82**: e73–77.

106. Costescu Strachinaru DI, Cambier J, Kandet-Yattara H, Konopnicki D. Relapsing fever in asylum seekers from Somalia arriving in Belgium in August 2015. *Acta Clin Belg* 2016; **71**: 353–55.

107. Colin de Verdiere N, Hamane S, Assous MV, et al. Tickborne relapsing fever caused by *Borrelia persica*, Uzbekistan and Tajikistan. *Emerg Infect Dis* 2011; **17**: 1325–27.

108. Ciervo A, Mancini F, di Bernardo F, et al. Louseborne relapsing fever in young migrants, Sicily, Italy, July-September 2015. *Emerg Infect Dis* 2016; **22**: 152–53.

109. Castilla-Guerra L, Marín-Martín J, Colmenero-Camacho MA. Tick-borne relapsing fever, Southern Spain, 2004-2015. *Emerg Infect Dis* 2016; **22**: 2217–19.

110. Brummitt SI, Kjemtrup AM, Harvey DJ, et al. *Borrelia burgdorferi* and *Borrelia miyamotoi* seroprevalence in California blood donors. *PLoS One* 2020; **15**: e0243950.

111. Brahim H, Perrier-Gros-Claude JD, Postic D, Baranton G, Jambou R. Identifying relapsing fever *Borrelia*, Senegal. *Emerg Infect Dis* 2005; **11**: 474–75.

112. Samuel T. Darling, M.D. The relapsing fever of Panama. *Ind Med Gaz* 1910; **3**: 115–16.

113. Boyer PH, Koetsveld J, Zilliox L, et al. Assessment of *Borrelia miyamotoi* in febrile patients and ticks in Alsace, an endemic area for Lyme borreliosis in France. *Parasit Vectors* 2020; **13**: 199.

114. Boyer KM, Munford RS, Maupin GO, et al. Tick-borne relapsing fever: an interstate outbreak originating at Grand Canyon National Park. *Am J Epidemiol* 1977; **105**: 469–79.

115. Borgnolo G, Denku B, Chiabrera F, Hailu B. Louse-borne relapsing fever in Ethiopian children: a clinical study. *Ann Trop Paediatr* 1993; **13**: 165–71.

116. Bell S. Predisposing factors in tickborne relapsing fever in Meru district, Kenya. *Trans R Soc Trop Med Hyg* 1953; **47**: 309–17.

117. Balicer RD, Mimouni D, Bar-Zeev Y, et al. Post exposure prophylaxis of tick-borne relapsing fever. *Eur J Clin Microbiol Infect Dis* 2010; **29**: 253–58.

118. Badger MS. Tick talk: unusually severe case of tick-borne relapsing fever with acute respiratory distress syndrome--case report and review of the literature. *Wilderness Environ Med* 2008; **19**: 280–86.

119. Aviles ES, Oakes M, Algranati M, Mansoor AM. Tick-borne relapsing fever. *BMJ Case Rep* 2020; **13**: e237296.

120. Antinori S, Mediannikov O, Corbellino M, Raoult D. Louse-borne relapsing fever among East African refugees in Europe. *Travel Med Infect Dis* 2016; **14**: 110–14.

121. Antinori S, Mediannikov O, Corbellino M, et al. Louse-borne relapsing fever (*Borrelia recurrentis*) in a Somali refugee arriving in Italy: A re-emerging infection in Europe? *PLoS Negl Trop Dis* 2016; **10**: e0004522.

122. Ahmed Yahia S, Faibis F, Benmoussa M, et al. Tick-borne relapsing fever: An unrecognized cause of fever in travellers. *Rev Med Interne* 2020; **41**: 418–20.

123. Acute respiratory distress syndrome in persons with tickborne relapsing fever--three states, 2004-2005. *Morb Mortal Wkly Rep* 2007; **56**: 1073–76.

124. Zei, Z. A. Louse borne relapsing fever (LBRF): mortality and frequency of Jarisch-Herxheimer reaction. *J R Soc Health* 1987; **107**: 146–47.

125. Yu S, Modarelli J, Tomeček JM, et al. Prevalence of common tick-borne pathogens in white-tailed deer and coyotes in south Texas. *Int J Parasitol Parasites Wildl* 2020; **11**: 129–35.

126. Yossepowitch O, Gottesman T, Schwartz-Harari O, Soroksky A, Dan M. Aseptic meningitis and adult respiratory distress syndrome caused by *Borrelia persica*. *Infection* 2012; **40**: 695–97.

127. Yang Y, Yang Z, Kelly P, et al. *Borrelia miyamotoi* sensu lato in Père David Deer and *Haemaphysalis longicornis* ticks. *Emerg Infect Dis* 2018; **24**: 928–31.

128. Yamano K, Ito T, Kiyanagi K, et al. Case report: clinical features of a case of suspected *Borrelia miyamotoi* disease in Hokkaido, Japan. *Am J Trop Med Hyg* 2017; **97**: 84–87.

129. Yagupsky P, Moses S. Neonatal *Borrelia* species infection (relapsing fever). *Am J Dis Child* 1985; **139**: 74–76.

130. Xu G, Pearson P, Dykstra E, Andrews ES, Rich SM. Human-biting *Ixodes* ticks and pathogen prevalence from California, Oregon, and Washington. *Vector Borne Zoonotic Dis* 2019; **19**: 106–14.

131. Wynns HL, Beck MD. Epidemiological studies on relapsing fever in California. *Am J Public Health Nations Health,* 1935; **25**: 270–76.

132. Wormser GP, Masters E, Liveris D, et al. Microbiologic evaluation of patients from Missouri with erythema migrans. *Clin Infect Dis* 2005; **40**: 423–28.

133. Wodecka B, Skotarczak B. Identification of host blood-meal sources and *Borrelia* in field-collected *Ixodes ricinus* ticks in north-western Poland. *Ann Agric Environ Med* 2016; **23**: 59–63.

134. Wilhelmsson P, Jaenson TGT, Olsen B, Waldenström J, Lindgren PE. Migratory birds as disseminators of ticks and the tick-borne pathogens *Borrelia* bacteria and tick-borne encephalitis (TBE) virus: a seasonal study at Ottenby Bird Observatory in South-eastern Sweden. *Parasit Vectors* 2020; **13**: 607.

135. Wilhelmsson P, Fryland L, Börjesson S, et al. Prevalence and diversity of *Borrelia* species in ticks that have bitten humans in Sweden. *J Clin Microbiol* 2010; **48**: 4169–76.

136. Walton GA. Relapsing fever in the Meru District of Kenya. *East Afr Med J* 1950; **27**: 94–98.

137. Varela-Stokes AS. Transmission of bacterial agents from lone star ticks to white-tailed deer. *J Med Entomol* 2007; **44**: 478–83.

138. van Duijvendijk G, Coipan C, Wagemakers A, et al. Larvae of *Ixodes ricinus* transmit *Borrelia afzelii* and *B. miyamotoi* to vertebrate hosts. *Parasit Vectors* 2016; **9**: 97.

139. van Dam AP, van Gool T, Wetsteyn JC, Dankert J. Tick-borne relapsing fever imported from West Africa: diagnosis by quantitative buffy coat analysis and in vitro culture of *Borrelia crocidurae*. *J Clin Microbiol* 1999; **37**: 2027–30.

140. Vaculová T, Derdáková M, Špitalská E, et al. Simultaneous occurrence of *Borrelia miyamotoi,* *Borrelia burgdorferi* sensu lato, *Anaplasma phagocytophilum* and *Rickettsia helvetica* in *Ixodes ricinus* ticks in Urban Foci in Bratislava, Slovakia. *Acta Parasitol* 2019; **64**: 19–30.

141. Ullmann AJ, Gabitzsch ES, Schulze TL, Zeidner NS, Piesman J. Three multiplex assays for detection of *Borrelia burgdorferi* sensu lato and *Borrelia miyamotoi* sensu lato in field-collected *Ixodes* nymphs in North America. *J Med Entomol* 2005; **42**: 1057–62.

142. Uhlmann EJ, Seed PC, Schwan TG, Storch GA. Tick-borne relapsing fever polymerase chain reaction of tick-borne relapsing fever caused by *Borrelia hermsii*. *Pediatr Infect Dis J* 2007; **26**: 267–69.

143. Raoult D, Birtles RJ, Montoya M, et al. Survey of three bacterial louse-associated diseases among rural Andean communities in Peru: prevalence of epidemic typhus, trench fever, and relapsing fever. *Clin Infect Dis* 1999; **2**: 434–36.

144. Trape JF, Diatta G, Arnathau C, et al. The epidemiology and geographic distribution of relapsing fever borreliosis in West and North Africa, with a review of the *Ornithodoros erraticus* complex (Acari: Ixodida). *PLoS One* 2013; **8**: e78473.

145. Tobudic S, Burgmann H, Stanek G, et al. Human *Borrelia miyamotoi* infection, Austria. *Emerg Infect Dis* 2020; **26**: 2201–04.

146. Thomas NJ, Bunikis J, Barbour AG, Wolcott MJ. Fatal spirochetosis due to a relapsing fever-like *Borrelia* sp. in a northern spotted owl. *J Wildl Dis* 2002; **38**: 187–193.

147. Taylor KR, Takano A, Konnai, et al. Borrelia miyamotoi infections among wild rodents show age and month independence and correlation with *Ixodes persulcatus* larval attachment in Hokkaido, Japan. *Vector Borne Zoonotic Dis* 2013; **13**: 92–97.

148. Takumi K, Sprong H, Hofmeester TR. Impact of vertebrate communities on *Ixodes ricinus*-borne disease risk in forest areas. *Parasit Vectors* 2019; **12**: 434.

149. Tadin A, Tokarz R, Markotić A, et al. Molecular survey of zoonotic agents in rodents and other small mammals in Croatia. *Am J Trop Med Hyg* 2016; **94**: 466–73.

150. Takhampunya R, Thaloengsok S, Tippayachai B, et al. Retrospective survey of *Borrelia* spp. from rodents and ticks in Thailand. *J Med Entomol* 2021; **58**: 1331–44.

151. Szekeres S, Coipan EC, Rigó K, et al. Eco-epidemiology of *Borrelia miyamotoi* and Lyme borreliosis spirochetes in a popular hunting and recreational forest area in Hungary. *Parasit Vectors* 2015; **8**: 309.

152. Szekeres S, Lügner J, Fingerle V, Margos G, Földvári G Prevalence of *Borrelia miyamotoi* and *Borrelia burgdorferi* sensu lato in questing ticks from a recreational coniferous forest of East Saxony, Germany. *Ticks Tick Borne Dis* 2017; **8**: 922–27.

153. Sytykiewicz H, Karbowiak G, Chorostowska-Wynimko J, et al. Coexistence of *Borrelia burgdorferi* s.l. genospecies within *Ixodes ricinus* ticks from central and eastern Poland. *Acta Parasitol* 2015; **60**: 654–61.

154. Stromdahl EY, Williamson PC, Kollars TM, Jr., et al. Evidence of *Borrelia lonestari* DNA in *Amblyomma americanum* (Acari: Ixodidae) removed from humans. *J Clin Microbiol* 2003; **41**: 5557–62.

155. Stromdahl EY, Nadolny RM, Gibbons JA, et al. *Borrelia burgdorferi* not confirmed in human-biting *Amblyomma americanum* ticks from the southeastern United States. *J Clin Microbiol* 2015; **53**: 1697–704.

156. Stegall-Faulk T, Clark DC, Wright SM. Detection of *Borrelia lonestari* in *Amblyomma americanum* (Acari: Ixodidae) from Tennessee. *J Med Entomol* 2003; **40**: 100–02.

157. Steen R, Townsend RS. Relapsing fever in Bulandshahr District. *Ind Med Gaz* 1913; **48**: 338–41.

158. Sormunen JJ, Penttinen R, Klemola T, et al. Tick-borne bacterial pathogens in southwestern Finland. *Parasit Vectors* 2016; **9**: 168.

159. Sprong H, Fonville M, Docters van Leeuwen A, et al. Detection of pathogens in *Dermacentor reticulatus* in northwestern Europe: evaluation of a high-throughput array. *Heliyon* 2019; **5**: e01270.

160. Sormunen JJ, Klemola T, Hänninen J, et al. The importance of study duration and spatial scale in pathogen detection-evidence from a tick-infested island. *Emerg Microbes Infect* 2018; **7**: 189.

161. Sprong H, Moonen S, van Wieren SE, Hofmeester TR. Effects of cattle grazing on *Ixodes ricinus*-borne disease risk in forest areas of the Netherlands. *Ticks Tick Borne Dis* 2020; **11**: 101355.

162. Sormunen JJ, Andersson T, Aspi J. Monitoring of ticks and tick-borne pathogens through a nationwide research station network in Finland. *Ticks Tick Borne Dis* 2020; **11**: 101449.

163. Sormunen JJ, Klemola T, Vesterinen EJ, et al. Assessing the abundance, seasonal questing activity, and *Borrelia* and tick-borne encephalitis virus (TBEV) prevalence of *Ixodes ricinus* ticks in a Lyme borreliosis endemic area in Southwest Finland. *Ticks Tick Borne Dis* 2016; **7**: 208–15.

164. Springer A, Raulf MK, Fingerle V, Strube C. Borrelia prevalence and species distribution in ticks removed from humans in Germany, 2013-2017. *Ticks Tick Borne Dis* 2020; **11**: 101363.

165. Konitzer L. Relapsing fever in ambulatory practice. *Acta Med Orient* 1949; **8**: 48–53.

166. Palma M, Lopes de Carvalho I, Figueiredo M, et al. *Borrelia hispanica* in *Ornithodoros erraticus*, Portugal. *Clin Microbiol Infect* 2012; **18**: 696–701.

167. Horton JM, Blaser MJ. The spectrum of relapsing fever in the Rocky Mountains. *Arch Intern Med* 1985; **145**: 871–75.

168. Zakham F, Jääskeläinen AJ, Castrén J, et al. Molecular detection and phylogenetic analysis of *Borrelia miyamotoi* strains from ticks collected in the capital region of Finland. *Ticks Tick Borne Dis* 2021; **12**: 101608.

169. Whitney MS, Schwan TG, Sultemeier KB, McDonald PS, Brillhart MN. Spirochetemia caused by *Borrelia turicatae* infection in 3 dogs in Texas. *Vet Clin Pathol* 2007; **36**: 212–16.

170. Warrell DA, Pope HM, Parry EH, Perine PL, Bryceson AD. Cardiorespiratory disturbances associated with infective fever in man: studies of Ethiopian louse-borne relapsing fever. *Clin Sci* 1970; **39**: 123–45.

171. Wagemakers A, Jahfari S, de Wever B, et al. *Borrelia miyamotoi* in vectors and hosts in The Netherlands. *Ticks Tick Borne Dis* 2017; **8**: 370–74.

172. Snavely E, Hymas W, Couturier MR. The Brief Case: Tick-borne relapsing fever in a returned traveler. *J Clin Microbiol* 2020; **58**: e01621–19.

173. Smith RP Jr, Elias SP, Cavanaugh CE, et al. Seroprevalence of *Borrelia burgdorferi*, *B. miyamotoi*, and Powassan Virus in residents bitten by *Ixodes* Ticks, Maine, USA. *Emerg Infect Dis* 2019; **25**: 804–07.

174. Smith L, Brown TG. Brown. Relapsing fever-a case history. *Calif Med* 1969; **110**: 322–24.

175. Shannon AB, Rucinsky R, Gaff HD, Brinkerhoff RJ. *Borrelia miyamotoi*, other vector-borne agents in cat blood and ticks in Eastern Maryland. *Ecohealth* 2017; **14**: 816–20.

176. Sinton JA. Relapsing fever at Meshed, North-East Persia. *Ind Med Gaz* 1921; **56**: 241–50.

177. Seto J, Tanaka S, Kawabata H, et al. Detection of tick-borne pathogens in ticks from dogs and cats in the Yamagata Prefecture of Japan in 2018. *Jpn J Infect Dis* 2021; **74**: 122–28.

178. Scott MC, Rosen ME, Hamer SA, et al. High-prevalence *Borrelia miyamoto*i infection among wild turkeys (*Meleagris gallopavo*) in Tennessee. *J Med Entomol* 2010; **47**: 1238–42.

179. Schreiber C, Krücken J, Beck S, et al. Pathogens in ticks collected from dogs in Berlin/Brandenburg, Germany. *Parasit Vectors* 2014; **7**: 535.

180. Sayler KA, Loftis AD, Beatty SK, et al. Prevalence of tick-borne pathogens in host-seeking *Amblyomma americanum* (Acari: Ixodidae) and *Odocoileus virginianus* (Artiodactyla: Cetartiodactyla) in Florida. *J Med Entomol* 2016; **53**: 949–56.

181. Sato K, Takano A, Konnai S, et al. Human infections with *Borrelia miyamotoi*, Japan. *Emerg Infect Dis* 2014; **20**: 1391–93.

182. Sarih M, Garnier M, Boudebouch N, et al. *Borrelia hispanica* relapsing fever, Morocco. *Emerg Infect Dis* 2009; **15**: 1626–29.

183. Sanseverino L, Ortêncio Filho H, Esteve-Gassent M, Jorge TMR. Test for *Borrelia* spp. in bats in an urban area in the South of Brazil. *Rev Soc Bras Med Trop* 2019; **52**: e20190234.

184. Salkeld DJ, Nieto NC, Carbajales-Dale P, et al. Disease risk & landscape attributes of tick-borne *Borrelia* pathogens in the San Francisco Bay Area, California. *PLoS One* 2015; **10**: e0134812.

185. Salkeld DJ, Nieto NC, Bonilla DL, Yoshimizu MH, Padgett KA. *Borrelia miyamotoi* infections in small mammals, California, USA. *Emerg Infect Dis* 2018; **24**: 2356–59.

186. Truong AT, Noh J, Park Y, et al. Molecular detection and phylogeny of tick-borne pathogens in ticks collected from dogs in the Republic of Korea. *Pathogens* 2021; **10**: 613.

187. Tokarz R, Tagliafierro T, Sameroff S, et al. Microbiome analysis of *Ixodes scapularis* ticks from New York and Connecticut. *Ticks Tick Borne Dis* 2019; **10**: 894–900.

188. Taft WC, Pike JB. Relapsing fever; report of a sporadic outbreak, including treatment with penicillin. *J Am Med Assoc* 1945; **129**: 1002–05.

189. Szekeres S, Docters van Leeuwen A, Tóth E, et al. Road-killed mammals provide insight into tick-borne bacterial pathogen communities within urban habitats. *Transbound Emerg Dis* 2019; **66**: 277–86.

190. Smith MP, Ponnusamy L, Jiang J, et al. Bacterial pathogens in ixodid ticks from a Piedmont County in North Carolina: prevalence of rickettsial organisms. *Vector Borne Zoonotic Dis* 2010; **10**: 939–52.

191. Salkeld DJ, Lagana DM, Wachara J, Porter WT, Nieto NC. Examining prevalence and diversity of tick-borne pathogens in questing *Ixodes pacificus* ticks in California. *Appl Environ Microbiol* 2021; **87**: e0031921.

192. Salkeld DJ, Cinkovich S, Nieto NC. Tick-borne pathogens in northwestern California, USA. *Emerg Infect Dis* 2014; **20**: 493–94.

193. Sakakibara K, Şen E, Sato K, et al. Detection and characterization of the emerging relapsing fever pathogen, *Borrelia miyamotoi*, from the *Ixodes ricinus* tick in the rural Trakya (Thrace) Region of Northwestern Turkey. *Vector Borne Zoonotic Dis* 2016; **16**: 797–99.

194. Rustenhoven-Spaan I, Melkert P, Nelissen E, van Roosmalen J, Stekelenburg J. Maternal mortality in a rural Tanzanian hospital: fatal Jarisch-Herxheimer reaction in a case of relapsing fever in pregnancy. *Trop Doct* 2013; **43**: 138–41.

195. Roux V, Raoult D. Body lice as tools for diagnosis and surveillance of reemerging diseases. *J Clin Microbiol* 1999; **37**: 596–99.

196. Rogers L. Relapsing fever (Sunjar) in the Kumaon Himalayas. *Ind Med Gaz* 1899; **34**: 151–52.

197. Robinson P. Relapsing fever in Addis Ababa. *Br Med J* 1942; **2**: 216–17.

198. Remesar S, Díaz P, Venzal JM, et al. Longitudinal study of infection with *Borrelia* spp. in questing ticks from North-Western Spain. *Vector Borne Zoonotic Dis* 2019; **19**: 785–92.

199. Regier Y, Komma K, Weigel M, et al. Combination of microbiome analysis and serodiagnostics to assess the risk of pathogen transmission by ticks to humans and animals in central Germany. *Parasit Vectors* 2019; **12**: 11.

200. Ravagnan S, Tomassone L, Montarsi F, et al. First detection of *Borrelia miyamotoi* in *Ixodes ricinus* ticks from northern Italy. *Parasit Vectors* 2018; **11**: 130.

201. Răileanu C, Tauchmann O, Vasić A, Wöhnke E, Silaghi C. *Borrelia miyamotoi* and *Borrelia burgdorferi* (sensu lato) identification and survey of tick-borne encephalitis virus in ticks from north-eastern Germany. *Parasit Vectors* 2020; **13**: 106.

202. Qiu Y, Nakao R, Hang'ombe BM, et al. Human borreliosis caused by a new world relapsing fever *Borrelia*-like organism in the old world. *Clin Infect Dis* 2019; **69**: 107–12.

203. Potkonjak A, Kleinerman G, Gutiérrez R, et al. Occurrence of *Borrelia burgdorferi* sensu lato in *Ixodes ricinus* ticks with first identification of *Borrelia miyamotoi* in Vojvodina, Serbia. *Vector Borne Zoonotic Dis* 2016; **16**: 631–35.

204. Porter WT, Motyka PJ, Wachara J, et al. Citizen science informs human-tick exposure in the Northeastern United States. *Int J Health Geogr* 2019; **18**: 9.

205. Piccione J, Levine GJ, Duff CA, et al. Tick-borne relapsing fever in dogs. *J Vet Intern Med* 2016; **30**: 1222–28.

206. Palmer JH, Crawford DJ. Relapsing fever in north America, with report of an outbreak in British Columbia. *Can Med Assoc J* 1933; **28**: 643–47.

207. Pedersen BN, Jenkins A, Kjelland V. Tick-borne pathogens in *Ixodes ricinus* ticks collected from migratory birds in southern Norway. *PLoS One* 2020; **15**: e0230579.

208. Pawełczyk A, Bednarska M, Hamera A, et al. Long-term study of *Borrelia* and *Babesia* prevalence and co-infection in *Ixodes ricinus* and *Dermacentor recticulatus* ticks removed from humans in Poland, 2016-2019. *Parasit Vectors* 2021; **14**: 348.

209. Paul RE, Cote M, Le Naour E, Bonnet SI. Environmental factors influencing tick densities over seven years in a French suburban forest. *Parasit Vectors* 2016; **9**: 309.

210. Smith KA, Oesterle PT, Jardine CM, et al. Tick infestations of wildlife and companion animals in Ontario, Canada, with detection of human pathogens in *Ixodes scapularis* ticks. *Ticks Tick Borne Dis* 2019; **10**: 72–76.

211. Sevestre J, Diarra AZ, Oumarou HA, et al. Detection of emerging tick-borne disease agents in the Alpes-Maritimes region, southeastern France. *Ticks Tick Borne Dis* 2021; **12**: 101800.

212. Seboxa T, Rahlenbeck SI. Treatment of louse-borne relapsing fever with low dose penicillin or tetracycline: a clinical trial. *Scand J Infect Dis* 1995; **27**: 29–31.

213. Schulze TL, Jordan RA, White JC, Roegner VE, Healy SP. Geographical distribution and prevalence of selected *Borrelia, Ehrlichia*, and *Rickettsia* infections in *Amblyomma americanum* (Acari: Ixodidae) in New Jersey. *J Am Mosq Control Assoc* 2011; **27**: 236–44.

214. Pakanen VM, Sormunen JJ, Sippola E, Blomqvist D, Kallio ER. Questing abundance of adult taiga ticks *Ixodes persulcatus* and their *Borrelia* prevalence at the north-western part of their distribution. *Parasit Vectors* 2020; **13**: 384.

215. Osthoff M, Schibli A, Fadini D, Lardelli P, Goldenberger D. Louse-borne relapsing fever - report of four cases in Switzerland, June-December 2015. *BMC Infect Dis* 2016; **16**: 210.

216. Osborne CJ, Crosbie PR, Van Laar TA. *Borrelia parkeri* in *Ornithodoros parkeri* (Ixodida: Argasidae) collected using compact dry ice traps in Madera County, California. *J Med Entomol* 2019; **56**: 579–83.

217. Oechslin CP, Heutschi D, Lenz N, et al. Prevalence of tick-borne pathogens in questing *Ixodes ricinus* ticks in urban and suburban areas of Switzerland. *Parasit Vectors* 2017; **10**: 558.

218. Nordmann T, Feldt T, Bosselmann M, et al. Outbreak of louse-borne relapsing fever among urban dwellers in Arsi Zone, Central Ethiopia, from July to November 2016. *Am J Trop Med Hyg* 2018; **98**: 1599–602.

219. Noden BH, Roselli MA, Loss SR. Effect of urbanization on presence, abundance, and coinfection of bacteria and protozoa in ticks in the US Great Plains. *J Med Entomol* 2022; **3**: 957–68.

220. Nitzan O, Blum A, Marva E, et al. Case report: Infectious diseases in pilgrims visiting the Holy Land. *Am J Trop Med Hyg* 2017; **97**: 611–14.

221. Nieto NC, Teglas MB, Stewart KM, Wasley T, Wolff PL. Detection of relapsing fever spirochetes (*Borrelia hermsii* and *Borrelia coriaceae*) in free-ranging mule deer (*Odocoileus hemionus*) from Nevada, United States. *Vector Borne Zoonotic Dis* 2012; **12**: 99–105.

222. Nieto NC, Teglas MB. Relapsing fever group *Borrelia* in Southern California rodents. *J Med Entomol* 2014; **51**: 1029–34.

223. Nieto NC, Porter WT, Wachara JC, et al. Using citizen science to describe the prevalence and distribution of tick bite and exposure to tick-borne diseases in the United States. *PLoS One* 2018; **13**: e0199644.

224. Nelder MP, Russell CB, Dibernardo A, et al. Monitoring the patterns of submission and presence of tick-borne pathogens in *Ixodes scapularis* collected from humans and companion animals in Ontario, Canada (2011-2017). *Parasit Vectors* 2021; **14**: 260.

225. Sanchez-Vicente S, Tagliafierro T, Coleman JL, Benach JL, Tokarz R. Polymicrobial nature of tick-borne diseases. *mBio* 2019; **10**: e02055–19.

226. Salih SY, Mustafa D. Mustafa. Louse-borne relapsing fever: II. Combined penicillin and tetracycline therapy in 160 Sudanese patients. *Trans R Soc Trop Med Hyg* 1977; **71**: 49–51.

227. Rogovskyy A, Batool M, Gillis DC, et al. Diversity of *Borrelia* spirochetes and other zoonotic agents in ticks from Kyiv, Ukraine. *Ticks Tick Borne Dis* 2018; **9**: 404–09.

228. Rar V, Livanova N, Sabitova Y, et al. *Ixodes persulcatus/pavlovskyi* natural hybrids in Siberia: Occurrence in sympatric areas and infection by a wide range of tick-transmitted agents. *Ticks Tick Borne Dis* 2019; **10**: 101254.

229. Quarsten H, Salte T, Lorentzen Å R, et al. Tick-borne pathogens detected in the blood of immunosuppressed norwegian patients living in a tick-endemic area. *Clin Infect Dis* 2021; **73**: e2364–71.

230. Newcomb C. On an outbreak of relapsing fever in Turkey in 1918. *Ind Med Gaz* 1920; **55**: 208–17.

231. Nazari M, Najafi A. Epidemiological study of endemic relapsing fever in Hamadan Province, West of Iran. *J Arthropod Borne Dis* 2016; **10**: 586–94.

232. Narain S, Kalra SL. Streptomycin in tick-borne relapsing fever of Kashmir. *Ind Med Gaz* 1950; **85**: 87–88.

233. Namina A, Capligina V, Seleznova M, et al. Tick-borne pathogens in ticks collected from dogs, Latvia, 2011-2016. *BMC Vet Res* 2019; **15**: 398.

234. Naddaf SR, Mahmoudi A, Ghasemi A, et al. Infection of hard ticks in the Caspian Sea littoral of Iran with Lyme borreliosis and relapsing fever borreliae. *Ticks Tick Borne Dis* 2020; **11**: 101500.

235. Murdock JH, Yabsley MJ, Little SE, et al. Distribution of antibodies reactive to *Borrelia lonestari* and *Borrelia burgdorferi* in white-tailed deer (*Odocoileus virginianus*) populations in the eastern United States. *Vector Borne Zoonotic Dis* 2009; **9**: 729–36.

236. Mun J, Eisen RJ, Eisen L, Lane RS. Detection of a *Borrelia miyamotoi* sensu lato relapsing-fever group spirochete from *Ixodes pacificus* in California. *J Med Entomol* 2006; **43**: 120–23.

237. Moore VAt, Varela AS, Yabsley MJ, Davidson WR, Little SE. Detection of *Borrelia lonestari*, putative agent of southern tick-associated rash illness, in white-tailed deer (*Odocoileus virginianus*) from the southeastern United States. *J Clin Microbiol* 2003; **41**: 424–27.

238. Mitiku K, Mengistu G. Mengistu. Relapsing fever in Gondar, Ethiopia. *East Afr Med J* 2002; **79**: 85–87.

239. Mitani H, Talbert A, Fukunaga M. New World relapsing fever *Borrelia* found in *Ornithodoros porcinus* ticks in central Tanzania. *Microbiol Immunol* 2004; **48**: 501–05.

240. Milholland MT, Xu G, Rich SM, et al. Pathogen coinfections harbored by adult *Ixodes scapularis* from white-tailed deer compared with questing adults across sites in Maryland, USA. *Vector Borne Zoonotic Dis* 2021; **21**: 86–91.

241. Michelet L, Delannoy S, Devillers E, et al. High-throughput screening of tick-borne pathogens in Europe. *Front Cell Infect Microbiol* 2014; **4**: 103.

242. Mehta BN. Notes on relapsing fever in Bhavnagar. *Ind Med Gaz* 1922; **57**: 456–57.

243. Markowicz M, Schötta AM, Höss D, et al. Infections with tickborne pathogens after tick bite, Austria, 2015-2018. *Emerg Infect Dis* 2021; **27**: 1048–56.

244. Marcos LA, Smith K, Reardon K, Weinbaum F, Spitzer ED. Presence of *Borrelia miyamotoi* infection in a highly endemic area of Lyme disease. *Ann Clin Microbiol Antimicrob* 2020; **19**: 22.

245. Mallannah S. Relapsing fever in Raichur. *Ind Med Gaz* 1923; **58**: 168.

246. Mahram M, Ghavami MB. Congenital tick-borne relapsing fever: report of a case with transplacental transmission in the Islamic Republic of Iran. *East Mediterr Health J* 2009; **15**: 761–64.

247. Mackie FP. The part played by pediculus corporis in the transmission of relapsing fever. *Br Med J* 1907; **2**: 1706–09.

248. Ly TDA, Louni M, Hoang VT, et al. Epidemiological serosurvey of vector-borne and zoonotic pathogens among homeless people living in shelters in Marseille: cross-sectional one-day surveys (2005-2015). *Eur J Clin Microbiol Infect Dis* 2020; **39**: 1663–72.

249. Livengood J, Hutchinson ML, Thirumalapura N, Tewari D. Detection of *Babesia, Borrelia, Anaplasma*, and *Rickettsia* spp. in adult black-legged ticks (*Ixodes scapularis*) from Pennsylvania, United States, with a luminex multiplex bead assay. *Vector Borne Zoonotic Dis* 2020; **20**: 406–11.

250. Liles WC, Spach DH. Late relapse of tick-borne relapsing fever following treatment with doxycycline. *West J Med* 1993; **158**: 200.

251. Lernout T, De Regge N, Tersago K, et al. Prevalence of pathogens in ticks collected from humans through citizen science in Belgium. *Parasit Vectors* 2019; **12**: 550.

252. Lepage P, Ntahorutaba M, Bogaerts J. Neonatal relapsing fever in Rwanda. *Am J Dis Child* 1986; **140**: 89.

253. Lejal E, Moutailler S, Šimo L, Vayssier-Taussat M, Pollet T. Tick-borne pathogen detection in midgut and salivary glands of adult *Ixodes ricinus*. *Parasit Vectors* 2019; **12**: 152.

254. Legge RT. Relapsing Fever: a new etiological observation: with case report of a field worker. *Cal West Med* 1933; **38**: 370–71.

255. Lee SH, Vigliotti JS, Vigliotti VS, et al. DNA sequencing diagnosis of off-season spirochetemia with low bacterial density in *Borrelia burgdorferi* and *Borrelia miyamotoi* infections. *Int J Mol Sci* 2014; **15**: 11364–86.

256. Seo JW, Han SY, Sung SH, et al. Survey on tick distribution and tick-borne pathogens in Daejeon and adjacent areas in South Korea. *Ticks Tick Borne Dis* 2021; **12:** 101711.

257. Laaksonen M, Sajanti E, Sormunen JJ, et al. Crowdsourcing-based nationwide tick collection reveals the distribution of *Ixodes ricinus* and *I. persulcatus* and associated pathogens in Finland. *Emerg Microbes Infect* 2017; **6**: e31.

258. Kutsuna S, Kawabata H, Shiga N, et al. Second Japanese case of relapsing fever. *Kansenshogaku Zasshi* 2014; **88**: 713–14.

259. Kumagai Y, Sato K, Taylor KR, et al. A relapsing fever group *Borrelia* sp. is widely distributed among wild deer in Japan. *Ticks Tick Borne Dis* 2018; **9**: 465–70.

260. Franck M, Ghozzi R, Pajaud J, et al. *Borrelia miyamotoi*: 43 cases diagnosed in France by real-time PCR in patients with persistent polymorphic signs and symptoms. *Front Med (Lausanne)* 2020; **7**: 55.

261. Krause PJ, Narasimhan S, Wormser GP, et al. Human *Borrelia miyamotoi* infection in the United States. *N Engl J Med* 2013; **368**: 291–93.

262. Krause PJ, Schwab J, Narasimhan S, et al. Hard tick relapsing fever caused by *Borrelia miyamotoi* in a child. *Pediatr Infect Dis J* 2016; **35**: 1352–54.

263. Krause PJ, Narasimhan S, Wormser GP, et al. *Borrelia miyamotoi* sensu lato seroreactivity and seroprevalence in the northeastern United States. *Emerg Infect Dis* 2014; **20**: 1183–90.

264. Konkova-Reidman AB, Barsukova DN, Bondarenko EI, Shvalov AN, Lucinina SV. Clinical and epidemiological features of infections ecologically related to ticks in the Chelyabinsk region. *Epidemiol Infect Dis* 2019; **24**: 178–87.

265. Perine PL, Krause DW, Awoke S, McDade JE. Single-dose doxycycline treatment of louse-borne relapsing fever and epidemic typhus. *Lancet* 1974; **2**: 742–44.

266. Leonova GN, Bondarenko EI, Ivanis VA, Belikov SI, Lubova VA. The first cases of *Borrelia miyamotoi* disease in the Far East of Russia. *Epidemiol Infect Dis* 2017; **3**: 57–64.

267. Ouchene N, Nebbak A, Ouchene-Khelifi NA, et al. Molecular detection of avian spirochete *Borrelia anserina* in *Argas persicus* ticks in Algeria. *Comp Immunol Microbiol Infect Dis* 2020; **68**: 101408.

268. Olchovsky D, Pines A, Sadeh M, Kaplinsky N, Frankl O. Multifocal neuropathy and vocal cord paralysis in relapsing fever. *Eur Neurol* 1982; **21**: 340–42.

269. Oda R, Kutsuna S, Sekikawa Y, et al. The first case of imported *Borrelia miyamotoi* disease concurrent with Lyme disease. *J Infect Chemother* 2017; **23**: 333–35.

270. Nebbak A, Dahmana H, Almeras L, et al. Co-infection of bacteria and protozoan parasites in *Ixodes ricinus* nymphs collected in the Alsace region, France. *Ticks Tick Borne Dis* 2019; **10**: 101241.

271. Nassif X, Dupont B, Fleury J, Lapresle C. Ceftriaxone in relapsing fever. *Lancet* 1988; **2**: 394.

272. Muñoz-Leal S, Faccini-Martínez Á A, Costa FB, et al. Isolation and molecular characterization of a relapsing fever *Borrelia* recovered from *Ornithodoros rudis* in Brazil. *Ticks Tick Borne Dis* 2018; **9**: 864–71.

273. Mukhacheva TA, Salikhova, II, Kovalev SY. Multilocus spacer analysis revealed highly homogeneous genetic background of Asian type of *Borrelia miyamotoi*. *Infect Genet Evol* 2015; **31**: 257–62.

274. Million M, Cazorla C, Doudier B, et al. Molecular identification of *Borrelia crocidurae* in a patient returning from Senegal. *BMJ Case Rep* 2009; **2009**: 298.

275. Michalski MM, Kubiak K, Szczotko M, Dmitryjuk M. Tick-borne pathogens in ticks collected from wild ungulates in North-Eastern Poland. *Pathogens* 2021; **10**: 587.

276. Melkert PW. Relapsing fever in pregnancy: analysis of high-risk factors. *Br J Obstet Gynaecol* 1988; **95**: 1070–72.

278. Mayegga E, Ljøstad U, Mygland A, Monstad P. Absence of focal neurological involvement in tick-borne relapsing fever in northern Tanzania. *Eur J Neurol* 2005; **12**: 449–52.

279. Masuzawa T, Sakakibara K, Suzuki K, Sato H, Yasuda S. Detection of Asian-type *Borrelia miyamotoi* from *Ixodes ricinus* inhabiting Tver Province (Russia): a sympatric region for *I. ricinus* and *Ixodes persulcatus*. *Vector Borne Zoonotic Dis* 2020; **20**: 921–23.

280. Malison MD. Relapsing fever. *JAMA* 1979; **241**: 2819–20.

281. Malincarne L, Schiaroli E, Ciervo A, et al. Meningitis with cranial polyneuritis and cavernous sinus thrombosis by *Borrelia crocidurae*: first autochthonous case in Europe. *Int J Infect Dis* 2019; **82**: 30–32.

282. Luu L, Palomar AM, Farrington G, et al. Bacterial pathogens and symbionts harboured by *Ixodes ricinus* ticks parasitising red squirrels in the United Kingdom. *Pathogens* 2021; **10**: 458.

283. Kubiak K, Dziekońska-Rynko J, Szymańska H, et al. Questing *Ixodes ricinus* ticks (Acari, Ixodidae) as a vector of *Borrelia burgdorferi* sensu lato and *Borrelia miyamotoi* in an urban area of north-eastern Poland. *Exp Appl Acarol* 2019; **78**: 113–26.

284. Kowalec M, Szewczyk T, Welc-Falęciak R, et al. Ticks and the city - are there any differences between city parks and natural forests in terms of tick abundance and prevalence of spirochaetes? *Parasit Vectors* 2017; **10**: 573.

285. Koton Y, Bisharat N. Tick-borne relapsing fever with severe Jarisch-Herxheimer reaction. *Isr Med Assoc J* 2018; **20**: 62–63.

286. Kotlyar S. Tick-borne relapsing fever in Southwest Colorado: a case report. *J Emerg Med.* 2017; **52**: 83–85.

287. Lee K, Takano A, Taylor K, et al. A relapsing fever group *Borrelia* sp. similar to *Borrelia lonestari* found among wild sika deer (*Cervus nippon yesoensis*) and *Haemaphysalis* spp. ticks in Hokkaido, Japan. *Ticks Tick Borne Dis* 2014; **5**: 841–47.

288. Kisinza WN, Talbert A, Mutalemwa P, McCall PJ. Community knowledge, attitudes and practices related to tick-borne relapsing fever in Dodoma rural district, central Tanzania. *Tanzan J Health Res* 2008; **10**: 131–36.

289. Kim CM, Seo JW, Kim DM, et al. Detection of *Borrelia miyamotoi* in *Ixodes nipponensis* in Korea. *PLoS One* 2019; **14**: e0220465.

290. Killmaster LF, Loftis AD, Zemtsova GE, Levin ML. Detection of bacterial agents in *Amblyomma americanum* (Acari: Ixodidae) from Georgia, USA, and the use of a multiplex assay to differentiate *Ehrlichia chaffeensis* and *Ehrlichia ewingii*. *J Med Entomol* 2014; **51**: 868–72.

291. Khoo JJ, Lim FS, Tan KK, et al. Detection in Malaysia of a *Borrelia* sp. from *Haemaphysalis hystricis* (Ixodida: Ixodidae). *J Med Entomol* 2017; **54**: 1444–48.

292. Khalil GM, Helmy N, Hoogstraal H, el-Said A. Seasonal dynamics of *Ornithodoros* (Pavlovskyella) *erraticus* (Acari: Ixodoidea: Argasidae) and the spirochete *Borrelia crocidurae* in Egypt. *J Med Entomol* 1984; **21**: 536–39.

293. Kelly AL, Raffel SJ, Fischer RJ, et al. First isolation of the relapsing fever spirochete, *Borrelia hermsii*, from a domestic dog. *Ticks Tick Borne Dis* 2014; **5**: 95–99.

294. Kaul S. Relapsing fever-tick-borne. Account of an outbreak in J.&K. Force, India. *Ind Med Gaz* 1949; **84**: 433–40.

295. Kalmár Z, Sprong H, Mihalca AD, et al. *Borrelia miyamotoi* and *Candidatus Neoehrlichia mikurensis* in *Ixodes ricinus* ticks, Romania. *Emerg Infect Dis* 2016; **22**: 550–51.

296. Kalmár Z, Sándor AD, Matei IA, et al. *Borrelia* spp. in small mammals in Romania. *Parasit Vectors* 2019; **12**: 461.

297. Kadkhoda K, Dumouchel C, Brancato J, Gretchen A, Krause PJ. Human seroprevalence of *Borrelia miyamotoi* in Manitoba, Canada, in 2011-2014: a cross-sectional study. *CMAJ Open* 2017; **5**: 690–93.

298. Jongen VH, van Roosmalen J, Tiems J, Van Holten J, Wetsteyn JC. Tick-borne relapsing fever and pregnancy outcome in rural Tanzania. *Acta Obstet Gynecol Scand* 1997; **76**: 834–38.

299. Jones JM, Hranac CR, Schumacher M, et al. Tick-borne relapsing fever outbreak among a high school football team at an outdoor education camping trip, Arizona, 2014. *Am J Trop Med Hyg* 2016; **95**: 546–50.

300. Johnson TL, Graham CB, Boegler KA, et al. Prevalence and diversity of tick-borne pathogens in nymphal *Ixodes scapularis* (Acari: Ixodidae) in Eastern National Parks. *J Med Entomol* 2017; **54**: 742–51.

301. Jobe DA, Lovrich SD, Oldenburg DG, Kowalski TJ, Callister SM. *Borrelia miyamotoi* Infection in patients from Upper Midwestern United States, 2014-2015. *Emerg Infect Dis* 2016; **22**: 1471–73.

302. Jiang BG, Jia N, Jiang JF, et al. *Borrelia miyamotoi* infections in humans and ticks, Northeastern China. *Emerg Infect Dis* 2018; **24**: 236–41.

303. Jernigan DA, Hart MC, Dodd KK, Jameson S, Farney T. Induced native phage therapy for the treatment of Lyme disease and relapsing fever: a retrospective review of first 14 months in one clinic. *Cureus* 2021; **13**: e20014.

304. James AM, Liveris D, Wormser GP, et al. *Borrelia lonestari* infection after a bite by an *Amblyomma americanum* tick. *J Infect Dis* 2001; **183**: 1810–14.

305. Jahfari S, Ruyts SC, Frazer-Mendelewska E, et al. Melting pot of tick-borne zoonoses: the European hedgehog contributes to the maintenance of various tick-borne diseases in natural cycles urban and suburban areas. *Parasit Vectors* 2017; **10**: 134.

306. Jahfari S, Hofhuis A, Fonville M, et al. Molecular detection of tick-borne pathogens in humans with tick bites and erythema migrans, in the Netherlands. *PLoS Negl Trop Dis* 2016; **10**: e0005042.

307. Ilsley ML. Relapsing fever probably caused by *Borrelia duttonii*. *Calif Med* 1952; **77**: 195–96.

308. Humphry AD. Relapsing Fever in Darjeeling District. *Ind Med Gaz* 1913; **48**: 451.

309. Hudman DA, Sargentini NJ. Prevalence of tick-borne pathogens in Northeast Missouri. *Mo Med* 2018; **115**: 162–68.

310. Hovius JW, de Wever B, Sohne M, et al. A case of meningoencephalitis by the relapsing fever spirochaete *Borrelia miyamotoi* in Europe. *Lancet* 2013; **382**: 658.

311. Subramanian G, Sekeyova Z, Raoult D, Mediannikov O. Multiple tick-associated bacteria in *Ixodes ricinus* from Slovakia. *Ticks and Tick-Borne Dis* 2012; **3**: 405–09.

312. Trape JF, Godeluck B, Diatta G, et al. The spread of tick-borne borreliosis in West Africa and its relationship to sub-Saharan drought. *Am J Trop Med Hyg* 1996; **54**: 289–93.

313. Geller J, Nazarova L, Katargina O, et al. Detection and genetic characterization of relapsing fever spirochete *Borrelia miyamotoi* in Estonian ticks. *PLoS One* 2012; **7**: e51914.

314. Machtinger ET, Nadolny RM, Vinyard BT, et al. Spatial heterogeneity of sympatric tick species and tick-borne pathogens emphasizes the need for surveillance for effective tick control. *Vector Borne Zoonotic Dis* 2021; **21**: 843–53.

315. Lommano E, Dvořák C, Vallotton L, Jenni L, Gern L. Tick-borne pathogens in ticks collected from breeding and migratory birds in Switzerland. *Ticks Tick Borne Dis* 2014; **5**: 871–82.

316. Lim LL, Rosenbaum JT Rosenbaum. *Borrelia hermsii* causing relapsing fever and uveitis. *Am J Ophthalmol* 2006; **142**: 348–49.

317. Lehane A, Maes SE, Graham CB, et al. Prevalence of single and coinfections of human pathogens in *Ixodes* ticks from five geographical regions in the United States, 2013-2019. *Ticks Tick Borne Dis* 2021; **12**: 101637.

318. Layzell SJ, Bailey D, Peacey M, Nuttall PA. Prevalence of *Borrelia burgdorferi* and *Borrelia miyamotoi* in questing *Ixodes* *ricinus* ticks from four sites in the UK. *Ticks Tick Borne Dis* 2018; **9**: 217–24.

319. Latas P, Auckland LD, Teel PD, Hamer SA. *Argas* (persicargas) *giganteus* soft tick infection with *Rickettsia hoogstraali* and relapsing fever *Borrelia* on wild avian species of the desert southwest, USA. *J Wildl Dis* 2020; **56**: 113–25.

320. Lange WR, Schwan TG, Frame JD. Can protracted relapsing fever resemble Lyme disease? *Med Hypotheses* 1991; **35**: 77–79.

321. Lane RS, Fedorova N, Kleinjan JE, Maxwell M. Eco-epidemiological factors contributing to the low risk of human exposure to ixodid tick-borne borreliae in southern California, USA. *Ticks Tick Borne Dis* 2013; **4**: 377–85.

322. Lam JC, Larios OE, Parkins MD, Vaughan SD. A case of tick-borne relapsing fever in pregnancy. *Can Commun Dis Rep* 2020; **46**: 362–64.

323. Kohn M, Krücken J, McKay-Demeler J, et al. Dermacentor reticulatus in Berlin/Brandenburg (Germany): Activity patterns and associated pathogens. *Ticks Tick Borne Dis* 2019; **10**: 191–206.

324. Knoll S, Springer A, Hauck D, et al. Distribution of *Borrelia burgdorferi* s. l. and *Borrelia miyamotoi* in *Ixodes* tick populations in Northern Germany, co-infections with Rickettsiales and assessment of potential influencing factors. *Med Vet Entomol* 2021; **35**: 595–06.

325. Kniazeva V, Pogotskaya Y, Higgs S, Krasko A. The prevalence of different human pathogenic microorganisms transmitted by *Ixodes* tick vectors in Belarus. *Vector Borne Zoonotic Dis* 2021; **21**: 6–10.

326. Knaack RH, Wright LJ, Leithead CS, Kidan TG, Plorde JJ. Penicillin vs. tetracycline in the treatment of louse-borne relapsing fever. a preliminary report. *Ethiop Med J* 1972; **10**: 15–22.

327. Hoornstra D, Koetsveld J, Sprong H, Platonov AE, Hovius JW. *Borrelia miyamotoi* disease in an immunocompetent patient, Western Europe. *Emerg Infect Dis* 2018; **24**: 1770–72.

328. Gaowa, Wulantuya, Sato K, et al. Surveillance of *Borrelia miyamotoi*-carrying ticks and genomic analysis of isolates in Inner Mongolia, China. *Parasit Vectors* 2021; **1**: 368.

329. Hoekstra KA, Kelly MT. Elevated troponin and Jarisch-Herxheimer reaction in tick-borne relapsing fever: a case report. *Case Rep Infect Dis* 2011; **2011**: 950314.

330. Heylen D, Fonville M, Docters van Leeuwen A, et al. Pathogen communities of songbird-derived ticks in Europe's low countries. *Parasit Vectors* 2017; **10**: 497.

331. Heron D. Relapsing fever in Seistan. *Ind Med Gaz* 1917; **52**: 378.

332. Henningsson AJ, Asgeirsson H, Hammas B, et al. Two cases of *Borrelia miyamotoi* meningitis, Sweden, 2018. *Emerg Infect Dis* 2019; **25**: 1965–68.

333. Hayashi T, Miura Y, Kawabata H. *Borrelia miyamotoi* disease rash. *Intern Med* 2018; **57**: 2601–02.

334. Hansford KM, Fonville M, Jahfari S, Sprong H, Medlock JM. *Borrelia miyamotoi* in host-seeking *Ixodes ricinus* ticks in England. *Epidemiol Infect* 2015; **143**: 1079–87.

335. Han S, Lubelczyk C, Hickling GJ, et al. Vertical transmission rates of *Borrelia miyamotoi* in *Ixodes scapularis* collected from white-tailed deer. *Ticks Tick Borne Dis* 2019; **10**: 682–89.

336. Han S, Hickling GJ, Tsao JI. High Prevalence of *Borrelia miyamotoi* among adult blacklegged ticks from white-tailed deer. *Emerg Infect Dis* 2016; **22**: 316–18.

337. Han HJ, Liu JW, Wen HL, et al. Pathogenic new world relapsing fever *Borrelia* in a *Myotis* bat, Eastern China, 2015. *Emerg Infect Dis* 2020; **26**: 3083–85.

338. Hamšíková Z, Coipan C, Mahríková L, et al. *Borrelia miyamotoi* and co-infection with *Borrelia afzelii* in *Ixodes ricinus* ticks and rodents from Slovakia. *Microb Ecol* 2017; **73**: 1000–08.

339. Kumsa B, Socolovschi C, Raoult D, Parola P. New *Borrelia* species detected in ixodid ticks in Oromia, Ethiopia. *Ticks Tick Borne Dis* 2015; **6**: 401–07.

340. Klitgaard K, Højgaard J, Isbrand A, et al. Screening for multiple tick-borne pathogens in *Ixodes ricinus* ticks from birds in Denmark during spring and autumn migration seasons. *Ticks Tick Borne Dis* 2019; **10**: 546–52.

341. Kleinerman G, King R, Nachum-Biala Y, Baneth G. *Borrelia persica* infection in rock hyraxes. *Ticks Tick Borne Dis* 2018; **9**: 382–88.

342. Klein M. Relapsing fever--successful treatment with demethylchlortetracycline (demclomycin). *Calif Med* 1964; **100**: 283–85.

343. Kjelland V, Rollum R, Korslund L, Slettan A, Tveitnes D. *Borrelia miyamotoi* is widespread in *Ixodes ricinus* ticks in southern Norway. *Ticks Tick Borne Dis* 2015; **6**: 516–21.

344. Khasnatinov MA, Danchinova GA, Takano A, et al. Prevalence of *Borrelia miyamotoi* in *Ixodes persulcatus* in Irkutsk City and its neighboring territories, Russia. *Ticks Tick Borne Dis* 2016; **7**: 394–97.

345. Kjelland V, Paulsen KM, Rollum R, et al. Tick-borne encephalitis virus, *Borrelia burgdorferi* sensu lato, *Borrelia miyamotoi*, *Anaplasma phagocytophilum* and *Candidatus Neoehrlichia mikurensis* in *Ixodes ricinus* ticks collected from recreational islands in southern Norway. *Ticks Tick Borne Dis* 2018; **9**: 1098–1102.

346. Iwabu-Itoh Y, Bazartseren B, Naranbaatar O, et al. Tick surveillance for *Borrelia miyamotoi* and phylogenetic analysis of isolates in Mongolia and Japan. *Ticks Tick Borne Dis* 2017; **8**: 850–57.

347. Allan BF, Goessling LS, Storch GA, Thach RE. Blood meal analysis to identify reservoir hosts for *Amblyomma americanum* ticks. *Emerg Infect Dis* 2010; **16**: 433–40.

348. Hudman DA, Sargentini NJ. Detection of *Borrelia, Ehrlichia*, and *Rickettsia* spp. in ticks in northeast Missouri. *Ticks Tick Borne Dis* 2016; **7**: 915–21.

349. Hornok S, Daccord J, Takács N, et al. Investigation on haplotypes of ixodid ticks and retrospective finding of *Borrelia miyamotoi* in bank vole (*Myodes glareolus*) in Switzerland. *Ticks Tick Borne Dis* 2022; **13**: 101865.

350. Hansford KM, Wheeler BW, Tshirren B, Medlock JM. Urban woodland habitat is important for tick presence and density in a city in England. *Ticks Tick Borne Dis* 2022; **13**: 101857.

351. Han SW, Chae JB, Jo YS, et al. First detection of *Borrelia* and *Rickettsia* species from *Ornithodoros* ticks in the Republic of Korea. *Ticks Tick Borne Dis* 2021; **12**: 101689.

352. Han S, Hickling GJ, Ogden NH, et al. Seasonality of acarological risk of exposure to *Borrelia miyamotoi* from questing life stages of *Ixodes scapularis* collected from Wisconsin and Massachusetts, USA. *Ticks Tick Borne Dis* 2021; **12**: 101556.

353. Hamer SA, Hickling GJ, Walker ED, Tsao JI. Increased diversity of zoonotic pathogens and *Borrelia burgdorferi* strains in established versus incipient *Ixodes scapularis* populations across the Midwestern United States. *Infect Genet Evol* 2014; **27**: 531–42.

354. Hamer SA, Hickling GJ, Keith R, et al. Associations of passerine birds, rabbits, and ticks with *Borrelia miyamotoi* and *Borrelia andersonii* in Michigan, U.S.A. *Parasit Vectors* 2012; **5**: 231.

355. Halperin T, Orr N, Cohen R, et al. Detection of relapsing fever in human blood samples from Israel using PCR targeting the glycerophosphodiester phosphodiesterase (GlpQ) gene. *Acta Trop* 2006; **98**: 189–95.

356. Hall JL, Alpers K, Bown KJ, Martin SJ, Birtles RJ. Use of mass-participation outdoor events to assess human exposure to tickborne pathogens. *Emerg Infect Dis* 2017; **23**: 463–67.

357. Hahn MB, Bjork JKH, Neitzel DF, et al. Evaluating acarological risk for exposure to *Ixodes scapularis* and *Ixodes scapularis*-borne pathogens in recreational and residential settings in Washington County, Minnesota. *Ticks Tick Borne Dis* 2018; **9**: 340–48.

358. Gyllemark P, Wilhelmsson P, Elm C, et al. Are other tick-borne infections overlooked in patients investigated for Lyme neuroborreliosis? A large retrospective study from South-Eastern Sweden. *Ticks Tick Borne Dis* 2021; **12**: 101759.

359. Gryczyńska A, Sokół M, Gortat T, Kowalec M. *Borrelia miyamotoi* infection in *Apodemus* spp. mice populating an urban habitat (Warsaw, Poland). *Int J Parasitol Parasites Wildl* 2021; **14**: 138–40.

360. Grech-Angelini S, Stachurski F, Vayssier-Taussat M, et al. Tick-borne pathogens in ticks (Acari: Ixodidae) collected from various domestic and wild hosts in Corsica (France), a Mediterranean island environment. *Transbound Emerg Dis* 2020; **67**: 745–57.

361. Goutier S, Ferquel E, Pinel C, et al. *Borrelia crocidurae* meningoencephalitis, West Africa. *Emerg Infect Dis* 2013; **19**: 301–304.

362. Ghasemi A, Naddaf SR, Mahmoudi A, et al. *Borrelia duttonii*-like spirochetes parasitize *Meriones persicus* in East Azerbaijan Province of Iran. *Ticks Tick Borne Dis* 2021; **12**: 101825.

363. Gebrehiwot T, Fiseha A. Tetracycline versus penicillin in the treatment of louse-borne relapsing fever. *Ethiop Med J* 1992; **30**: 175–81.

364. Gaud M, Morgan MT. Epidemiological study on relapsing fever in North Africa (1943-1945). *Bull World Health Organ* 1948; **1**: 69–92.

365. Gaud M, Bey MK, Vaucel M. The Evolution of the epidemic of relapsing fever, 1942-1946. *Bull World Health Organ* 1948; **1**: 93–101.

366. Garcia-Vozmediano A, Krawczyk AI, Sprong H, et al. Ticks climb the mountains: Ixodid tick infestation and infection by tick-borne pathogens in the Western Alps. *Ticks Tick Borne Dis* 2020; **11**: 101489.

367. Gambles RM, Coghill NF. Relapsing fever in Cyprus. *Ann Trop Med Parasitol* 1948; **42**: 288–303.

368. Galun E, Ben-Chetrit E. Possible prevention of tick-borne relapsing fever in patients infected with *Borrelia recurrentis*. *J Infect Dis* 1984; **150**: 617.

369. Gage KL, Eggleston ME, Gilmore RD, Jr., et al. Isolation and characterization of *Borrelia parkeri* in *Ornithodoros parkeri* (Ixodida: Argasidae) collected in Colorado. *J Med Entomol* 2001; **38**: 665–74.

370. Gaber MS, Khalil GM, Hoogstraal H. *Borrelia crocidurae*: venereal transfer in Egyptian *Ornithodoros erraticus* ticks. *Exp Parasitol* 1982; **54**: 182–84.

371. Fuchs PC, Oyama AA. Neonatal relapsing fever due to transplacental transmission of *Borrelia*. *JAMA* 1969; **208**: 690–92.

372. Fryxell RT, Steelman CD, Szalanski AL, et al. Survey of Borreliae in ticks, canines, and white-tailed deer from Arkansas, U.S.A. *Parasit Vectors* 2012; **5**: 139.

373. Fritz CL, Payne JR, Schwan TG. Serologic evidence for *Borrelia hermsii* infection in rodents on federally owned recreational areas in California. *Vector Borne Zoonotic Dis* 2013; **13**: 376–81.

374. Fritz CL, Bronson LR, Smith CR, et al. Isolation and characterization of *Borrelia hermsii* associated with two foci of tick-borne relapsing fever in California. *J Clin Microbiol* 2004; **42**: 1123–28.

375. Fraenkel CJ, Garpmo U, Berglund J. Determination of novel *Borrelia* genospecies in Swedish *Ixodes ricinus* ticks. *J Clin Microbiol* 2002; **40**: 3308–12.

376. Fowler J. A case of relapsing fever in the central provinces. *Ind Med Gaz* 1909; **44**: 460.

377. Fischer RJ, Johnson TL, Raffel SJ, Schwan TG. Identical strains of *Borrelia hermsii* in mammal and bird. *Emerg Infect Dis* 2009; **15**: 2064–66.

378. Fihn S, Larson EB. Tick-borne relapsing fever in the Pacific Northwest: an underdiagnosed illness? *West J Med* 1980; **133**: 203–09.

379. Fesler MC, Shah JS, Middelveen MJ, et al. Lyme disease: diversity of *Borrelia* species in California and Mexico detected using a novel immunoblot assay. *Healthcare (Basel),* 2020; **8**: 97.

380. Felder H, Hoekstra KA. *Borrelia hermsii* relapsing fever. *Blood* 2014; **123**: 160.

381. Fedorova N, Kleinjan JE, James D, et al. Remarkable diversity of tick or mammalian-associated *Borreliae* in the metropolitan San Francisco Bay Area, California. *Ticks Tick Borne Dis* 2014; **5**: 951–61.

382. Fall NS, Diagne N, Mediannikov O, et al. Detection of *Borrelia crocidurae* in a vaginal swab after miscarriage, rural Senegal, Western Africa. *Int J Infect Dis* 2020; **91**: 261–63.

383. Evans NJ, Bown K, Timofte D, Simpson VR, Birtles RJ. Fatal borreliosis in bat caused by relapsing fever spirochete, United Kingdom. *Emerg Infect Dis* 2009; **15**: 1331–33.

384. Esteve-Gasent MD, Snell CB, Adetunji SA, Piccione J. Serological detection of tick-borne relapsing fever in Texan domestic dogs. *PLoS One* 2017; **12**: e0189786.

385. Eshoo MW, Crowder CD, Carolan HE, et al. Broad-range survey of tick-borne pathogens in Southern Germany reveals a high prevalence of *Babesia microti* and a diversity of other tick-borne pathogens. *Vector Borne Zoonotic Dis* 2014; **14**: 584–91.

386. Eshoo MW, Carolan HE, Massire C, et al. Survey of *Ixodes pacificus* ticks in California reveals a diversity of microorganisms and a novel and widespread Anaplasmataceae species. *PLoS One* 2015; **10**: e0135828.

387. Ellis L, Curtis MW, Gunter SM, Lopez JE. Relapsing fever infection manifesting as aseptic meningitis, Texas, USA. *Emerg Infect Dis* 2021; **27**: 2681–85.

388. Eisenberg S, Gunders AE, Cohen AM. Tick-borne relapsing fever in the Judean hills, including a case with massive haematuria. *Trans R Soc Trop Med Hyg* 1968; **62**: 679–81.

389. Elbir H, FotsoFotso A, Diatta G, et al. Ubiquitous bacteria *Borrelia crocidurae* in Western African ticks *Ornithodoros sonrai*. *Parasit Vectors* 2015; **8**: 477.

390. Edwards MJ, Russell JC, Davidson EN, et al. A 4-yr survey of the range of ticks and tick-borne pathogens in the Lehigh Valley Region of Eastern Pennsylvania. *J Med Entomol* 2019; **56**: 1122–34.

391. Edell TA, Emerson JK, Maupin GO, Barnes AM, Vernon TM. Tick-borne relapsing fever in Colorado. Historical review and report of cases. *JAMA* 1979; **241**: 2279–82.

392. Dykstra EA, Oltean HN, Kangiser D, et al. Ecology and Epidemiology of tickborne pathogens, Washington, USA, 2011-2016. *Emerg Infect Dis* 2020; **26**: 648–57.

393. Dupont HT, La Scola B, Williams R, Raoult D. A focus of tick-borne relapsing fever in southern Zaire. *Clin Infect Dis* 1997; **25**: 139–44.

394. Duplaix L, Wagner V, Gasmi S, et al. Exposure to tick-borne pathogens in cats and dogs infested with *Ixodes scapularis* in Quebec: An 8-year surveillance study. *Front Vet Sci* 2021; **8**: 696815.

395. Dibernardo A, Cote T, Ogden NH, Lindsay LR. The prevalence of *Borrelia miyamotoi* infection, and co-infections with other *Borrelia* spp. in *Ixodes scapularis* ticks collected in Canada. *Parasit Vectors* 2014; **7**: 183.

396. Díaz P, Remesar S, Venzal JM, et al. Occurrence of *Borrelia* and *Borreliella* species in *Ixodes ricinus* collected from roe deer in northwestern Spain. *Med Vet Entomol* 2019; **33**: 427–30.

397. Diatta G, Souidi Y, Granjon L, et al. Epidemiology of tick-borne borreliosis in Morocco. *PLoS Negl Trop Dis* 2012; **6**: e1810.

398. Diatta G, Duplantier JM, Granjon L, et al. *Borrelia* infection in small mammals in West Africa and its relationship with tick occurrence inside burrows. *Acta Trop* 2015; **152**: 131–40.

399. Diallo MA, Kane BS, Ndiaye M, et al. *Plasmodium falciparum* malaria co-infection with tick-borne relapsing fever in Dakar. *Malar J* 2017; **16**: 24.

400. Delaney SL, Murray LA, Aasen CE, et al. *Borrelia miyamotoi* serology in a clinical population with persistent symptoms and suspected tick-borne illness. *Front Med (Lausanne)* 2020; **7**: 567350.

401. de Jong J, Wilkinson RJ, Schaeffers P, Sondorp HE, Davidson RN. Louse-borne relapsing fever in southern Sudan. *Trans R Soc Trop Med Hyg* 1995; **89**: 621.

402. De Jesus CE, Ganser C, Kessler WH, et al. A survey of tick-borne bacterial pathogens in Florida. *Insects* 2019; **10**: 297.

403. Davis RD, Burke JP, Wright LJ. Relapsing fever associated with ARDS in a parturient woman. A case report and review of the literature. *Chest,* 1992; **102**: 630–32.

404. Cutler S, Abdissa A, Adamu H, Tolosa T, Gashaw A. Borrelia in Ethiopian ticks. *Ticks Tick Borne Dis* 2012; **3**: 14–17.

405. Cull B, Hansford KM, McGinley L, et al. A nationwide study on *Borrelia burgdorferi* s.l. infection rates in questing *Ixodes ricinus*: a six-year snapshot study in protected recreational areas in England and Wales. *Med Vet Entomol* 2021; **35**: 352–60.

406. Cuevas LE, Borgnolo G, Hailu B, et al. Tumour necrosis factor, interleukin-6 and C-reactive protein in patients with louse-borne relapsing fever in Ethiopia. *Ann Trop Med Parasitol* 1995; **89**: 49–54.

407. Crowder CD, Carolan HE, Rounds MA, et al. Prevalence of *Borrelia miyamotoi* in *Ixodes* ticks in Europe and the United States. *Emerg Infect Dis* 2014; **20**: 1678–82.

409. Colebunders R, De Serrano P, Van Gompel A, et al. Imported relapsing fever in European tourists. *Scand J Infect Dis* 1993; **25**: 533–36.

410. Coghill NF, Lawrence J, Ballantine ID. Relapsing fever in Cyrenaica. *Br Med J* 1947; **1**: 637–40.

411. Coghill NF. Treatment of tick-borne relapsing fever. Neoarsphenamine and other remedies. *Lancet* 1951; **1**: 604–605.

412. Coene J. Relapsing fever in Sichili. *Med J Zambia,* 1984; **18**: 10–14.

413. Cleary M, Theis J. Identification of a novel strain of *Borrelia hermsii* in a previously undescribed northern California focus. *Am J Trop Med Hyg* 1999; **60**: 883–87.

414. Clark K. *Borrelia* species in host-seeking ticks and small mammals in northern Florida. *J Clin Microbiol* 2004; **42**: 5076–86.

415. Chowdri HR, Gugliotta JL, Berardi VP, et al. *Borrelia miyamotoi* infection presenting as human granulocytic anaplasmosis: a case report. *Ann Intern Med* 2013; **159**: 21–27.

416. Charters AD. Tick-borne relapsing fever in Somaliland with special reference to the blood sedimentation rate. *Trans R Soc Trop Med Hyg* 1950; **43**: 427–34.

417. Castilla-Guerra L, Alvarez-Suero J, Del Carmen Fernandez-Moreno M, Fontana ER. Tick-borne relapsing fever: conjunctival haemorrhages. *BMJ Case Rep* 2009; **2009**: 114.

418. Castellaw AH, Showers J, Goddard J, Chenney EF, Varela-Stokes AS. Detection of vector-borne agents in lone star ticks, *Amblyomma americanum* (Acari: Ixodidae), from Mississippi. *J Med Entomol* 2010; **47**: 473–76.

419. Castellaw AH, Chenney EF, Varela-Stokes AS. Tick-borne disease agents in various wildlife from Mississippi. *Vector Borne Zoonotic Dis* 2011; **11**: 439–42.

420. Capligina V, Seleznova M, Akopjana S, et al. Large-scale countrywide screening for tick-borne pathogens in field-collected ticks in Latvia during 2017-2019. *Parasit Vectors* 2020; **13**: 351.

421. Campbell SB, Klioueva A, Taylor J, et al. Evaluating the risk of tick-borne relapsing fever among occupational cavers-Austin, TX, 2017. *Zoonoses Public Health* 2019; **66**: 579–86.

422. Burkot TR, Mullen GR, Anderson R, et al. *Borrelia lonestari* DNA in adult *Amblyomma americanum* ticks, Alabama. *Emerg Infect Dis* 2001; **7**: 471–73.

423. Coleman GE. Relapsing fever in California. *Cal West Med* 1933; **39**: 420.

424. Brown V, Larouze B, Desve G, et al. Clinical presentation of louse-borne relapsing fever among Ethiopian refugees in northern Somalia. *Ann Trop Med Parasitol* 1988; **82**: 499–502.

425. Brasseur D. Tick-borne relapsing fever in a premature infant. *Ann Trop Paediatr.* 1985; **5**: 161–62.

426. Boutellis A, Mediannikov O, Bilcha KD, et al. *Borrelia recurrentis* in head lice, Ethiopia. *Emerg Infect Dis* 2013; **19**: 796–98.

427. Bouattour A, Garnier M, M'Ghirbi Y, et al. *Borrelia crocidurae* infection of *Ornithodoros erraticus* (Lucas, 1849) ticks in Tunisia. *Vector Borne Zoonotic Dis* 2010; **10**: 825–30.

428. Bottieau E, Verbruggen E, Aubry C, Socolovschi C, Vlieghe E. Meningoencephalitis complicating relapsing fever in traveller returning from Senegal. *Emerg Infect Dis* 2012; **18**: 697–98.

429. Bottieau E, Verbruggen E, Aubry C, Socolovschi C, Vlieghe E. Study on the infection of taiga ticks with *Borrelia* in the territory of Novosibirsk Scientific Center SB PAS. *Parazitologiia* 2010; **44**: 543–56.

430. Borgnolo G, Hailu B, Chiabrera F. Louse-borne relapsing fever in Ethiopia. *Lancet* 1991; **338**: 827.

431. Boden K, Lobenstein S, Hermann B, Margos G, Fingerle V. *Borrelia miyamotoi*-associated neuroborreliosis in immunocompromised person. *Emerg Infect Dis* 2016; **22**: 1617–20.

432. Billiet A, Vanderschueren S, Lagrou K, et al. Tick borne relapsing fever after travelling to a Greek island. *J Travel Med* 2021; **29**: 73.

433. Bhandarkar PR. Relapsing fever and the louse. *Ind Med Gaz* 1909; **44**: 433.

434. Bermúdez SE, Gottdenker N, Krishnvajhala A, et al. Synanthropic mammals as potential hosts of tick-borne pathogens in Panama. *PLoS One* 2017; **12**: e0169047.

435. Bechtel MJ, Drake KK, Esque TC, et al. Borreliosis transmission from ticks associated with desert tortoise burrows: examples of tick-borne relapsing fever in the Mojave Desert. *Vector Borne Zoonotic Dis* 2021; **21**: 635–37.

436. Barmaki A, Rafinejad J, Vatandoost H, et al. Study on presence of *Borrelia persica* in soft ticks in Western Iran. *Iran J Arthropod Borne Dis* 2010; **4**: 19–25.

437. Barbour AG, Bunikis J, Travinsky B, et al. Niche partitioning of *Borrelia burgdorferi* and *Borrelia miyamotoi* in the same tick vector and mammalian reservoir species. *Am J Trop Med Hyg* 2009; **81**: 1120–31.

438. Banović P, Díaz-Sánchez AA, Galon C, et al. Humans infested with *Ixodes ricinus* are exposed to a diverse array of tick-borne pathogens in Serbia. *Ticks Tick Borne Dis* 2021; **12**: 101609.

439. Baneth G, Nachum-Biala Y, Halperin T, et al. *Borrelia persica* infection in dogs and cats: clinical manifestations, clinicopathological findings and genetic characterization. *Parasit Vectors* 2016; **9**: 244.

440. Banerjee SN, Banerjee M, Fernando K, Burgdorfer W, Schwan TG. Tick-borne relapsing fever in British Columbia, Canada: first isolation of *Borrelia hermsii*. *J Clin Microbiol* 1998; **36**: 3505–08.

441. Bacon RM, Gilmore RD, Jr., Quintana M, Piesman J, Johnson BJ. DNA evidence of *Borrelia lonestari* in *Amblyomma americanum* (Acari: Ixodidae) in southeast Missouri. *J Med Entomol* 2003; **40**: 590–92.

442. Babudieri B. Relapsing fever in Jordan. *Bull World Health Organ,* 1957; **16**: 911–28.

443. Atkinson RC. Relapsing fever. *Cal West Med* 1936; **45**: 172.

444. Assous MV, Wilamowski A, Bercovier H, Marva E. Molecular characterization of tickborne relapsing fever *Borrelia*, Israel. *Emerg Infect Dis* 2006; **12**: 1740–43.

445. Ashbel R. Notes on spirochaeta persica from Palestine and spirochaetes of relapsing fever from the Western Desert (Tobruk area). *Trans R Soc Trop Med Hyg* 1949; **42**: 409.

446. Armstrong ER. Two cases of relapsing fever. *Ind Med Gaz* 1914; **49**: 79.

447. Armstrong BA, Kneubehl A, Krishnavajhala A, et al. Seroprevalence for the tick-borne relapsing fever spirochete *Borrelia turicatae* among small and medium sized mammals of Texas. *PLoS Negl Trop Dis* 2018; **12**: e0006877.

448. Antinori S, Tonello C, Edouard S, et al. Diagnosis of louse-borne relapsing fever despite negative microscopy in two asylum seekers from Eastern Africa. *Am J Trop Med Hyg* 2017; **97**: 1669–72.

449. Anderson IG. A note on relapsing fever occurring in two Europeans. *Cent Afr J Med* 1958; **4**: 444–45.

450. Amanzougaghene N, Akiana J, Mongo Ndombe G, et al. Head lice of pygmies reveal the presence of relapsing fever Borreliae in the Republic of Congo. *PLoS Negl Trop Dis* 2016; **10**: e0005142.

451. Anda P, Sánchez-Yebra W, del Mar Vitutia M, et al. A new *Borrelia* species isolated from patients with relapsing fever in Spain. *Lancet* 1996; **348**: 162–65.

452. Lawaczeck EW, Mead PS, Schriefer ME, Brett ME, McCollum JT. Tickborne relapsing fever in a mother and newborn child-Colorado, 2011. *Morb Mortal Wkly Rep* 2012; **61**: 174–76.

453. Centers for Disease Control and Prevention. Tickborne relapsing fever outbreak after a family gathering--New Mexico, August 2002. *Morb Mortal Wkly Rep* 2003; **52**: 809–12.

454. Binetruy F, Garnier S, Boulanger N, et al. A novel *Borrelia* species, intermediate between Lyme disease and relapsing fever groups, in neotropical passerine-associated ticks. *Sci Rep*. 2020; **10:** 10596.

455. Centers for Disease C. Common source outbreak of relapsing fever-California. *Morb Mortal Wkly Rep* 1990; **39**: 579, 585–76.

456. Almaviva M, Hailu B, Borgnolo G, et al. Louse-borne relapsing fever epidemic in Arssi Region, Ethiopia: a six months survey. *Trans R Soc Trop Med Hyg* 1993; **87**: 153.

457. Al-Gwaiz LA, Al-Mashhadani SA, Ayoola EA, et al. Relapsing fever in Saudi Arabia: Report of two cases. *Ann Saudi Med* 1995; **15**: 165–67.

458. Agarwal BL. Relapsing fever in Kashmir. *Ind Med Gaz* 1951; **86**: 446–53.

459. Zumpt F. Is the multimammate rat a natural reservoir of *Borrelia duttoni*? *Nature* 1959; **184**: 793–94.

460. Aghighi Z, Assmar M, Piazak N, et al. Distribution of soft ticks and their natural infection with *Borrelia* in a focus of relapsing fever in Iran. *Iranian J Arthropod-Borne Dis,* 2007; **1**: 14–18.

461. Aslam B, Hussain I, Mahmood MS, Sajjad ur R, Siddique AB. Flagellin gene based phylogenetic analysis of Pakistani strain of *Borrelia anserina* isolated from *Argas* ticks. *Afr J Biomed Res,* 2012; **6**: 3214–21.

462. Lebredo MG. A case of recurrent fever observed in Havana. *Public Health Pap Rep,* 1906; **32**: 238–47.

463. Cosson J-F, Michelet L, Chotte J, et al. Genetic characterization of the human relapsing fever spirochete *Borrelia miyamotoi* in vectors and animal reservoirs of Lyme disease spirochetes in France. *Parasites Vectors,* 2014; **7**: 233.

464. Diaz P, Luis Arnal J, Remesar S, et al. Molecular identification of *Borrelia* spirochetes in questing *Ixodes ricinus* from northwestern Spain. *Parasites Vectors,* 2017; **10**: 615.

465. Filatov S, Krishnavajhala A, Armstrong BA, et al. Isolation and molecular characterization of tick-borne relapsing fever *Borrelia* infecting *Ornithodoros* (Pavlovskyella) *verrucosus* ticks collected in Ukraine. *J Infect,* 2020; **221**: 804–11.

466. Jahfari S, Herremans T, Platonov AE, et al. High seroprevalence of *Borrelia miyamotoi* antibodies in forestry workers and individuals suspected of human granulocytic anaplasmosis in the Netherlands. *New Microbes New Infect,* 2014; **2**: 144–49.

467. Kalmar Z, Dumitrache MO, D'Amico G, et al. Multiple tick-borne pathogens in *Ixodes ricinus* ticks collected from humans in Romania. *Pathogens* 2020; **9**: 390.

468. Jones JM, Schumacher M, Peoples M, et al. Tickborne relapsing fever outbreak at an outdoor education camp - Arizona, 2014. *Morb Mortal Wkly Rep* 2015; **64**: 651–52.

469. Kelly AL, Raffel SJ, Fischer R, et al. First isolation of the relapsing fever spirochete, *Borrelia herrnsii*, from a domestic dog. *Ticks and Tick-Borne Dis* 2014; **5**: 95–99.

470. Koyo CSB, Amanzougaghene N, Davoust B, et al. Genetic diversity of human head lice and molecular detection of associated bacterial pathogens in Democratic Republic of Congo. *Parasites Vectors,* 2019; **12**: 290.

471. Lee S-H, Chong S-T, Kim H-C, et al. Surveillance and molecular identification of *Borrelia* Species in ticks collected at U.S. Army Garrison Humphreys, Republic of Korea, 2018-2019. *J Med Entomol* 2020; **59**: 363–71.

472. Lejal E, Marsot M, Chalvet-Monfray K, et al. A three-years assessment of *Ixodes ricinus*-borne pathogens in a French peri-urban forest. *Parasites Vectors,* 2019; **12**: 551.

473. Ruyts SC, Frazer-Mendelewska E, Van den Berge K, Verheyen K, Sprong H. Molecular detection of tick-borne pathogens *Borrelia afzelii*, *Borrelia miyamotoi* and *Anaplasma phagocytophilum* in Eurasian red squirrels (Sciurus vulgaris). *Eur J Wildlife Res*, 2017; **63**: 43.

474. Stauffer MT, Mandli J, Pritt BS, et al. Detection of zoonotic human pathogens from *Ixodes scapularis* in Wisconsin. *J Vector Ecol* 2020; **45**: 147–49.

476. Sarksyan DS, Platonov AE, Karan LS, et al. Probability of spirochete *Borrelia miyamotoi* transmission from ticks to humans. *Emerg Infect Dis* 2015; **21**: 2273–74.

477. Rafinejad J, Shemshad K, Banafshi O. Epidemiological study on tick-borne (acari: argasidae) relapsing fever in Kurdistan province, Iran, 2000-2004. *Florida Entomologist* 2012; **95**: 758–63.

478. Scott JC, Wright DJM, Cutler SJ. Typing African relapsing fever spirochetes. *Emerg Infect Dis* 2005; **11**: 1722–29.

479. Bunikis J, Barbour AG. Third *Borrelia* species in white-footed mice. *Emerg Infect Dis* 2005; **11**: 1150–51.

480. Stete K, Rieg S, Margos G, et al. Case report and genetic sequence analysis of *Candidatus* Borrelia kalaharica, Southern Africa. *Emerg Infect Dis* 2018; **24**: 1659–64.

481. Sudhindra P, Wang G, Schriefer ME, et al. Insights into *Borrelia miyamotoi* infection from an untreated case demonstrating relapsing fever, monocytosis and a positive C6 Lyme serology. *Diagn. Microbiol Infect Dis* 2016; **86**: 93–96.

482. Venczel R, Knoke L, Pavlovic M, et al. A novel duplex real-time PCR permits simultaneous detection and differentiation of *Borrelia miyamotoi* and *Borrelia burgdorferi* sensu lato. *Infection* 2016; **44**: 47–55.

483. Von Both U, Alberer M. *Borrelia recurrentis* infection. *N Engl J Med* 2016; **375**: 5.

484. Walker RL, Read DH, Hayes DC, Nordhausen RW. Equine abortion associated with the *Borrelia parkeri*-*B. turicatae* tick-borne relapsing fever spirochete group. *J Clin Microbiol* 2002; **40**: 1558–62.

485. Centers for Disease C Prevention. Prevention tickborne relapsing fever outbreak after a family gathering: New Mexico, August 2002. *Morb Mortal Wkly Rep* 2003; **52**: 809–12.

486. Morales-Diaz J, Colunga-Salas P, Romero-Salas D, et al. Molecular detection of reptile-associated *Borrelia* in *Boa constrictor* (Squamata: Boidae) from Veracruz, Mexico. *Acta Trop* 2020; **205**: 105422.

488. Medkour H, Laidoudi Y, Marié JL, et al. Molecular investigation of vector-borne pathogens in red foxes (*vulpes vulpes*) from southern France. *J Wildl Dis* 2020; **56**: 837–50.

489. Margos G, Pantchev N, Globokar M, et al. First cases of natural infections with *Borrelia hispanica* in two dogs and a cat from Europe. *Microorganisms* 2020; **8**: 1251.

490. Mancini F, Innocenti P, Baumgartner M, et al. *Borrelia microti* infection in an Italian woman returning from Kyrgyzstan and Tajikistan. *Travel Med Infect Dis* 2020; **35**: 101448.

491. Lau ACC, Qiu Y, Moustafa MAM, et al. Detection of *Borrelia burgdorferi* sensu lato and Relapsing fever *Borrelia* in feeding *Ixodes* ticks and rodents in Sarawak, Malaysia: New geographical records of *Borrelia yangtzensis* and *Borrelia miyamotoi*. *Pathogens* 2020; **9**: 846.

492. Guggenheim JN, Haverkamp AD. Tick-borne relapsing fever during pregnancy: a case report. *J Reprod Med* 2005; **50**: 727–29.

493. Colunga-Salas P, Sánchez-Montes S, León-Paniagua L, Becker I. *Borrelia* in neotropical bats: Detection of two new phylogenetic lineages. *Ticks Tick Borne Dis* 2021; **2**: 101642.

494. Hojgaard A, Osikowicz LM, Eisen L, Eisen RJ. Evaluation of a novel multiplex PCR amplicon sequencing assay for detection of human pathogens in *Ixodes* ticks. *Ticks Tick Borne Dis* 2020; **11**: 101504.

495. N. Piazak, Seyedi Rashti SMA, M Asmar. A survey of prevalence of *Ornithodorus tartakovskyi* and its infection rate with *Borrelia latychevi* in Sarakhs county, Khorassan province. *Iran J Public Health* 2000; **29**: 103–08.

496. Gondard M, Delannoy S, Pinarello V, et al. Upscaling the surveillance of tick-borne pathogens in the French Caribbean Islands. *Pathogens* 2020; **9**: 176.

497. Ghafar A, Cabezas-Cruz A, Galon C, et al. Bovine ticks harbour a diverse array of microorganisms in Pakistan. *Parasit Vectors* 2020; **13**: 1.

498. Dahmana H, Granjon L, Diagne C, et al. Rodents as hosts of pathogens and related zoonotic disease risk. *Pathogens* 2020; **9**: 202.

499. Cleveland CA, Swanepoel L, Brown JD, et al. Surveillance for *Borrelia* spp. in upland game birds in Pennsylvania, USA. *Vet Sci* 2020; **7**: 82.

500. Rampes S YM, Galloway. Tick-borne relapsing fever: a fever syndrome mimic. *Rheumatol Adv Pract,* 2019; **3**: 23.

501. Rudakova SA, Teslova OE, Kaneshova NE, et al. Genospecies diversity of *Borrelia* in *Ixodes* ticks of the West Siberia. *Problemy Osobo Opasnykh Infektsii* 2019; **4**: 92–96.

502. Szekeres S, Lügner J, Fingerle V, Margos G, et al. Prevalence of *Borrelia burgdorferi* sensu lato and *Borrelia miyamotoi* in ixodid ticks in the Far East of Russia. *Int J Parasitol Parasites Wildl* 2019; **8**: 192–202.

503. B. Weller, G. M. Graham. Relapsing fever in central Texas. *JAMA* 1930; **95**: 1834–35.

504. Magnarelli LA, Anderson JF, Johnson RC. Cross-reactivity in serological tests for Lyme disease and other spirochetal infections. *J Infect Dis* 1987; **156**: 183–88.

505. Barbour AG, Maupin GO, Teltow GJ, Carter CJ, Piesman J. Identification of an uncultivable *Borrelia* species in the hard tick *Amblyomma americanum*: Possible agent of a Lyme disease-like illness. *J Infect Dis* 1996; **173**: 403–09.

506. Ali Majidpour. A case of *Borrelia* meningitis. *Arch of Iranian Med* 2003; **6**: 222–23.

507. Shahnam Arshi AM, Homayoun Sadeghi, Mehdi Asmar, Darioush Emdadi, Mohammad Hossein Derakhshan. Relapsing fever in Ardabil, a northwestern province of Iran. *Arch Iran Med* 2002; **5**: 141–45.

508. Loftis AD, Gill JS, Schriefer ME, et al. Detection of *Rickettsia, Borrelia*, and *Bartonella* in *Carios kelleyi* (Acari: Argasidae). *J Med Entomol* 2005; **42**: 473–80.

509. Pampana, E. J. Colombian relapsing fever simulating acute appendicitis. *Trans R Soc Trop Med Hyg* 1927; **20**: 357–58.

510. Muñoz-Leal S, Marcili A, Fuentes-Castillo D, Ayala M, Labruna MB. A relapsing fever *Borrelia* and spotted fever *Rickettsia* in ticks from an Andean valley, central Chile. *Exp Appl Acarol* 2019; **78**: 403–20.

511. Modarelli JJ, Tomecek JM, Piccione J, Ferro PJ, Esteve-Gasent MD. Molecular prevalence and ecoregion distribution of select tick-borne pathogens in Texas dogs. *Transbound Emerg Dis* 2019; **66**: 1291–300.

512. Morel N, De Salvo MN, Cicuttin G, et al. The presence of *Borrelia theileri* in Argentina. *Vet Parasitol Reg Stud Reports* 2019; **17**: 100314.

513. Lambregts MMC, Bentvelsen RG, Makiello PE, et al. Relapsing fever after travelling in the tropics; a story with a twist. *Ned Tijdschr Geneeskd* 2019; **163**: D3455.

514. Jaenson TGT, Wilhelmsson P. First records of tick-borne pathogens in populations of the taiga tick *Ixodes persulcatus* in Sweden. *Parasit Vectors* 2019; **12**: 559.

515. Heida J, van Arkel A, Verweij JJ, Tijssen CC. Meningitis due to infection with *Borrelia hispanica*. *Ned Tijdschr Geneeskd* 2019; **163**: D3859.

516. Sanchez RST, Santodomingo AMS, Munoz-Leal S, et al. Rodents as potential reservoirs for *Borrelia* spp. in northern Chile. *Rev Bras Parasitol Vet* 2020; **29**: e000120.

517. Abanda B, Paguem A, Abdoulmoumini M, et al. Molecular identification and prevalence of tick-borne pathogens in zebu and taurine cattle in North Cameroon. *Parasit Vectors* 2019; **12**: 448.

518. Zhai B, Niu Q, Liu Z, et al. First detection and molecular identification of *Borrelia* species in Bactrian camel (*Camelus bactrianus*) from Northwest China. *Infect Genet Evol* 2018; **64**: 149–55.

519. M. V. Savel’eva, E. I. Krasnova, N. I. Khokhlova, N. V. Tikunova, et al. Clinical and laboratory characteristics of diseases caused by *Borrelia* spp. in the inhabitants of the Novosibirsk region in 2015–2017. *J Infectology* 2018; **10**: 68–75.

520. Piedmonte NP, Shaw SB, Prusinski MA, Fierke MK. Landscape features associated with blacklegged tick (Acari: Ixodidae) density and tick-borne pathogen prevalence at multiple spatial scales in central New York State. *J Med Entomol* 2018; **55**: 1496–508.

521. Pereira A, Parreira R, Cotão AJ, et al. Tick-borne bacteria and protozoa detected in ticks collected from domestic animals and wildlife in central and southern Portugal. *Ticks Tick Borne Dis* 2018; **9**: 225–34.

522. Palomar AM, Portillo A, Santibáñez P, Santibáñez S, Oteo JA. *Borrelia miyamotoi*: Should this pathogen be considered for the diagnosis of tick-borne infectious diseases in Spain? *Enferm Infecc Microbiol Clin (Engl Ed)* 2018; **36**: 568–71.

523. Middelveen MJ, Shah JS, Fesler MC, Stricker RB. Relapsing fever *Borrelia* in California: a pilot serological study. *Int J Gen Med* 2018; **11**: 373–82.

524. Luis Marcos, Kalie Smith, Fredric Weinbaum, Spitzer E. An emerging tick-borne disease in Long Island, New York: Relapsing fever caused by *Borrelia miyamotoi*. *Open Forum Infect Dis* 2018; **5**: 241.

525. Kulkarni M, Kryuchkov R, Statculescu A, et al. *Ixodes scapularis* tick distribution and infection rates in Ottawa, Ontario, 2017. *Can Commun Dis Rep* 2018; **10**: 237–42.

526. Krause PJ, Carroll M, Fedorova N, et al. Human *Borrelia miyamotoi* infection in California: Serodiagnosis is complicated by multiple endemic *Borrelia* species. *PLoS One* 2018; **2**: e0191725.

527. Karan L, Makenov M, Kolyasnikova N, Stukolova O, Toporkova M, Olenkova O. Dynamics of spirochetemia and early PCR detection of *Borrelia miyamotoi*. *Emerg Infect Dis* 2018; **5**: 860–67.

528. Kahouli S, Naoui H, Uwingabiye J, et al. Relapsing fever in a Moroccan man. *Med Sante Trop* 2018; **28**: 141–43.

529. Johnson TL, Graham CB, Maes SE, et al. Prevalence and distribution of seven human pathogens in host-seeking *Ixodes scapularis* (Acari: Ixodidae) nymphs in Minnesota, USA. *Ticks Tick Borne Dis* 2018; **9**: 1499–507.

530. Guiheneuf E, Desjardins N, Guiheneuf R. It is not always malaria: diagnosis of *Borrelia* recurrent fever on blood smear. *Ann Biol Clin (Paris)* 2018; **76**: 118–19.

531. Egizi A, Roegner V, Faraji A, et al. A historical snapshot of *Ixodes scapularis*-borne pathogens in New Jersey ticks reflects a changing disease landscape. *Ticks Tick Borne Dis* 2018; **9**: 418–26.

532. Cutler SJ, Idris JM, Ahmed AO, Elelu N. *Ornithodoros savignyi*, the Tick Vector of "*Candidatus* Borrelia kalaharica" in Nigeria. *J Clin Microbiol* 2018; **56**: e00532–18.

533. Andersson MO, Marga G, Banu T, Dobler G, Chitimia-Dobler L. Tick-borne pathogens in tick species infesting humans in Sibiu County, central Romania. *Parasitol Res* 2018; **117**: 1591–97.

534. Straub MH, Roy AN, Martin A, et al. Distribution and prevalence of vector-borne diseases in California chipmunks (*Tamias* spp.). *PLoS One* 2017; **12**: e0189352.

535. Raileanu C, Moutailler S, Pavel I, et al. *Borrelia* diversity and co-infection with other tick borne pathogens in ticks. *Front Cell Infect Microbiol* 2017; **7**: 36.

536. Muhammad Morshed SJD, Min-Kuang Lee, Stephanie Man, et al. Patrick tick-borne relapsing fever in British Columbia: A 10-year review (2006–2015). *BCMJ* 2017; **59**: 412–27.

537. Leen I, Bruynseels P, Mukadi BK, van Oort M, van den Akker M. A 13-year old girl with pancytopenia at the presentation of a *Borrelia hispanica* infection: a case report and review of the literature. *J Med Case Rep* 2017; **11**: 51.

538. Lafri I, El Hamzaoui B, Bitam I, et al. Detection of relapsing fever *Borrelia* spp., *Bartonella* spp. and Anaplasmataceae bacteria in argasid ticks in Algeria. *PLoS Negl Trop Dis* 2017; **11**: e0006064.

539. Honig V, Carolan HE, Vavruskova Z, et al. Broad-range survey of vector-borne pathogens and tick host identification of *Ixodes ricinus* from Southern Czech Republic. *Fems Microbiol Ecol* 2017; **93**: 129.

540. Fiorito TM, Reece, Rebecca,Flanigan, Timothy P, Silverblatt Fredric J. *Borrelia miyamotoi* polymerase chain reaction positivity on a tick-borne disease panel in an endemic region of Rhode Island. *Infect Dis Clin Pract* 2017; **25**: 250–54.

541. Christensen AM, Pietralczyk E, Lopez JE, et al. Diagnosis and management of *Borrelia turicatae* infection in febrile soldier, Texas, USA. *Emerg Infect Dis* 2017; **23**: 883–84.

542. Wodecka B, Michalik J, Lane RS, Nowak-Chmura M, Wierzbicka A. Differential associations of *Borrelia* species with European badgers (*Meles meles*) and raccoon dogs (*Nyctereutes procyonoides*) in western Poland. *Ticks Tick Borne Dis* 2016; **7**: 1010–16.

543. Skotarczak B, Wodecka B, Rymaszewska A, Adamska M. Molecular evidence for bacterial pathogens in *Ixodes ricinus* ticks infesting Shetland ponies. *Exp Appl Acarol* 2016; **69**: 179–89.

544. Taylor AJ, Vongphayloth K, Vongsouvath M. Large-scale survey for tick-borne bacteria, Khammouan Province, Laos. *Emerg Infect Dis* 2016; **22**: 1635–39.

545. M S, N P, A G, H N, M A. Tick-borne relapsing fever in Sabzevar (Khorasan Razavy Province), North-Eastern Iran. *Bangladesh J Medical Sci* 2016; **15**: 551–55.

546. Gleim ER, Garrison LE, Vello MS, et al. Factors associated with tick bites and pathogen prevalence in ticks parasitizing humans in Georgia, USA. *Parasit Vectors* 2016; **9**: 125.

547. Bermúdez SE, Armstrong BA, Domínguez L, et al. Isolation and genetic characterization of a relapsing fever spirochete isolated from *Ornithodoros puertoricensis* collected in central Panama, 2021. *PLoS Negl Trop Dis* 2021; **8**: e0009642.

548. Fingerle V, Pritsch M, Wächtler M, et al. *Candidatus* Borrelia kalaharica detected from a febrile traveler returning to Germany from vacation in Southern Africa. *PLoS Negl Trop Dis* 2016; **10**: e0004559.

549. Clow KM, Ogden NH, Lindsay LR, et al. Distribution of ticks and the risk of Lyme disease and other tick-borne pathogens of public health significance in Ontario, Canada. *Vector Borne Zoonotic Dis* 2016; **16**: 215–22.

550. Castilla-Guerra L, Fernandez-Moreno MC, Vergara-Lopez S, Merino-Rumin M, Colmenero-Camacho MA. Neurological complications of tick-borne relapsing fever. *Rev Neurol* 2016; **63**: 252–56.

551. Telford SR, Goethert HK, Molloy PJ, et al. *Borrelia miyamotoi* disease: Neither Lyme disease nor relapsing fever. *Clin Lab Med* 2015; **35**: 867–82.

552. Skar G, Snowden J. Case 2: Recurrent fever and thrombocytopenia in a 4-year-old girl. *Pediatr Rev* 2015; **36**: 130–31.

553. Sarksyan DS, Maleev VV, Platonov AE, Platonova OV, Karan LS. Relapsing (recurrent) disease caused by *Borrelia miyamotoi*. *Ter Arkh* 2015; **87**: 18–25.

554. Reiter M, Schoetta A-M, Mueller A, Stockinger H, Stanek G. A newly established real-time PCR for detection of *Borrelia miyamotoi* in *Ixodes ricinus* ticks. *Ticks and Tick-Borne Dis* 2015; **6**: 303–08.

555. Nunes M, Parreira R, Lopes N, et al. Molecular identification of *Borrelia miyamotoi* in *Ixodes ricinus* from Portugal. *Vector Borne Zoonotic Dis* 2015; **15**: 515–17.

556. Fuchs I, Tarabin S, Kafka M. Relapsing fever: Diagnosis thanks to a Vigilant Hematology Laboratory. *Vector Borne Zoonotic Dis* 2015; **15**: 446–48.

557. Fotso Fotso A, Angelakis E, Mouffok N, Drancourt M, Raoult D. Blood-borne *Candidatus* Borrelia algerica in a patient with prolonged fever in Oran, Algeria. *Am J Trop Med Hyg* 2015; **93**: 1070–73.

558. Cerar T, Korva M, Avšič-Županc T, Ružić-Sabljić E. Detection, identification and genotyping of *Borrelia* spp. in rodents in Slovenia by PCR and culture. *BMC Vet Res* 2015; **11**: 188.

559. Bilal Aslam IH, Muhammad Asif Zahoor,Muhammad Shahid Mahmood,Muhammad Hidayat Rasool. Prevalence of *Borrelia anserina* in *Argas* Ticks. *Pakistan J Zool* 2015; **47**: 1125–31.

560. Souidi Y, Boudebouch N, Ezikouri S, et al. *Borrelia crocidurae* in *Ornithodoros* ticks from northwestern Morocco: A range extension in relation to climatic change? *J Vector Ecol* 2014; **39**: 316–20.

561. Mediannikov O, Socolovschi C, Bassene H, et al. High incidence of *Borrelia crocidurae* in acute febrile patients in Senegal. *Int J Infect Dis* 2014; **21**: 218.

562. McCoy BN, Maiga O, Schwan TG. Detection of *Borrelia theileri* in *Rhipicephalus geigyi* from Mali. *Ticks and Tick-Borne Dis* 2014; **5**: 401–03.

563. Lawrence H. Dunn, Herbert C. Clark. Notes on relapsing fever in Panama with special reference to animal hosts. *AJTMH* 1933; **13**: 201–09.

564. Kassiri H, Kasiri A, Karimi M, Kasiri E, Lotfi M. The seven-year longitudinal study on relapsing fever borreliosis in Western Iran. *Asian Pacific J Trop Dis* 2014; **4**: 679–83.

565. Kassiri H, Kasiri A, Dostifar K, Lotfi M. The epidemiology of tick-borne relapsing fever in Bijar County, North-Western Iran. *J Acut Dis* 2014; **3**: 224–27.

566. Sokhna C, Mediannikov O, Fenollar F, et al. Point-of-care laboratory of pathogen diagnosis in rural Senegal. *PLoS Negl Trop Dis* 2013; **7**: e1999.

567. Gholkar N, Lehman D. Images in clinical medicine. *Borrelia hermsii* (relapsing fever). *N Engl J Med* 2013; **368**: 266.

568. Yabsley MJ, Parsons NJ, Horne EC, Shock BC, Purdee M. Novel relapsing fever *Borrelia* detected in African penguins (*Spheniscus demersus*) admitted to two rehabilitation centers in South Africa. *Parasitol Res* 2012; **110**: 1125–30.

569. Shehab KW, Banaei N. Banaei. Unexplained fever after a camping trip in the American Southwest. *J Pediatric Infect Dis Soc* 2012; **1**: 254–55.

570. Nieto N, Teglas M. Maintenance of endemic tick-borne relapsing fever (*Borrelia hermsii*) from the western United States. *Int J Infect Dis* 2012; **16**: E140.

571. Lawrence H. Dunn. Studies on the South American Tick, *Ornithodoros venezuelensis* Brumpt, in Colombia. Its prevalence, distribution, and importance as an intermediate host of relapsing fever. *J Parasitol* 1927; **13**: 249–55.

572. Reller ME, Clemens EG, Schachterle SE, et al. Multiplex 5'nuclease-quantitative PCR for diagnosis of relapsing fever in a large Tanzanian cohort. *J Clin Microbiol* 2011; **49**: 3245–49.

573. Reis C, Cote M, Paul RE, Bonnet S. Questing ticks in suburban forest are infected by at least six tick-borne pathogens. *Vector Borne Zoonotic Dis* 2011; **11**: 907–16.

574. Padgett KA, Bonilla DL. Bonilla. Novel exposure sites for nymphal *Ixodes pacificus* within picnic areas. *Ticks and Tick-Borne Dis* 2011; **2**: 191–95.

575. García-Soler P, Núñez-Cuadros E, Milano-Manso G, Ruiz Sánchez P. Severe Jarisch-Herxheimer reaction in tick-borne relapsing fever. *Enferm Infecc Microbiol Clin* 2011; **29**: 710–11.

576. Fritzen CM, Huang J, Westby K, et al. Infection prevalences of common tick-borne pathogens in adult lone star ticks (*Amblyomma americanum*) and American dog ticks (*Dermacentor variabilis*) in Kentucky. *Am J Trop Med Hyg* 2011; **85**: 718–23.

577. Tokarz R, Jain K, Bennett A, Briese T, Lipkin WI. Assessment of polymicrobial infections in ticks in New York state. *Vector Borne Zoonotic Dis* 2010; **10**: 217–21.

578. Borgoiakov VIu, Fomenko NV, Panov VV, Chikova ED. Infestation of taiga ticks with *Borrelia* in the territory of Novosibirsk Scientific Center (Siberian Branch, Russian Academy of Sciences). *Parazitologiya* 2010; **44**: 543–56.

579. Pilz, H. Mooser, H. La fiebre recurrente en Aguascalientes. *Boletín del Instituto de Higien* 1936; **2**: 295–300.

580. Gholamreza Pouladfar, Alborzi Abdolvahab, Bahman Pourabbas. Tick-borne relapsing fever, a neglected cause of fever in Fars Province. *Iran J Basic Med Sci* 2008; **33**: 177–79.

581. Aher AR, Shah H, Rastogi V, Tukaram PK, Choudhury RC. A case report of relapsing fever. *Indian J Pathol Microbiol* 2008; **51**: 292–93.

582. Nordstrand A, Bunikis I, Larsson C, et al. Tickborne relapsing fever diagnosis obscured by malaria, Togo. *Emerg Infect Dis* 2007; **13**: 117–23.

583. Gallien S, Sarfati C, Haas L, Lagrange-Xelot M, Molina JM. Borreliosis: a rare and alternative diagnosis in travellers' febrile illness. *Travel Med Infect Dis* 2007; **5**: 247–50.

584. Tordini G, Giaccherini R, Corbisiero R, Zanelli G. Relapsing fever in a traveller from Senegal: determination of *Borrelia* species using molecular methods. *Trans R Soc Trop Med Hyg* 2006; **100**: 992–94.

585. Heerdink G, Petit PL, Hofwegen H, van Genderen PJ. A patient with fever following a visit to the tropics: tick-borne relapsing fever discovered in a thick blood smear preparation. *Ned Tijdschr Geneeskd* 2006; **150**: 2386–89.

586. Hasin T, Davidovitch N, Cohen R, et al. Postexposure treatment with doxycycline for the prevention of tick-borne relapsing fever. *N Engl J Med* 2006; **355**: 148–55.

587. Pichon B, Rogers M, Egan D, Gray J. Blood-meal analysis for the identification of reservoir hosts of tick-borne pathogens in Ireland. *Vector Borne Zoonotic Dis* 2005; **5**: 172–80.

588. Lane RS, Mun J, Parker JM, White M. Columbian black-tailed deer (*Odocoileus hemionus columbianus*) as hosts for *Borrelia* spp. in northern California. *J Wildl Dis* 2005; **41**: 115–25.

589. Güner ES, Hashimoto N, Kadosaka T, Imai Y, Masuzawa T. A novel, fast-growing *Borrelia* sp. isolated from the hard tick *Hyalomma aegyptium* in Turkey. *Microbiology (Reading)* 2003; **149**: 2539–44.

590. Webster G, Schiffman JD, Dosanjh AS, et al. Jarisch-Herxheimer reaction associated with ciprofloxacin administration for tick-borne relapsing fever. *Pediatr Infect Dis J* 2002; **21**: 571–73.

591. Chatel G, Gulletta M, Matteelli A, et al. Short report: diagnosis of tick-borne relapsing fever by the quantitative buffy coat fluorescence method. *Am J Trop Med Hyg* 1999; **60**: 738–39.

592. Talbert A, Nyange A, Molteni F. Spraying tick-infested houses with lambda-cyhalothrin reduces the incidence of tick-borne relapsing fever in children under five years old. *Trans R Soc Trop Med Hyg* 1998; **92**: 251–53.

593. vanHolten J, Tiems J, Jongen V. Neonatal *Borrelia duttoni* infection: A report of three cases. *Trop Doct* 1997; **27**: 115–16.

594. Poulsen LW, Iversen G. Relapsing fever: a differential diagnosis to malaria. *Scand J Infect Dis* 1996; **28**: 419–20.

595. Newton JA, Pepper PV. Images in clinical medicine. Relapsing fever. *N Engl J Med* 1996; **335**: 1197.

596. Keung YK, Cobos E, Kimbrough RC, Carver RC. Borreliosis as a cause of fever in a woman who recently returned from Saudi Arabia. *Clin Infect Dis* 1995; **21**: 447–48.

597. Diatta G, Trape JF, Legros F, Rogier C, Duplantier JM. A comparative study of three methods of detection of *Borrelia crocidurae* in wild rodents in Senegal. *Trans R Soc Trop Med Hyg* 1994; **88**: 423–24.

598. Lovett MA, Goldstein EJ, Fleischmann J. Fever in a couple vacationing in the mountains of southern California. *Clin Infect Dis* 1992; **14**: 1254–58.

599. Rose I, Yoshimizu MH, Bonilla DL, et al. Phylogeography of *Borrelia* spirochetes in *Ixodes pacificus* and *Ixodes spinipalpis* ticks highlights differential acarological risk of tick-borne disease transmission in northern versus southern California. *PLoS One* 2019; **14**: e0214726.

600. T. G. Evans, J. Kurrus, S. Magarian. Non-seasonal relapsing fever in Utah. *Clin. Microbiol, Newsl* 1992; **14**: 105–12.

601. López-Cortés L, Lozano de León F, Gómez-Mateos JM, Sánchez-Porto A, Obrador C. Tick-borne relapsing fever in intravenous drug abusers. *J Infect Dis* 1989; **159**: 804.

602. Shaked Y, Maier MK, Samra Y. Relapsing fever and salmonella bacteraemia simultaneously affecting a healthy young man. *J Infect* 1986; **13**: 308–09.

603. Wengrower D, Knobler H, Gillis S, Chajek-Shaul T. Myocarditis in tick-borne relapsing fever. *J Infect Dis* 1984; **149**: 1033.

604. Goodman RL, Arndt KA, Steigbigel NH. *Borrelia* in Boston. *JAMA* 1969; **210**: 722–23.

605. Dewar HA, Walmsley R. Relapsing fever with nephritis and subarachnoid haemorrhage. *Lancet* 1945; **2**: 630.

606. Blazejak K, Raulf MK, Janecek E, et al. Shifts in *Borrelia burgdorferi* (s.l.) geno-species infections in *Ixodes ricinus* over a 10-year surveillance period in the city of Hanover (Germany) and *Borrelia miyamotoi*-specific Reverse Line Blot detection. *Parasit Vectors* 2018; **11**: 304.

607. Wodecka B, Rymaszewska A, Skotarczak B. Host and pathogen DNA identification in blood meals of nymphal *Ixodes ricinus* ticks from forest parks and rural forests of Poland. *Exp Appl Acarol* 2014; **62**: 543–55.

608. Burri C, Schumann O, Schumann C, Gern L. Are *Apodemus* spp. mice and *Myodes glareolus* reservoirs for *Borrelia miyamotoi*, *Candidatus Neoehrlichia mikurensis*, *Rickettsia helvetica*, *R. monacensis* and *Anaplasma phagocytophilum*? *Ticks Tick Borne Dis* 2014; **5**: 245–51.

609. Wilhelmsson P, Lindblom P, Fryland L, et al. Prevalence, diversity, and load of *Borrelia* species in ticks that have fed on humans in regions of Sweden and Aland Islands, Finland with different Lyme borreliosis incidences. *PLoS One* 2013; **8**: e81433.

610. Reeves WK, Loftis AD, Sanders F, et al. *Borrelia, Coxiella,* and *Rickettsia* in *Carios capensis* (Acari: Argasidae) from a brown pelican (*Pelecanus occidentalis*) rookery in South Carolina, USA. *Exp Appl Acarol* 2006; **39**: 321–29.

611. Hulínská D, Votýpka J, Kríz B, et al. Phenotypic and genotypic analysis of *Borrelia* spp. isolated from *Ixodes ricinus* ticks by using electrophoretic chips and real-time polymerase chain reaction. *Folia Microbiol (Praha)* 2007; **52**: 315–24.

612. Yang Xiao N, Yang Hui J, Zhang L, et al. Tick-borne pathogens in Shanxi Province, China. *Biomed Environ Sci*, 2021; **34**: 410–15.

613. Algwaiz LA, Almashhadani SA, Ayoola EA, et al. Relapsing fever in Saudi-Arabia - report of 2 cases. *Ann Saudi Med* 1995; **15**: 165–67.

614. Borda M. *Borrelia duttoni* infection without tick-bite. *Trans R Soc Trop Med Hyg* 1986; **80**: 495.

615. Young WA, Farr AG, Mc KA. Relapsing fever in the Lake Province of Tanganyika with an account of a case in an eight day old infant. *East Afr Med J* 1946; **23**: 345–47.

616. Walton GA. Relapsing fever in the Digo District of Kenya Colony. *East Afr Med J* 1955; **32**: 377–93.

617. Daniel E, Beyene H, Tessema T. Relapsing fever in children--demographic, social and clinical features. *Ethiop Med J* 1992; **30**: 207–14.

618. Trowell HC. The treatment of tick-borne relapsing fever in East Africa with special reference to aureomycin. *East Afr Med J* 1951; **28**: 402–12.

619. Titkov AV, Platonov AE, Stukolova OA, et al. Epidemiological features of *Ixodes* tick-borne borelioses in the Krasnoyarsk territory in the context of searching for the cases of infection caused by *Borrelia miyamotoi*. *Zh Mikrobiol Epidemiol Immunobiol*, 2018; **3**: 10–18.

620. Brinkmann A, Hekimoğlu O, Dinçer E, Hagedorn P, Nitsche A, Ergünay K. A cross-sectional screening by next-generation sequencing reveals *Rickettsia*, *Coxiella*, *Francisella*, *Borrelia*, *Babesia*, *Theileria* and *Hemolivia* species in ticks from Anatolia. *Parasit Vectors* 2019; **12:** 26.

621. Farone TS, Campagnolo ER, Mason KL, Butler CL. *Borrelia miyamotoi* infection rate in black-legged ticks (*Ixodes scapularis*) recovered from heads of hunter-harvested white-tailed deer (*Odocoileus virginianus*) in Pennsylvania: a public health perspective. *JPAS*, 2018; **92**: 1–12.

622. Fomenko NV, Epikhina TI, Chernousova NY. Identification *Borrelia miyamotoi* in the blood of people taken ill in the spring and summer. *Molekulyarnaya meditsina*, 2010; **3**: 28–31.

623. Godeluck B, Duplantier J-M, Ba K, Trape J-F. A longitudinal survey of *Borrelia crocidurae* prevalence in rodents and insectivores in Senegal. *Am J Trop Med Hyg* 1994; **50**: 165–68.

624. Krasnova EI, Savelyeva MV, Khokhlova NI, et al. Features of clinical manifestations and laboratory diagnosis of tick-borne relapsing fever caused by *Borrelia miyamotoi* in the Novosibirsk region. *Epidemiol Infect Dis* 2017; **2**: 10–15.

625. Kiewra D, Zalesny G. Relationship between temporal abundance of ticks and incidence of Lyme borreliosis in Lower Silesia regions of Poland. *J Vector Ecol* 2013; **38**: 345–52.

626. Klitgaard K, Kjaer LJ, Isbrand A, Hansen MF, Bodker R. Multiple infections in questing nymphs and adult female *Ixodes ricinus* ticks collected in a recreational forest in Denmark. *Ticks and Tick-Borne Dis* 2019; **10**: 1060–65.

627. Korotkov Iu S, Kislenko GS, Burenkova LA, Rudnikova NA, Karan LS. Spatial and temporal variability of *Ixodes ricinus* and *Ixodes persulcatus* infection with the Lyme disease agent in Moscow Region. *Parazitologiia* 2008; **42**: 441–51.

628. Lane RS, Manweiler SA. *Borrelia coriaceae* in its tick vector, *Ornithodoros coriaceus* (Acari: Argasidae), with emphasis on transstadial and transovarial infection. *J Med Entomol* 1988; **25**: 172–77.

629. Lee K. The role of sika deer (*Cervus nippon yesoensis*) in the transmission of *Borrelia* spp. in Hokkaido, Japan. *Jpn. J Vet Res* 2014; **62**: 199–200.

630. Lubova VA, Leonova GN, Shutikova AL. Role of *Ixodes* ticks in circulation of tick-borne infections in the south of the far east. *Ekologiya cheloveka* 2020; **2**: 58–64.

631. Melkert PW, Stel HV. Neonatal *Borrelia* infections (relapsing fever): report of 5 cases and review of the literature. *East Afr Med J* 1991; **68**: 999–1005.

632. Pineda Cantero A, Pérez de Pedro I, Martín Téllez S, et al. *Borrelia hispanica* as a cause of recurrent fever. *Med Clin (Barc)* 2020; **154**: 380.

633. Rar VA, Epikhina TI, Tikunova NV, et al. DNA detection of pathogens transmitted by Ixodid ticks in blood of small mammals inhabiting the forest biotopes in Middle Irtysh Area (Omsk Region, West Siberia). *Parazitologiia* 2014; **48**: 37–53.

634. Sambado S, Salomon J, Crews A, Swei A. Mixed transmission modes promote persistence of an emerging tick-borne pathogen. *Ecosphere* 2020; **11**: e03171.

635. ZUMPT F, ORGAN D. Strains of spirochaetes isolated from *Ornithodoros zumpti* Heisch & Guggisberg, and from wild rats in the Cape Province. *Afr J Lab Clin Med* 1961; **7**: 31–35.

636. Sarksyan DS, Platonov AE, Karan LS, et al. Clinical presentation of "new" tick-borne borreliosis caused by *Borrelia miyamotoi. Ter Arkh* 2012; **84**: 34–41.

637. Shutikova AL, Lubova VA, Leonova GN. Verification of mono- and mixed contaminations transmitting agents tick-borne infections. *Klin Lab Diagn* 2020; **65**: 659–64.

638. Singh J. Recurring fever and headache in a six-year-old child. *Pediatr. Infect Dis J* 1999; **18**: 842.

639. Abdullah H, Aboelsoued D, Farag TK, et al. Molecular characterization of some equine vector-borne diseases and associated arthropods in Egypt. *Acta Trop* 2022; **227**: 106274.

640. Abdullah H, Amanzougaghene N, Dahmana H, et al. Multiple vector-borne pathogens of domestic animals in Egypt. *PLoS Negl Trop Dis* 2021; **15**: e0009767.

641. Abdullah H, Elbayoumy MK, Allam AM, Ashry HM, Abdel-Shafy S. Molecular epidemiology of certain vector-borne bacterial microorganisms in domestic animals and their ectoparasites in Egypt. *Trop Anim Health Prod* 2021; **53**: 484.

642. Kang JG, Chae JB, Cho YK, et al. Molecular Detection of *Anaplasma, Bartonella*, and *Borrelia theileri* in Raccoon Dogs (*Nyctereutes procyonoides*) in Korea. *Am J Trop Med Hyg* 2018; **98**: 1061–68.

643. Aouadi A, Leulmi H, Boucheikhchoukh M, et al. Molecular evidence of tick-borne hemoprotozoan-parasites (*Theileria ovis* and *Babesia ovis*) and bacteria in ticks and blood from small ruminants in Northern Algeria. *Comp Immunol Microbiol Infect Dis* 2017; **50**: 34–39.

644. Bottieau E, Verbruggen E, Aubry C, Socolovschi C, Vlieghe E. Meningoencephalitis complicating relapsing fever in traveler returning from Senegal. *Emerg Infect Dis* 2012; **18:** 697-98.

645. Smith RD, Brener J, Osorno M, Ristic M. Pathobiology of *Borrelia theileri* in the tropical cattle tick, *Boophilus microplus*. *J Invertebr Pathol* 1978; **2**: 182–90.

646. Gioia GV, Vinueza RL, Marsot M, et al. Bovine anaplasmosis and tick-borne pathogens in cattle of the Galapagos Islands. *Transbound Emerg Dis* 2018; **65**: 1262–71.

647. Hagen RM, Frickmann H, Ehlers J, et al. Presence of *Borrelia* spp. DNA in ticks, but absence of *Borrelia* spp. and of *Leptospira* spp. DNA in blood of fever patients in Madagascar. *Acta Trop* 2018; **177**: 127–34.

648. Li ZM, Xiao X, Zhou CM, et al. Human-pathogenic relapsing fever *Borrelia* found in bats from central China phylogenetically clustered together with relapsing fever borreliae reported in the New World. *PLoS Negl Trop Dis* 2021; **15**: e0009113.

649. Mel'nikova OV, Trushina YN, Adel'shin RV, et al. The abundance of *Borrelia* in ixodid ticks of Baikal region. *Med Parazitol* 2021; **3**: 12–20.

650. Scoles GA, Lohmeyer KH, Ueti MW, et al. Stray Mexico origin cattle captured crossing into Southern Texas carry *Babesia bovis* and other tick-borne pathogens. *Ticks Tick Borne Dis* 2021; **12**: 101708.

651. Sharma SP, Amanfu W, Losho TC. Bovine borreliosis in Botswana. *Onderstepoort J Vet Res* 2000; **67**: 221–23.

652. Young AS, Mutugi JJ, Kariuki DP, et al. Immunisation of cattle against theileriosis in Nakuru District of Kenya by infection and treatment and the introduction of unconventional tick control. *Vet Parasitol* 1992; **42**: 225–40.

653. Norton JR, Steiert JG. Steiert Infection rates of *Amblyomma americanum* and *Dermacentor variabilis* by *Francisella tularensis* and *Borrelia lonestari* in Southwest Missouri. *Vector Borne Zoonotic Dis* 2002; **2:** 53-60.

654. Qiu Y, Squarre D, Nakamura Y, et al. Evidence of *Borrelia theileri* in wild and domestic animals in the Kafue Ecosystem of Zambia. *Microorganisms* 2021; **9**: 2405.

655. Padgett K, Bonilla D, Kjemtrup A, et al. Large scale spatial risk and comparative prevalence of *Borrelia miyamotoi* and *Borrelia burgdorferi* sensu lato in *Ixodes pacificus*. *PLoS One* 2014; **9**: e110853.

657. Xie J. Clinical observation of 12 cases of tick-borne relapsing fever. *People's Military Surgeon* 1959; **2**: 109–10.

658. Yao MG. Investigation on tick-borne relapsing fever spirochete in Yunnan Province. Master, Dali University; 2020.

659. Zhu LX, Gong GY. Relapse-fever spirochetes were detected in cerebrospinal fluid of a patient with meningitis. *Chinese Journal of Infectious Dis* 1994; **12**: 144.

660. Wang MD, Shao GN. Lessons from epidemic tick-borne relapsing fever outbreaks in camping. *People's Military Surgeon* 1959; **6**: 442–43.

661. Mitchell EA, Williamson PC, Billingsley PM, et al. Frequency and distribution of Rickettsiae, Borreliae, and Ehrlichiae detected in human-parasitizing ticks, Texas, USA. *Emerg Infect Dis* 2016; **22**: 312–15.

662. Keller C, Zumblick M, Streubel K, et al. Hemorrhagic diathesis in *Borrelia recurrentis* infection imported to Germany. *Emerg Infect Dis* 2016; **22**: 917–19.

663. Porcella SF, Raffel SJ, Schrumpf ME, et al. Serodiagnosis of louse-borne relapsing fever with glycerophosphodiester phosphodiesterase (GlpQ) from *Borrelia recurrentis*. *J Clin Microbiol* 2000; **38**: 3561–71.

664. Cutler SJ, Bonilla EM, Singh RJ. Population structure of East African relapsing fever *Borrelia* spp. *Emerg Infect Dis* 2010; **16**: 1076–80.

665. Hendson M, Lane RS. Genetic characteristics of *Borrelia coriaceae* isolates from the soft tick *Ornithodoros coriaceus* (Acari: Argasidae). *J Clin Microbiol* 2000; **38**: 2678–82.

666. Sanz-Aguilar A, Payo-Payo A, Rotger A, et al. Infestation of small seabirds by *Ornithodoros* *maritimus* ticks: Effects on chick body condition, reproduction and associated infectious agents. *Ticks Tick Borne Dis* 2020; **11**: 101281.

667. Shariat BM, Freimund JA, Wright SM, Murphree CS, Thomas JT. *Borrelia* infection rates in winter ticks (*Dermacentor albipictus*) removed from white-tailed deer (*Odocoileus virginianus*) in Cheatham County, Tennessee. *J Tenn Acad Sci* 2007; **82**: 57–61.

668. Fomenko NV, Livanova NN, Borgoiakov V, et al. Detection of *Borrelia miyamotoi* in ticks *Ixodes persulcatus* from Russia. *Parazitologiia* 2010; **44**: 201–11.

669. Bacon RM, Pilgard MA, Johnson BJ, Piesman J, Biggerstaff BJ, Quintana M. Rapid detection methods and prevalence estimation for *Borrelia lonestari* glpQ in *Amblyomma americanum* (Acari: Ixodidae) pools of unequal size. *Vector Borne Zoonotic Dis* 2005; **2**: 146–56.

670. Mendell NL, Reynolds ES, Blanton LS, et al. Detection of Rickettsiae, Borreliae, and Ehrlichiae in ticks collected from Walker County, Texas, 2017-2018. *Insects* 2019; **10**: 315.

671. Taft SC, Miller MK, Wright SM. Distribution of borreliae among ticks collected from eastern states. *Vector Borne Zoonotic Dis* 2005; **4**: 383–89.

672. Varela AS, Moore VA, Little SE. Disease agents in *Amblyomma americanum* from northeastern Georgia. *J Med Entomol* 2004; **4**: 753–59.

673. Cordeiro MD, Bahia M, Magalhães-Matos PC, et al. Morphological, molecular and phylogenetic characterization of *Borrelia theileri* in *Rhipicephalus microplus*. *Rev Bras Parasitol Vet* 2018; **27**: 555–61.

674. Armstrong PM, Rich SM, Smith RD, et al. A new *Borrelia* infecting Lone Star ticks. *Lancet* 1996; **347**: 67–68.

675. Ataliba AC, Resende JS, Yoshinari N, Labruna MB. Isolation and molecular characterization of a Brazilian strain of *Borrelia anserina*, the agent of fowl spirochaetosis. *Res Vet Sci* 2007; **2**: 145–49.

676. Fukunaga M, Ushijima Y, Aoki Y, Talbert A. Detection of *Borrelia duttonii*, a tick-borne relapsing fever agent in central Tanzania, within ticks by flagellin gene-based nested polymerase chain reaction. *Vector Borne Zoonotic Dis* 2001; **1**: 331–38.

677. Lin T, Gao L, Seyfang A, Oliver JH. ‘*Candidatus* Borrelia texasensis’, from the American dog tick *Dermacentor variabilis*. *Int J Syst Evol Microbiol* 2005; **55**: 685–93.

678. Rafyi A, Stiller D, Maghami G. Some observations on *Ornithodoros parkeri* from Monterey County and its associated *Borrelia* infection. *Bull Soc Pathol Exot Filiales* 1965; **58**: 1157–69.

679. Reeves WK, Loftis AD, Sanders F, et al. *Borrelia, Coxiella,* and *Rickettsia* discovered from *Carios capensis* collected from South Carolina, USA. *Exp Appl Acaro*, 2005; **39**: 321–29.

680. Jordan BE, Onks KR, Hamilton SW, Hayslette SE, Wright SM. Detection of *Borrelia burgdorferi* and *Borrelia lonestari* in birds in Tennessee. *J Med Entomol* 2009; **46**: 131-38.

681. Shah AH, Khan MN, Iqbal Z, Sajid MS, Akhtar MS. Some epidemiological aspects and vector role of tick infestation on layers in the Faisalabad district (Pakistan). *World Poultry Sci J* 2006; **62**: 145–57.

682. Uhlmann EJ, Seed PC, Schwan TG, Storch GA. Polymerase chain reaction of tick-borne relapsing fever caused by *Borrelia hermsii*. *Pediatr. Infect Dis J* 2007; **26**: 267–69.

683. Tanner CL, Ammer FK, Barry RE, Stromdahl EY. Tick burdens on *Peromyscus leucopus* *Rafinesque* and infection of ticks by *Borrelia* spp. in Virginia. *Southeastern Naturalist* 2010; **9**: 529–46.

684. Tucker WA. A report on the treatment of tick relapsing fever with sodium penicillin. *East Afr Med J* 1946; **23**: 13–18.

685. Varela AS, Luttrell MP, Howerth EW, et al. First culture isolation of *Borrelia lonestati*, putative agent of southern tick-associated rash illness. *J Clin Microbiol* 2004; **42**: 1163–69.

686. Van Heerden J, Reyers F. Reyers. *Borrelia* sp. infection in a horse. *J S Afr Vet Assoc* 1984; **55**: 41–43.

687. Sa'idu L, Agbede RIS, Abdu AP. Prevalence of avian spirochaetosis in Zaria (1980-1989). *Isr J Vet Med* 1995; **50**: 39–40.

688. Wilting KR, Stienstra Y, Sinha B, et al. Louse-borne relapsing fever (*Borrelia recurrentis*) in asylum seekers from Eritrea, the Netherlands, July 2015. *Euro Surveill* 2015; **20**: 21196.

689. Shao GN. Methods on investigation of the epidemic focus of tick-borne relapsing fever. *People's Military Surgeon* 1982; **3**: 17–19.

690. Han HJ. Molecular biological investigation of pathogenic microorganisms carried by bats in Mengyin area of Shandong Province. Master, Wuhan University; 2019.

691. Zhou L. Investigation on pathogen carried by vector ticks in Inner Mongolia forest region. Master, Academy of Military Medical Sciences; 2017.

692. Vasil'eva IS, Ershova AS, Mansurov AA, et al. Changes in the village foci of tick-borne relapsing fever in Uzbekistan over a 10-year period. *Parazitologiia* 1991; **4**: 323–29.

693. Muñoz-Leal S, Lopes MG, Marcili A, Martins TF, González-Acuña D, Labruna MB. *Anaplasmataceae, Borrelia* and *Hepatozoon* agents in ticks (Acari: Argasidae, Ixodidae) from Chile. *Acta Trop* 2019; **192**: 91–103.

694. Mazzotti L. Sobre una nueva espiroqueta de la fiebre recurrente, encontrada en México. *Rev Inst Salub y Enf trop* 1949; **10**: 277–81.

695. DAVIS GE. A relapsing fever spirochete, *Borrelia mazzottii* (sp. nov.) from *Ornithodoros talaje* from Mexico. *Am J Hyg* 1956; **1**: 13–17.

696. CALERO C. Relapsing fever on the Isthmus of Panama; report of 106 cases. *Am J Trop Med Hyg* 1946; **6**: 761–69.

697. Emilio J. Pampana, M.D., D.T.M.&H. Notes on Colombian relapsing fever. *Trans R Soc Trop Med Hyg* 1928; **21**: 315–28.
